# Differential gene expression in male and female rainbow trout embryos prior to the onset of gross morphological differentiation of the gonads

**DOI:** 10.1186/1471-2164-12-404

**Published:** 2011-08-08

**Authors:** Matthew C Hale, Peng Xu, Julie Scardina, Paul A Wheeler, Gary H Thorgaard, Krista M Nichols

**Affiliations:** 1 Department of Biological Sciences, Purdue University, West Lafayette, IN 47907,USA; 2 School of Biological Sciences and Center for Reproductive Biology, Washington State University, Pullman, WA 99164, USA; 3 Department of Forestry and Natural Resources, Purdue University, West Lafayette, IN 47907, USA

**Keywords:** Sex-biased expression, ovary, testis, microarray, candidate genes

## Abstract

**Background:**

There are large differences between the sexes at the genetic level; these differences include heterogametic sex chromosomes and/or differences in expression of genes between the sexes. In rainbow trout (*Oncorhynchus mykiss*) qRT-PCR studies have found significant differences in expression of several candidate sex determining genes. However, these genes represent a very small fraction of the genome and research in other species suggests there are large portions of the transcriptome that are differentially expressed between the sexes. These differences are especially noticeable once gonad differentiation and maturation has occurred, but less is known at earlier stages of development. Here we use data from a microarray and qRT-PCR to identify genes differentially expressed between the sexes at three time points in pre-hatch embryos, prior to the known timing of sexual differentiation in this species.

**Results:**

The microarray study revealed 883 differentially expressed features between the sexes with roughly equal numbers of male and female upregulated features across time points. Most of the differentially expressed genes on the microarray were not related to sex function, suggesting large scale differences in gene expression between the sexes are present early in development. Candidate gene analysis revealed *sox9*, *DMRT1*, *Nr5a1 *and *wt1 *were upregulated in males at some time points and *foxl2*, *ovol1*, *fst *and *cyp19a1a *were upregulated in females at some time points.

**Conclusion:**

This is the first study to identify sexual dimorphism in expression of the genome during embryogenesis in any fish and demonstrates that transcriptional differences are present before the completion of gonadogenesis.

## Background

Genetic differences between the sexes can broadly be separated into two groups: differences in the transcription level, where the abundance of a particular gene transcript(s) differs between the sexes (a phenomenon known as sex-biased expression), and heterogametic sex chromosomes that are present in one sex and absent in the other. These two mechanisms can occur together, and often species that lack differentiated sex chromosomes exhibit sex specific gene expression [1,2]. Many studies have shown that sex-biased differences in gene expression are present after sex determination and differentiation has taken place; sex bias in gene expression has been documented in multiple species including fruit flies (*Drosophila sp*) [3-5], the worm (*Caenorhabditis elegans*) [6], the mouse (*Mus mus*) [7], chicken (*Gallus gallus*) [8,9], the flour beetle (*Tribolium *castaneum) [10] and zebra fish (*Danio rerio*) [11,2] (See [12] for review). Most (but by no means all) studies have found male bias in gene expression, with more genes upregulated in mature males than mature females [e.g. [5,7,2]]. This is hypothesized to be due to strong sexual selection in males in the form of female choice, and/or sperm competition [12,13].

Patterns of sex-biased gene expression are extremely variable both within and between species, and there are marked differences in the proportion, number, and identity of genes that are differentially expressed depending on the tissue type and developmental stage examined. For example 4% of the adult whole-body transcriptome exhibited sex-biased expression in mice [14] to as high as 88% in *Drosophila *[15]. Sex-biased expression also varies within different tissues of the same individual. For example, Yang et al. [7] studied sex-biased expression in *Mus *and found that brain tissue exhibits fewer sex-biased genes (13%) than muscle (55%), adipose (68%) and liver (72%) tissue. Other studies (on model organisms like *Drosophila *and zebra fish) report similar differences with the greatest percentage of sex-biased expression frequently being in the gonad transcriptome of sexually mature adults [e.g. [16,7,2]].

Although sex-bias in expression seems to be a common phenomenon in many different species, most studies have used sexually mature specimens or individuals (i.e. juveniles) that have already undergone differentiation of the gonads (either whole individuals or tissue specific transcriptomes). However, substantially less information is available on the level of sex-biased expression in un-differentiated embryos. Studies in the pre-implantation stage of embryogenesis in mouse found that roughly 3% of the transcriptome is differentially expressed between the sexes [17,18]. In chicken, levels of sex-biased expression in embryos after differentiation have been compared with adults, and unsurprisingly found far less sex-biased expression in embryos [9]. This suggests that the amount of sex-biased expression in the transcriptome changes throughout development through the juvenile and adult stages, and that sex-biased expression patterns may be present very early in development. The extent to which biases in gene expression occur prior to gonad differentiation, however, have not been fully explored.

Genetic differences between the sexes also occur due to the presence of heteromorphic sex chromosomes. Genetic sex determination (GSD) is most often thought of as being initiated by a switch in one sex that begins the gonad differentiation cascade [see [19] for review]. However, despite the widespread importance of genetic sex determination across taxonomic groups, surprisingly little is known about the genes involved in the cascade in fishes [see [20-23] for review]. Unlike mammals (for which the gene *SRY *is the primary switch that initiates the cascade [24]), fish species with GSD appear to use a range of different loci, with *DMY *in medaka the only known example of a primary switch [25,26]. Karyotype and inheritance studies have shown there are a number of fish species that have GSD, but the loci responsible have yet to be determined. Sex can also be determined by a set of genes presumably on both the sex chromosomes and the autosomes that act in concert to determine sex, e.g. many species of livebearers including the platyfish *Xiphophorus maculatus *[[27]; reviewed in [21]]. Rainbow trout (*Oncorhynchus mykiss*) are known to have GSD, but the mechanisms triggering the differentiation of testes and ovaries during development have not yet been revealed. However, a number of studies have identified candidate sex determination genes that are differentially expressed as early as the onset of exogenous feeding, and presumably during the differentiation process [28,29], but no studies have yet identified the sex determination gene on the Y chromosome. Moreover, these studies evaluated candidate genes known to be important for sex determination in other species. A whole-transcriptome approach may be better suited for determining as-yet unidentified sex determination genes, while also revealing the patterns of sex bias in gene expression at the earliest time points in development.

Our aims here were twofold: 1) to evaluate differences in gene expression between the sexes in pre-hatch embryos of rainbow trout on a genome-wide level using a microarray analysis to: a) determine how much of the transcriptome appears to be differentially expressed between the sexes early on in development (i.e. identify sex bias in gene expression on a global level), and b) identify genes which are known to be involved in the sex differentiation cascade in model organisms, and 2) to characterize the expression profiles of a set of candidate sex genes that are known to be differentially expressed at the onset of exogenous feeding (swim up) in an attempt to ascertain the time point earlier in development when these genes are being turned on. This study focuses on three development time points: 15 days post-fertilization (dpf; eyed stage; 165 degree days), 19 dpf (caudal flexing stage; 209 degree days) and 28 dpf (beginning of hatch stage; 308 degree days). This combination of approaches identifies sex bias in gene expression at some of the earliest time points in development on a genome-wide level, even before the onset of sex determination and phenotypic sex differentiation, while also evaluating the timing of genes known for their role in the sex determination cascade in other species.

## Results

Samples used in this experiment were from a fourth generation backcross between two clonal lines of rainbow trout (*Oncorhynchus mykiss*), as described in Xu et al. [30]. One line (Clearwater, CW, YY male) possessed alleles for fast development, and the other line (Oregon State University, OSU, XX female) possessed alleles for slower development at a major QTL for embryonic development rate. Here we use samples from this cross to investigate sex differences in the expression in the early stages of development of *O. mykiss*.

### Microarray data results of differential expression between the sexes

Microarray data from the experiments have been submitted to the Gene Expression Omnibus database http://www.ncbi.nlm.nih.gov/geo/ according to MIAME guidelines. The series accession number is GSE13570. Controlling for the false discovery rate in multiple tests, 883 features were identified as differentially expressed between female and male embryos in at least one comparison. More features were differentially expressed between the sexes in samples from OSU/CW than OSU/OSU; these included 208 features from OSU/CW in 15 dpf embryos compared to 163 from OSU/OSU, 507 features in 19 dpf embryos from OSU/CW compared to 202 features from OSU/OSU, and 138 features in 28 dpf embryos from OSU/CW compared to 98 from OSU/OSU.

To focus on features with large expression differences, there were 276 (32% of differentially expressed genes) features with a minimum of a 3-fold change expression difference between the sexes in at least one comparison. The top 15 features with the greatest differences in expression at 15, 19 and 28 dpf are shown in Tables 1, 2 and 3 (see Additional File 1 for complete list of genes). Of the features differentially expressed at 15 dpf, 48 features were higher expressed in males, and 51 were greater expressed in females. The most common GO terms associated with these features were cell cycle regulation (36.8%), muscle contraction and development (13.2%) and transport (9.2). In the 19 dpf samples 85 features were higher expressed in males, and 45 were higher expressed in females. The most common GO terms were cell cycle regulation (30.1%), protein metabolism (11.7%) and lipid metabolism (10.7%). In the 28 dpf samples 26 features were upregulated in males and 21 features were upregulated in females. The most common GO terms were cell cycle regulation (35.5%), protein synthesis (12.9%) and transport (12.9%).

**Table 1:** Top 15 features with greatest difference in expression between the males and females rainbow trout embryos (15 dpf), genes above the line are upregulated in females, below the line upregulated in males

ID	Blast hit	F/M	P-value
**Female-upregulated genes**			
CA044503	Small inducible cytokine B14 precursor	13.59	1.96E-03
CA063549	Apolipoprotein F precursor	10.15	3.23E-04
CB510226	Parvalbumin-2	7.51	9.16E-03
CB493442	Sorcin	6.40	3.29E-03
CB496453	Gap junction beta-4 protein	6.27	5.94E-03
CA039346	Ornithine aminotransferase, mitochondrial precursor	6.05	2.21E-05
CB509706	Parvalbumin-2	5.97	1.35E-02
CB504468	Elastase-1	5.44	6.30E-04
CA060056	Ornithine decarboxylase antizyme 2	4.78	4.41E-03
CB510525	Guanine nucleotide-binding protein G(t) subunit alpha	4.36	1.13E-02
CA038163	Complement C3-1	4.34	2.43E-03
CB510736	DNA-binding protein inhibitor ID-2	4.20	4.08E-03
CK991090	Glycerol-3-phosphate dehydrogenase 1-like protein	4.02	7.79E-03
CB496738	mRNA-binding protein expressed during iron starvation	3.80	5.48E-03

**Male upregulated genes**			
CB517495	Nuclear pore complex protein Nup88	0.08	1.56E-03
CB510281	Parvalbumin beta 1	0.10	5.76E-03
CA038193	Fatty acid-binding protein, liver	0.14	9.81E-05
CB498670	Proteasome maturation protein	0.16	6.17E-04
CA051876	60S ribosomal protein L19	0.16	3.03E-04
CK990989	Elongation factor 1-alpha, oocyte form	0.17	1.38E-02
CB498109	SWI/SNF-related matrix-associated actin-dependent regulator of chromatin subfamily E member 1-related	0.19	9.28E-03
CB515883	Sodium/potassium-transporting ATPase subunit beta-233	0.20	2.41E-03
CB503485	BTB/POZ domain-containing protein KCTD5	0.21	3.65E-04
CA051876	60S ribosomal protein L19	0.21	1.06E-02
CA045072	Staphylococcal nuclease domain-containing protein 1	0.22	3.10E-02
CB511669	Protein BCCIP homolog	0.22	2.02E-03
CA769320	Fatty acid-binding protein, intestinal	0.23	5.93E-03
CA047068	Sodium/potassium-transporting ATPase subunit beta-3	0.23	8.94E-03
CK990989	Elongation factor 1-alpha, oocyte form	0.23	4.85E-03

**Table 2:** Top 15 annotated features with a three-fold difference in expression between female and male rainbow trout embryos (19 dpf), genes above the line are upregulated in females, genes below the line are upregulated in males

ID	Blast hit	F/M	P-value
**Female upregulated genes**			
CA042004	High-affinity copper uptake protein 1	5.38	2.73E-04
CA051515	Cellular retinaldehyde-binding protein	5.32	2.47E-03
CK991151	Transcription factor HES-1	4.94	7.97E-02
CB496523	15-hydroxyprostaglandin dehydrogenase [NAD+]	4.66	3.94E-03
CA053442	Medium-chain specific acyl-CoA dehydrogenase, mitochondrial precursor	4.50	8.81E-01
CB501058	putative acyl-CoA dehydrogenase	4.41	4.76E-04
CA046470	Oncorhynchus mykiss CD59-like protein (CD59) mRNA, complete cds	4.27	5.16E-03
CB515011	Galectin-3-binding protein precursor	4.21	1.39E-02
CA045033	Trypsin-1 precursor	4.20	5.48E-01
CA037513	Glutathione peroxidase 2	4.09	4.84E-01
CA038646	Periostin precursor	4.02	1.27E-03
CB494589	Glycogen phosphorylase, muscle form	3.96	2.79E-02
CA048635	Peroxisomal NADH pyrophosphatase NUDT12	3.87	9.97E-04
CK990305	Protein RCC2 homolog	3.86	5.53E-01
CA049909	Developmentally-regulated RNA-binding protein 1	3.74	1.80E-04

**Male up regulated genes**			
CA045222	PREDICTED: similar to MGC82565 protein isoform 1 [Danio rerio]	0.15	3.56E-03
CA055654	Arachidonate 5-lipoxygenase	0.16	4.37E-03
CA039066	Tripeptidyl-peptidase 1 precursor	0.16	1.06E-01
CB509577	Prolargin precursor	0.17	1.16E-02
CA058492	novel protein [Xenopus tropicalis]	0.17	4.09E-03
CB510525	Guanine nucleotide-binding protein G(t) subunit alpha	0.17	1.15E-01
CA051319	PAX interacting	0.18	1.31E-02
CA056706	Tektin-4	0.18	1.43E-03
CA058670	Stabilin-2 precursor	0.19	3.46E-04
CA044989	Plastin-1	0.19	4.54E-03
CB497295	NADH dehydrogenase [ubiquinone] 1 beta subcomplex subunit 2, mitochondrial precursor	0.19	1.10E-02
CA051876	60S ribosomal protein L19	0.20	3.79E-02
CB494343	Adenosylhomocysteinase B	0.21	8.60E-03
CB510525	Guanine nucleotide-binding protein G(t) subunit alpha	0.21	5.48E-03
CA058492	novel protein [Xenopus tropicalis]	0.22	1.33E-01

**Table 3:** Subset of non-redundant, annotated features with a three-fold difference in expression between female and male rainbow trout embryos (28 dpf)

ID	Blast hit	F/M	P-value
**Female upregulated**			
CB515363	Transmembrane protein 35	9.77	6.87E-03
CA050193	Granulins precursor	4.65	1.22E-02
CK991016	40S ribosomal protein SA	4.33	4.46E-03
CB509406	Phosphoribosyl pyrophosphate synthetase-associated protein 1	4.04	4.43E-04
CA046429	Pituitary tumor-transforming gene 1 protein-interacting protein precursor	3.76	1.64E-03
CB515607	Eukaryotic initiation factor 4A-I	3.51	6.07E-04
CB488623	Fatty acid-binding protein 1, liver	3.51	4.67E-03
CA044543	Apolipoprotein D precursor	3.30	3.81E-04
CA768033	Coatomer subunit alpha	3.23	9.30E-03
CB507253	pfam00909, Ammonium_transp, Ammonium Transporter Family	3.19	2.20E-02
CB504468	Elastase-1	3.14	1.34E-02
CB496736	Cytochrome c oxidase subunit 5A, mitochondrial precursor	3.09	4.76E-03
CB488623	Fatty acid-binding protein 1, liver	2.94	1.32E-02
CB496795	Serine/threonine-protein kinase Sgk3	2.91	8.97E-03
CA042983	Diamine acetyltransferase 1	2.79	4.43E-03

**Male upregulated**			
CA769703	Ubiquinol-cytochrome-c reductase complex core protein 2, mitochondrial precursor	0.07	2.29E-03
CB492678	Profilin-2	0.09	1.18E-02
CA058992	Salmo salar aryl hydrocarbon receptor 2b (AhR2) gene, exons 5 and 6	0.09	1.90E-04
CB503189	60S ribosomal protein L12	0.11	8.40E-03
CB503189	60S ribosomal protein L12	0.12	1.17E-02
CB516729	ER lumen protein retaining receptor 2	0.12	5.16E-03
CB516729	ER lumen protein retaining receptor 2	0.15	9.97E-03
CK990246	Phosphatidic acid phosphatase type 2 domain-containing protein 1B	0.18	1.10E-02
CA058992	Salmo salar aryl hydrocarbon receptor 2b (AhR2) gene, exons 5 and 6	0.19	6.41E-03
CA050997	40S ribosomal protein S3a	0.22	5.91E-03
CA044542	Hypoxia up-regulated protein 1 precursor	0.24	6.33E-03
CB499653	Enhancer of mRNA-decapping protein 4	0.25	1.30E-02
CK991256	Subunit of the THO complex	0.25	2.59E-03
CA063234	Cornichon homolog 4	0.26	8.58E-05
CB509577	Prolargin precursor	0.26	9.78E-02
CA037885	Cytochrome c oxidase polypeptide VIa, mitochondrial precursor	0.26	2.92E-03

**Figure 1:**
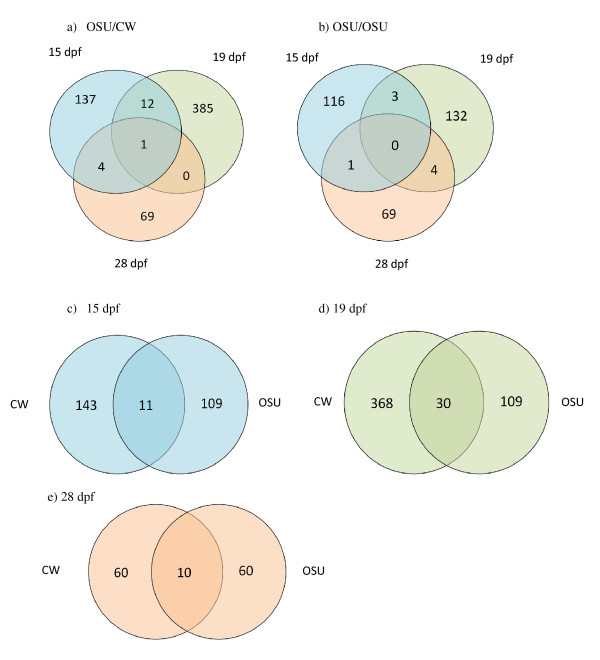
**Venn diagrams showing the number of significant sex-biased genes in common between a) all three time points in samples from OSU/CW, b) all three time points from OSU/OSU samples, c) Samples from 15 dpf from both OSU/CW and OSU/OSU, d) samples from 19 dpf, e) samples from 28 dpf**.

**Figure 2:**
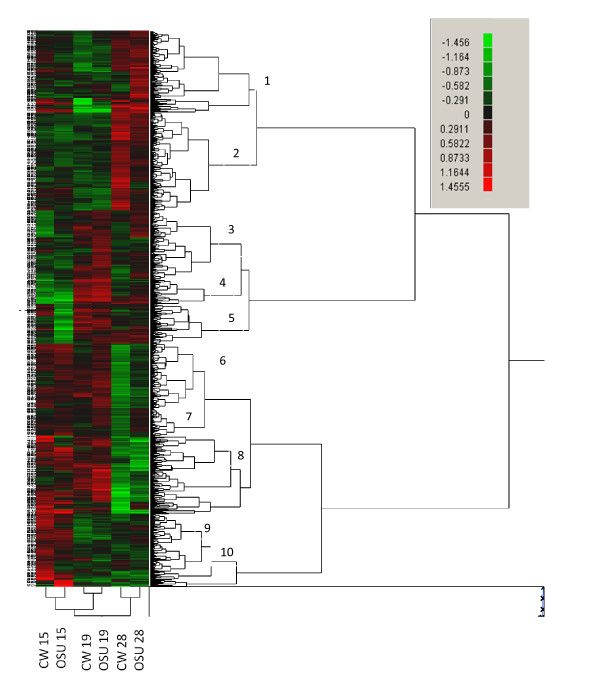
**Heat map produced by cluster analysis in JMP Genomics**. Data are presented as female to male ratios. Features in green are upregulated in males, features in red are upregulated in females. The cluster analysis found 10 discrete clusters and these are shown on the heat map.

Only a limited number of differentially expressed features between the sexes were shared between different time points; 17 out of 608 (3%) features in OSU/CW samples and 8 out of 325 (2.5%) in OSU samples (Figures 1a and 1b). (Note that there is overlap between features that were upregulated at different time points and between the two genotypes. In other words the 883 features is a non-redundant total). A small number of sex-biased features were shared between genotypes at the same time point with 11 out of 263 (4%) at 15dpf (Figure 1c), 30 out of 504 (6%) at 19 dpf (Figure 1d) and 10 out of 138 (7.2%) at 28 dpf (Figure 1e). These results indicate that very few differentially expressed genes are shared between genotypes, or between developmental time points, suggesting that expression patterns are rapidly changing during early stages of development, and that the individuals with the different QTL genotypes are developing at different rates [30]. Cluster analysis grouped the 883 features into 10 discrete clusters (Figure 2). Most of these clusters had only a few features, but clusters 2 and 6 had 164 and 133 features respectively. Cluster 2 grouped those features that were upregulated in OSU/CW females at 28 dpf, and features within cluster 6 showing upregulation in OSU/CW males at 28 dpf. Clustering analysis suggests that samples from the same developmental time point show more similar expression profiles than samples from different time points from the same genotype. For example, OSU/CW samples at 28 dpf are more similar to samples from to OSU/OSU at 28 dpf than OSU/CW samples from either 15 or 19 dpf. This trend is similar in samples from all three time points.

**Figure 3:**
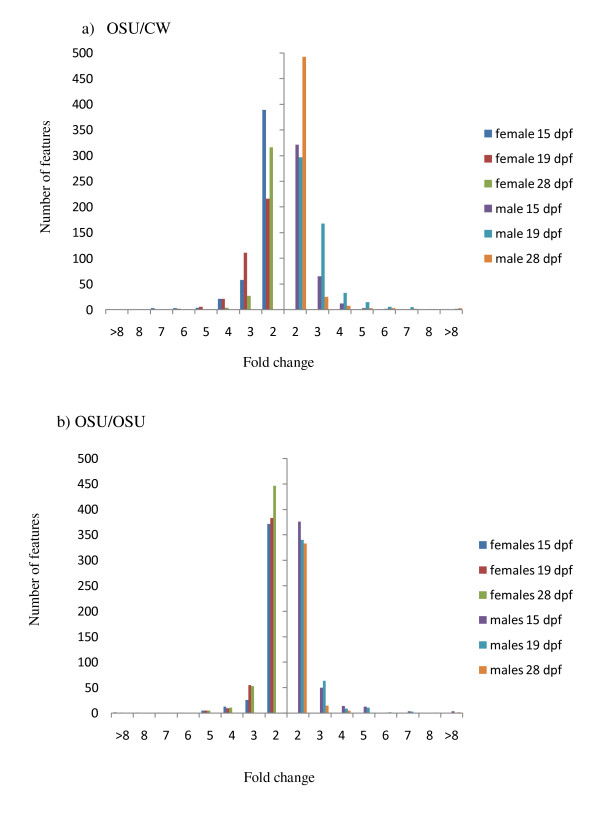
**Expression bias of 883 genes differentially expressed on the microarray**. Histogram showing the distribution of fold change values for female-enriched (left of Y axis bar) and male-enriched (right of Y axis bar) from a) OSU/CW, and b) OSU/OSU samples. Different colour bars represent different time points.

As mentioned, previous studies have documented a bias in the number of differentially expressed featured between the sexes [[12] for review]. We compared fold change values of differentially expressed features between the sexes to determine if there was bias in the directionality of differentially regulated genes between the sexes (Figure 3a and 3b). For each gene the fold change represents the magnitude and direction of differential expression between the sexes. There appears to be little difference in the number of genes differentially expressed between the sexes at 15 dpf in both genotypes, with marginally more female expressed genes than male expressed genes in OSU/CW (26 and 8 features in females, compared to 16 in males) (Figure 3a and 3b). There is a similar difference in the opposite direction in samples from OSU/OSU where 19 features have a three-fold upregulation in females compared to 28 in males. Samples from later developmental time points seem to show higher differences in fold expression between the sexes. At 19 dpf there are more features from both OSU/CW and OSU/OSU that exhibit male bias expression with 49 features with a three-fold difference from OSU/CW (compared to 28 from females) and 21 features with a three-fold difference from male OSU/OSU samples (compared to 16 from females). Very similar patterns were seen in samples from 28 dpf with males from both OSU/CW and OSU/OSU showing a greater number of three-fold difference genes than females (Figure 3a and 3b). The majority of the 883 sex-biased genes did not show big differences in expression between the sexes, and showed ratios less than 2:1.

### GO enrichment analyses

**Figure 4:**
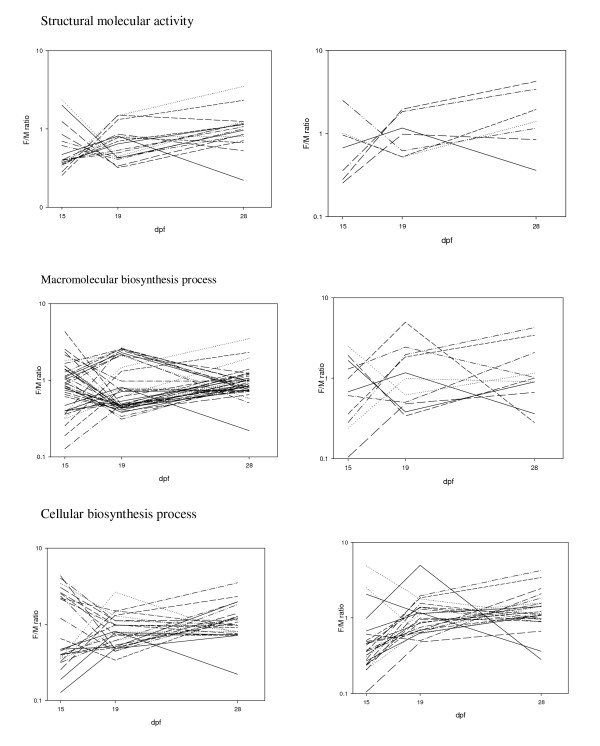
**Expression plots of three GO terms significantly differentially expressed**. Plots on the left show all three developmental time points from samples from OSU/CW, plots on the right from OSU/OSU. The three GO terms are a) structural molecular activity b) macromolecular biosynthesis process and c) cellular biosynthesis process.

GO enrichment analyses determined that only 3 GO terms identified from the 883 differentially expressed features between the sexes were upregulated compared to the whole 16K GRASP chip. These GO functions were cellular macromolecule metabolic process, (p = 0.003), macromolecule metabolic process (p = 0.01) and cellular protein metabolic process (p = 0.02). When comparing the features identified from one sex to the total set of differentially expressed genes, 62 GO terms were statistically different between the sexes (p = < 0.05). Of these 43 were differentially expressed in OSU/CW samples (Table 4) and 19 were differentially expressed in OSU/OSU samples (Table 5). For the OSU/CW samples, 24 GO terms were upregulated in males compared to 16 that were upregulated in females. For the OSU/OSU samples, 17 GO terms were upregulated in males compared to 2 GO terms in females. These differences suggest that in addition to more features being upregulated in males, more GO terms (and therefore more functional processes) are also upregulated in males compared to females. The ratio of GO terms upregulated in males compared to females changed between the three developmental time points. For example, within OSU/CW samples there were 19 GO terms upregulated in males compared to 3 GO terms in females at 15 dpf, 4 GO terms in males to 13 GO terms in females at 19 dpf and finally one GO term being upregulated in males at 28 dpf and none in females. These patterns suggest that the greatest differences in the functional categories differentially regulated between the sexes were at 15 and 19 dpf. Numbers of functional GO categories that were enriched from OSU/OSU were generally low but again the ratio of male to female upregulated genes varied between time points. All six GO terms differentially expressed at 15 dpf were upregulated in males, whereas the 2 GO terms differentially expressed between the sexes at 19 dpf were upregulated in females at 19 dpf. Some enriched GO categories were shared between different developmental time points and between different genotypes with some terms switching between upregulation in males and upregulation in females. For example structural molecular activity was upregulated in males at 15 dpf in samples from OSU/CW (p = 0.002), but upregulated in females at 19 dpf in OSU/CW (p = 0.001; Figure 4). However, some GO terms that were shared between the two genotypes showed similar patterns of expression such as cellular biosynthesis process, which was upregulated in males in samples from OSU/CW at 15 dpf (p = 0.03) and the same result was found in samples from OSU/OSU at 15 dpf (p = 0.03; Figure 4).

**Table 4:** GO terms identified as being significantly differentially expressed between the sexes within the OSU/CW genotype

dpf	GO term ID	GO term name	p-Value	females	males
15	GO:0005198	structural molecule activity	0.000	2	14
	GO:0009059	macromolecule biosynthetic process	0.001	2	12
	GO:0034645	cellular macromolecule biosynthetic process	0.001	2	12
	GO:0044249	cellular biosynthetic process	0.003	4	14
	GO:0003735	structural constituent of ribosome	0.003	1	9
	GO:0009058	biosynthetic process	0.006	5	14
	GO:0044260	cellular macromolecule metabolic process	0.010	9	18
	GO:0032991	macromolecular complex	0.011	13	22
	GO:0044430	cytoskeletal part	0.015	1	7
	GO:0003723	RNA binding	0.017	0	5
	GO:0003676	nucleic acid binding	0.017	9	17
	GO:0006412	translation	0.032	1	6
	GO:0005524	ATP binding	0.032	1	6
	GO:0032559	adenyl ribonucleotide binding	0.032	1	6
	GO:0006414	translational elongation	0.039	0	4
	GO:0016301	kinase activity	0.039	0	4
	GO:0016772	transferase activity	0.039	0	4
	GO:0044237	cellular metabolic process	0.043	16	22
	GO:0044424	intracellular part	0.048	34	37
	GO:0016491	oxidoreductase activity	0.021	11	2
	GO:0032502	developmental process	0.040	15	5
	GO:0050790	regulation of catalytic activity	0.047	5	0

19	GO:0043232	non-membrane-bounded organelle	0.000	16	0
	GO:0043228	non-membrane-bounded organelle	0.000	16	0
	GO:0005198	structural molecule activity	0.002	12	0
	GO:0032991	macromolecular complex	0.007	41	13
	GO:0003735	structural constituent of ribosome	0.011	9	0
	GO:0030529	ribonucleoprotein complex	0.013	15	2
	GO:0034645	cellular macromolecule biosynthetic process	0.013	12	1
	GO:0009059	macromolecule biosynthetic process	0.013	12	1
	GO:0005840	ribosome	0.018	8	0
	GO:0044237	cellular metabolic process	0.022	48	19
	GO:0044445	cytosolic part	0.030	7	0
	GO:0044249	cellular biosynthetic process	0.033	26	8
	GO:0006091	generation of precursor metabolites and energy	0.050	9	1
	GO:0003008	system process	0.050	6	0
	GO:0006412	translation	0.050	6	0
	GO:0042802	identical protein binding	0.050	6	0
	GO:0043231	intracellular membrane-bounded organelle	0.001	15	24
	GO:0043227	membrane-bounded organelle	0.001	15	24
	GO:0005576	extracellular region	0.034	1	5
	GO:0004872	receptor activity	0.043	3	7

28	GO:0005509	calcium ion binding	0.028	0	4

**Table 5:** GO terms identified as being significantly differentially expressed between the sexes within the OSU/OSU genotype

dpf	GO term ID	GO term name	p-Value	females	males
15	GO:0009058	biosynthetic process	0.014	2	17
	GO:0044424	intracellular part	0.018	11	38
	GO:0044249	cellular biosynthetic process	0.021	2	16
	GO:0003676	nucleic acid binding	0.029	1	12
	GO:0000166	nucleotide binding	0.031	2	15
	GO:0044281	small molecule metabolic process	0.044	2	14

19	GO:0048037	cofactor binding	0.016	5	0
	GO:0046483	heterocycle metabolic process	0.038	4	0

28	GO:0044444	cytoplasmic part	0.004	9	12
	GO:0044424	intracellular part	0.005	18	16
	GO:0032991	macromolecular complex	0.030	6	8
	GO:0015669	gas transport	0.031	0	3
	GO:0015671	oxygen transport	0.031	0	3
	GO:0046906	tetrapyrrole binding	0.031	0	3
	GO:0020037	heme binding	0.031	0	3
	GO:0044445	cytosolic part	0.031	0	3
	GO:0005344	oxygen transporter activity	0.031	0	3
	GO:0019825	oxygen binding	0.031	0	3
	GO:0005833	hemoglobin complex	0.031	0	3

Enrichment analysis on the 10 clusters shown in Figure 2 failed to identify any GO terms that were differentially expressed compared to both the complete (10,162 features) and sex-biased (883 features) datasets. This suggests that patterns of similar expression within clusters are not due to shared functionality. Thirty-eight GO terms were found to be significantly under expressed in 5 clusters (5, 7, 8, 9 and 10) but this is almost certainly due to small cluster size rather than a cluster of genes with shared function.

### qRT-PCR of candidate sex genes

We further examined whether genes with a potential role in sex determination were differentially expressed between male and female embryos. These genes were identified using two approaches: 1) Based on GO term analyses of differentially expressed genes on the microarray (details in methods), 9 candidate sex determining genes were identified for further study. Of these only 5 (*coatemer subunit*, *vasa*, *prostaglandin*, *cyp19a1a *and *zonadhesin*) amplified with qRT-PCR methods. 2) Of 102 candidate sex genes described in Baron et al [28] we investigated 15 genes (based on differences observed in Baron et al's study, and their known importance in the sex process in other vertebrates [28]). All 15 genes amplified product of the expected size (i.e. as described in [28]). Note that *cyp19a1a *(*aromatase*) was the only candidate sex gene identified from both the microarray and was in Baron et al's study.

**Figure 5:**
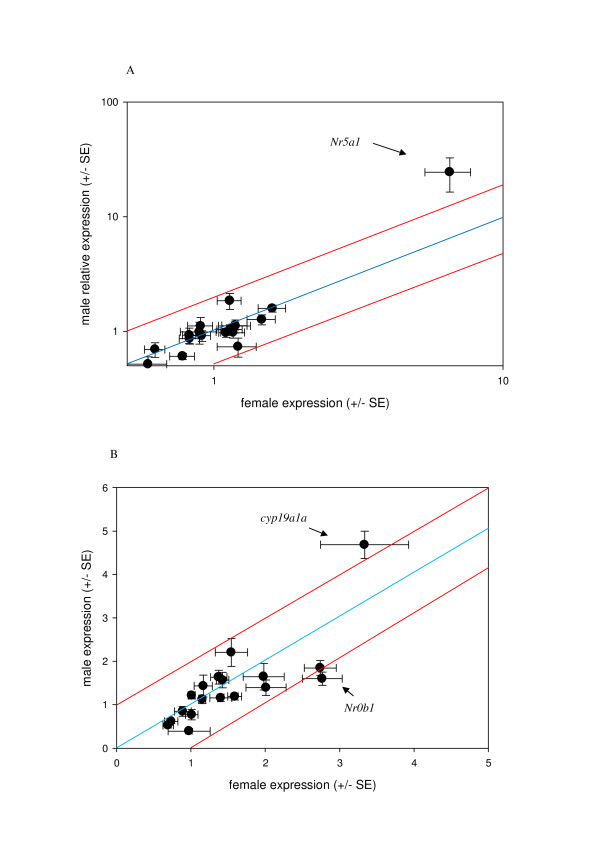
**Comparing female and male expression for sex determining genes**. Errors bars mark the SE of the mean for both female expression (x-axis) and male expression (y-axis). The blue line represents equal expression between the sexes, red lines represent 2-fold difference in expression. A) early developmental points (9, 15 and 19 dpf), and B) later development points (24, 28 and 33 dpf).

Both sets of genes were examined in the original three developmental time points, and a subset of six genes from Baron et al. [28] (*wt1*, *ovol1*, *foxl2A*, *foxl2B*, *DMRT1 *and so*x9b1*) as well as *zonadhesin *and *cyp19a1a *identified from the microarray were investigated at three additional developmental times points (8, 24 and 33 dpf). There is a common pattern in six of the genes (*wt1*, *ovol1*, *foxl2B*, *DMRT1 zonadhesin *and *cyp19a1a*) which show a higher peak of expression in 28 dpf (hatch) samples than the other developmental time points. At 28 dpf, the expression was higher in the males for *ovol1*, *wt1*, *foxL2B *and *DMRT1 *and higher in the females for *zonadhesin *and *cyp19a1a*. Looking at each time point only *ovol1 *shows a significant difference in expression between the sexes with higher expression in males than in females (p = 0.05). Sex was significant when it was considered as an interaction term with dpf (*wt1*, *ovol1*, *zonadhesin *and *cyp19a1a*) and with QTL (*ovol1*, *zonadhesin *and *cyp19a1a*). QTL was a significant factor in *ovol1*, *zonadhesin *and *cyp19a1a *although in all cases it was only marginally significant (Additional File 2). The other 10 sex genes did not show a significant relationship with sex either as a main or interaction term except *SOLT1 *where the sex*dpf interaction was significant (p = 0.02, Additional File 2). Most of these genes showed very similar levels of expression across all three developmental time periods (Figure 5, Additional Files 2 and 3).

Most of the candidate sex genes except for the ones discussed above show constant low levels of expression throughout all developmental time points and in both sexes (average correlation of all genes at each developmental time point; r^2 ^= 0.99 ± 0.003, p = <0.001) (see Additional File 4). Scatter plots shown in Figure 5 plot the mean relative expression values of the male and female samples per gene averaged between the early developmental time points (8-19 dpf; Figure 5a) and the late developmental time points (24-33 dpf; Figure 5b). Of note, *Nr5a1 *appears to be highly expressed in male samples from early developmental time points, whereas the other samples seem roughly similarly expressed between males and females. *ovol1 *has a two fold increase in expression in males than in females in later stages in development. *Cyp19a1a *and *Nr0b1 *show higher expression in females than in males at later stages of development.

### Candidate gene linkage mapping

Where possible, candidate sex genes were mapped to determine if any map to the putative sex determining region on the rainbow trout sex chromosomes. Four of the candidate sex determining genes (*sox9*, *DMRT1*, *Nr0b1 *and *Amh*) have already been mapped in rainbow trout, and so were not mapped here; none of these map to the sex chromosome [31]. Of the remaining (n = 11) candidate sex determining genes, three (*Nr5a1*, *GC1 *and *foxl2A*) failed to produce clean sequence product, despite primer redesign and optimization. This suggests that these genes are duplicated in the rainbow trout. Of the eight remaining genes all had at least one polymorphic base within the sequence and were genotyped in the OSU × CW doubled haploid mapping population [32,33]. One of these genes (*SOLT*) did not produce significant linkage with any other marker and remains in an unmapped region of the genome. None of the remaining seven genes mapped to the sex linkage group (LG1) (Additional File 5).

## Discussion

### Patterns of gene expression between the sexes

Repeatedly, microarray experiments have found that a substantial portion of the transcriptome is differentially expressed between the sexes [e.g. [2,5,10]]. Most of these studies have focused on model organisms such as *Drosophila*, *C. elegans *and *Mus*, with relatively few studies on non-model species that lack a complete genome sequence. Moreover, very few studies have included individuals that have not yet undergone sexual differentiation. Our study mined data from a microarray project to find a total of 883 genes that showed a significant difference in expression between the sexes before the completion of sexual differentiation. In so doing, it is not only one of the few studies to evaluate sex bias in the transcriptome in a non-model species, but is also one of the first studies to examine expression patterns between the genotypic sexes in embryos before the completion of gonad differentiation. Most genes differentially expressed between the sexes in this study are involved in processes such as cell cycle function and general structure, and are not necessarily associated with sexual development. This suggests that there are inherent differences in the transcriptome of male and female rainbow trout, and that these differences are present early in development. Other microarray studies in ovarian *O. mykiss *samples (collected from females of 1-2 years of age, after sexual differentiation) also found a wide range of genes differentially expressed in the ovaries, many of which had no obvious sex function [34]. These included transcripts that encode various elastases, cathepsins, proteases and immune function transcripts, suggesting that rainbow trout are exhibiting broad differences in transcriptome expression between the sexes throughout life. An expression quantitative trait loci (eQTL) study in an F1 cross between "dwarf" and "normal" Lake whitefish (*Coregonus clupeaformis*) found strong evidence for more eQTLs in females than in males. However, the genes underlying these eQTLs were found not to be differentially expressed between the sexes, strongly suggesting pleiotropic sex-linked effects in the transcriptome, at least within white muscle, the tissue used for microarray analysis [35].

Here we find a relatively low proportion of the transcriptome that exhibits sex bias in gene expression when compared to other studies, but it is important to consider the developmental stages of the samples. In our study, 8.7% of the features showed sex-biased gene expression (883 out of 10,153) in at least one of the three developmental time points in one of the two studied QTL genotypes. These numbers are low when compared to results in *Drosophila*, where a much higher percentage of sex-biased expression in mature adults (between 12% [4] and 88% [15] of genes) with most studies reporting a number near 50% [5]. Studies from whole individual adult *Tribolium *[10] and adult zebra fish [2] report around 20% and 38%, respectively. Similar numbers have been found in mice [e.g. [36]]; although note that like in *Drosophila*, sex-biased expression is very variable depending on tissue type with up to 72% of genes from liver to 13% of genes from brain [36]. Taken together these studies suggest that sex-biased gene expression is commonplace, however it seems to be variable both within and among species and variable with regard to tissue type and developmental stage.

There are a number of potential reasons for why we see fewer genes showing sex-biased expression compared to prior studies. Firstly, most other studies have focused on developmental stages that have occurred after sex differentiation, so presumably the samples used in such studies were actively expressing genes linked to sex and gamete functions [2,5]. In salmonids gonad differentiation tends to occur soon after the onset of hatching [37,38] which is around our oldest time point of sampling. So it is perhaps surprising that we did not find more sex-biased genes, and genes with an obvious sex function in samples from our oldest developmental point (28 dpf). Secondly, the absolute numbers of genes that are being expressed in embryos are less compared to the number of genes that are expressed in adults. Many of the sex and gamete function processes do not begin at such early stages of development and transcripts of such genes, presumably, would not be present (or present in very small numbers). Thirdly, the microarray is not a complete survey of the transcriptome and so there will be sex genes and sex-biased genes that were not surveyed. For example, none of the 16 candidate sex genes chosen for qRT-PCR study were on the microarray. Although these 16 genes did not show significant differences in expression between the sexes there are many other genes that have been shown to be differentially expressed at the onset of exogenous feeding in *O. mykiss *[28] that were not studied here. These reasons are not mutually exclusive, and all three undoubtedly help explain our results. Other studies have also found low levels of sex-bias expression in embryos, for example roughly 8.7% of the chicken embryo gonad transcriptome is female biased and 4.7% male biased [9]. This number increases to 33.5% and 28.2% in the adult gonads of females and males, respectively. Although the proportions of the transcriptome that was differentially expressed in chicken is similar to this study in rainbow trout, it is important to note key differences in the study designs. In our study, whole embryos were used from developmental time points prior to gross morphological differentiation of the gonads, while in [9] gonad tissue was used from embryos after sex differentiation.

Previous studies have examined sex-bias in gene expression to test hypotheses about the influence of sexual selection on gene expression. As other authors have suggested, a male bias in gene expression could provide support for a higher rate of functional evolution in genes important for sex and reproduction in males, where selective pressure on males in the form of male-male competition for mates is high [9,39,40]. We however, found roughly equal expression between the number of male biased and female biased genes, with only slightly higher number of male biased genes in samples from both lines at 19 dpf and slightly higher number of female biased genes in OSU/OSU at 15 dpf. Moreover, our enrichment analysis failed to identify any key functional processes with sex bias in gene expression and moreover within and among genes there was no consistent directionality to sex-biased in expression across time points (Figures 2 and 5a and 5b). All of the sex-biased genes and candidate sex determining genes showed spiky patterns of expression, suggesting rapid change in the transcriptome of the developing embryo. Many other studies using adult individuals report large differences in the number of sex-biased genes with, frequently, many more male-biased than female-biased genes [2,41], supporting the idea that sex bias towards males could be a product of sexual selection. However, studies in both *Drosophila *[5] and zebra fish [11] have found more female-biased genes than male-biased genes. These studies reinforce the idea that results can be very dependent on which transcriptomes are being investigated. For example, studies in the gonads [41] and whole body transcriptome [5] of *Drosophila *produce different results. In a hypothetical situation where sexual selection arises through sperm competition, sex bias may be expected to occur only at the level of the gonad and potentially swamped by signals from other tissues in whole body analyses (such as reported here). In contrast, sex bias in gene expression arising from selection in the form of male-male competition through alternative behaviors, ornamentation, or other 'phenotypes' outside of the gonads themselves could be found in the transcriptome of multiple tissues or at the level of the whole body. Moreover, the way in which animals used for transcriptome studies are maintained prior to tissue sampling can have a large effect. For example, [2] separated male and female zebra fish whereas [11] kept both sexes together immediately prior to sampling and in so doing, may have modified the expression of sex related genes. In other words, it is clear that the way individuals are reared prior to sampling can have an effect on the results of the experiment. This potentially could lead to misleading interpretations of the effects of sexual selection on sex-bias expression, as behavioral and physiological interactions among members of the opposite sex can elicit changes in gene expression. Finally, the degree to which gene expression early in life is genetically correlated to gene expression late in life when sexual selection is prominent also remain as questions to be explored in future studies of model and non-model organisms where sexual selection is prevalent.

### Candidate sex determination genes

Studies on post hatch rainbow trout indicate that genes known to be involved in the sex determination process are differentially expressed between the sexes [28,29]. However, we are still unclear as to when expression of these genes is initiated as the majority of the candidate sex determination genes were differentially expressed at the earliest point of sampling (55 dpf, onset of exogenous feeding; [28]). The *O. mykiss *embryos in our study were reared at 11°C and hatched at 28 dpf (or after 308 degree days), and therefore, our sampling procedure should span the point of sex determination. It has been found that differences in expression between the sexes in these genes tend to spike more at specific time points, rather than show consistent differences in multiple development points [28,29]. Our results show a similar trend in that out of 16 candidate sex genes only *ovol1 *showed a statistically significant difference in expression between male and female samples across all developmental time points sampled, although many genes showed differences in expression at specific time points. In the fruit fly (*Drosophila*) *ovol1 *is required for female germ line determination and differentiation [42]. *Ovol1 *is also expressed in sheep (*Ovis aries*) ovaries immediately prior to gonad differentiation [43]. Here we find upregulation of *ovol1 *in males at 15dpf and females at 28 dpf. Of the other genes that showed a significant difference between the sexes, *Nr5a1 *expression has been shown to be directly affected by *Sox9 *in mammals and *wt1 *in reptiles and is turned on after male determination [44,45]. With so few candidate genes showing differences between the sexes, it is important to give pause as to why this might be. Results from studies in mouse suggest that the window of differential expression of sex determining genes is small [e.g. [46-48]]. Perhaps we have missed the specific time point when these genes are upregulated? Increasing sampling effort just before the onset of hatch to the point of first exogenous feeding, and using a whole-transcriptome approach, could prove fruitful.

### Mapping of candidate sex differentiation genes

The fact that none of the candidate sex genes mapped to the sex chromosome in this study further suggests that a master control gene for the determination of sex in *O. mykiss *remains elusive. Linkage mapping of other candidate sex determining genes in other populations of *O. mykiss *also found no genes mapping to the sex chromosome. A total of 11 candidate sex genes have been mapped prior to this study, including 3 different transcripts of *wt1 *[49], three *sox9 *loci [31] and 2 *sox6 *genes, *Amh*, *DMRT1 *and *Nrob1 *[31]. The addition of *fst*, *ovo1*, *zonadhesin*, *IGF1*, *foxl2A*, *cyp19a1a *and *wt1 *brings the total number of loci to 18. The sex chromosomes of fishes (including salmonids) are at the early stages of differentiation (unlike mammals) and this in part helps explain why only one sex determining gene is known in any fish (*Dmy *in Medaka, [25]). In order to identify the master sex gene in rainbow trout a concentrated sequencing effort of the sex chromosome is needed.

## Conclusion

Here we found evidence that a proportion of the transcriptome is differentially expressed between the sexes at early stages of development in rainbow trout, prior to the morphological differentiation of the gonads. Most of the features were not connected with obvious sex function suggesting that there is sexual dimorphism in gene expression even prior to sex differentiation, and the processes that are differentially regulated are varied and complex. The early age of our sampling precluded us from looking at the transcriptome of the gonads; however the fact that so many genes are differentially expressed so early in development is a novel and interesting find. In addition, we found evidence that several candidate sex determining genes showed a significant difference in expression between males and females. In all cases, this difference was seen at one or at most two time points only, suggesting that the transcriptome of *O. mykiss *embryos goes through rapid changes early in development. It is possible that our sampling scheme has missed the key window of sexual differentiation in *O. mykiss*, to that end a more thorough sampling effort to include embryos after 33 dpf and before (or just overlapping) with 55 dpf as well as earlier points in development could further our understanding of embryonic development in *O. mykiss*.

## Methods

### Backcross lines and embryos sampled

Details of the experimental backcrosses and the conditions for raising the embryos are presented elsewhere [30]. Briefly, samples originated from a fourth generation backcross between two clonal lines of rainbow trout, whereby alleles from a fast-developing line were introgressed by repeated backcrossing into a slow-developing line. One line (Clearwater, CW, YY male) possessed alleles for fast development, and the other line (Oregon State University, OSU, XX female) possessed alleles for slower development at a major QTL for embryonic development rate. Embryos from the fourth generation backcross were reared at 11°C and sampled at 8, 15, 19, 24, 28 and 33 days post fertilization (dpf). At each time point a total of 100 embryos were collected and immediately flash frozen in liquid nitrogen and stored at -80°C.

### RNA extraction and cDNA synthesis

Total RNA was extracted from each embryo using TriZol reagent (Sigma) following the manufacturer's protocol, and RNA was further purified using RNeasy columns (Qiagen) to remove residual yolk proteins, as described [30].

### Microarray processing and data analysis

Full details for the microarray experimental design and hybridization, are presented elsewhere [30]. Briefly, four individuals of each of two QTL genotypes (OSU/OSU and OSU/CW), each of three time points (15, 19 and 28 dpf) and each sex were selected at random for the microarray experiment, for a total of 48 samples. QTL genotype was determined using three microsatellite markers linked to the major QTL for development rate [30]. Sex was determined by genotyping samples for the Y chromosome specific marker *OmyY1 *which is 96% concordant with phenotypic sex in these clonal lines [50]. The three development time points used in the microarray study were: 15 (eyed stage, 165 degree days), 19 (caudal flexing stage, 209 degree days) and 28 (beginning of hatch stage, 308 degree days) days post-fertilization (dpf). The microarray used was a 16,006 feature (16 k) array developed from ESTs of several salmonid species, primarily Atlantic salmon (*Salmo salar*) and rainbow trout, by the Consortium for Genomic Resources for All Salmonids Project [cGRASP, [51]].

As described by Xu et al. [30], after removal of features that were not detected (the signal was less than 90th percentile of the negative controls) and normalizing, the log intensity of each feature (y_ijkl_) on the microarray was tested using a mixed effects linear model y_ijkl _= μ + D_i _+ T_j _+ A_k _+e_ijkl_, where μ is the mean log intensity for each probe, D is the dye (Cy3 or Cy5), T is treatment effect and A is the array effect. The treatment effect is the combination of sex (male/female) genotype (OSU/CW, or OSU/OSU), and days post fertilization (15, 19 or 28 dpf). Dye and treatment were fixed effects while array was fitted as a random effect. An FDR of 0.05 was applied to identify features that were significant in the overall statistical model. A false discovery rate (FDR) of 0.2 was then applied across all possible pairwise treatment contrasts (n = 66) to control for type I error [52,53], as described [30]. Only those six contrasts comparing males and females at each time point and each QTL genotype were evaluated for differences between the sexes (i.e. OSU/CW males vs. OSU/CW females and OSU/OSU males vs. OSU/OSU females at each time point). In other words, all pairwise comparisons between the sexes were only made within the same genotype and at the same developmental time point. Models were evaluated using SAS JMP Genomics (SAS Institute, Cary NC, USA).

ESTs from the microarray were annotated using BLASTX, as described [30] and Blast2GO [54,55]. Blast2GO was used to both obtain gene ontology terms and to conduct a GO enrichment analysis. A Fisher's exact test with multiple testing correction (FDR of 0.05) was used for the enrichment analysis with two objectives: 1) to assess overrepresentation of GO terms in all sex-biased genes (male or female biased) relative to all features on the microarray, and 2) to assess overrepresentation of GO terms in either male or female sex-biased genes relative to the total set of sex-biased genes. All genes that were significantly differentially expressed between the sexes at each time point for each genotype were compared so that a total of six Fisher's exact tests were calculated (e.g. OSU/CW males vs. OSU/CW females and OSU/OSU males vs. OSU/OSU females at each time point). Ward hierarchical clustering was used to group all the sex-biased genes based on shared patterns of expression between all six treatments. Clustering was done with JMP Genomics (SAS, Cary, NC). We also calculated Fisher's exact tests with an applied FDR of 0.05 to examine over expression of GO terms within each of the clusters generated from shared patterns of expression.

A total of 20 genes were selected for qRT-PCR analysis from the microarray, of these 9 had GO terms suggesting functions important in sex. qRT-PCR follow up on these 20 genes identified from the microarray served to validate the microarray analysis, while also examining an increased sampling of gene expression differences in those genes associated with sex determination. All qRT-PCR experiments were conducted on a StepOnePlus (Applied Biosystems). Primers were designed from the rainbow trout EST that produced the best BLAST hit (see Additional File 6). Primers were designed (where possible) to span intron/exon boundaries so that amplification of genomic DNA in qRT-PCR could be avoided. In addition, primers were designed to have optimum binding at 58°C, so that minimum effort was required in primer optimizing. All primer design was done in Primer 3 [56].

For qRT-PCR, a total of 24 samples were run per gene (8 individuals per development time point, with equal numbers of males and females and OSU/CW and OSU/OSU genotypes). Samples were run in triplicate on the same plate with a negative control that lacked cDNA. Positive controls were set up for each sample in triplicate using beta-actin. Beta-actin was used as a reference gene because the microarray showed no differential expression among treatments (sex, genotype, and dpf) [30]. qRT-PCR mixtures consisted of 1× SYBR Green, 0.36 μM of each primer, 1 μl of template cDNA and water to 10 μl. The thermal profile for all genes was as follows; 95°C for 10 min, followed by 40 cycles of 95°C for 15 s and 58°C for 1 min. A melting curve analysis was conducted from 50°C to 90°C with 0.5°C increases per cycle for a total of 80 cycles to insure there was no mis-annealing or contaminated cDNA in the sample. For each gene PCR efficiency was tested by pooling samples of all cDNA for each developmental time period. Eight serial dilutions of the cDNA pool were used in the real-time PCR reactions on both target and reference genes to assess PCR efficiency. The qRT-PCR data was analyzed using Pfaffl's method, which corrects cycle thresholds (C_T_) for differing amplification efficiencies of the target and reference genes, this procedure is more commonly known as the ΔΔC_T _method [57].

Expression of additional candidate sex determination genes were examined, as a number of genes involved in the sex determination cascade are not represented on the microarray. A total of 16 genes known to be involved in the sex determining cascade in rainbow trout [28,29] were chosen for examination of differential expression prior to hatch in this study. The qRT-PCR was carried out as described above. The same 24 samples used for the microarray genes of interest (from 15, 19, and 28 dpf) were used for this candidate gene approach, but samples from additional time points were also analyzed for a subset of genes (*wt1*, *foxl2b*, *foxl2a*, *DMRT1*, *sox9b1*, *ovol1*, *zonadhesin *and *cyp19a1a)*. Twenty four samples from three additional developmental time points (8 each from 8, 24 and 33 dpf) were included to expand the time series examined. The additional samples consisted of 4 male and 4 female (2 OSU/OSU and 2 OSU/CW) individuals. For primer sequences and PCR amplification conditions see [28].

### Statistical methods for qRT-PCR

To test the significance of differences in expression between the sexes while accounting for time and genotype effects, general linear models were constructed. Gene expression, y was modeled as a function of sex (S), time (T) and genotype (G) as follows: y_ijkl _= S_i _+ T_j _+ G_k _+ (S_i_T_j_) + (S_i_G_k_) + (T_j_G_k_) + e_ijkl _where S is the sex of the individual (male or female), T is the developmental time point (dpf) and G is the QTL genotype of the individual (OSU/OSU or OSU/CW). All models were constructed in SAS v9.2, and a type I error rate of 0.05 was chosen to determine statistical significance of the overall model, and pairwise comparisons of treatments.

### Candidate sex gene mapping

A subset of genes that were differentially expressed between the sexes were chosen for linkage mapping, in order to identify genes that map to the putative sex determining region on the sex chromosome. The majority of the candidate sex determining genes had primers that amplified a short (150 bp or less) fragment of cDNA for qRT-PCR. Primers were redesigned in these genes to increase sequence length and increase the chances of locating SNPs. One primer pair was designed for each gene (see Additional File 7). Each gene was amplified using 1× buffer, 1 μM dNTPs, 1 μM each forward and reverse primer, 0.1 U Taq polymerase and 20-50 ng of template DNA. The thermal profile for PCR followed the same conditions as described above but with variable annealing temperatures (see Additional File 7). Once optimized, PCRs were cleaned of excess primers and unincorporated dNTPs using 1.5 U Antarctic Alkaline Phosphatase (New England BioLabs) and 0.5 U of Exonuclease I (New England BioLabs) and incubated at 37°C for 1 h, followed by heat inactivation at 75°C for 15 min.

Cleaned PCR products were cycle-sequenced using ABI BigDye 3.1 (Applied Biosystems) and size separated on an ABI 3130xl. Sequences were aligned in LaserGene (DNAstar) and investigated for the presence of SNPs that segregate between the CW and OSU clonal lines. Candidate SNPs for mapping were chosen based on the quality of the sequence, the presence of two discrete alleles, and the presence of at least 40 bp next to the SNP in both directions to allow the design of SNP specific primers. One SNP primer for each gene was designed immediately adjacent to the SNP (either the 5' or 3' end). Each SNP was genotyped in a mapping population of 110 double-haploid progeny derived from an OSUxCW cross [32,33]. SNP genotyping was conducted using ABI SNaPshot (Applied Biosystems). SNPs that were successfully genotyped were incorporated into a previously published map [32,33]. Linkage mapping was conducted in JoinMap 4, and the resulting linkage maps were drawn in MapChart v 2.0 [58].

## Authors' contributions

MCH carried out the real time PCR, performed the analyses and wrote the manuscript. PX designed and analyzed the microarray data. JS genotyped the SNPs from the candidate sex genes and mapped them to the OSU × CW cross. PAW and GH conducted the backcrosses and collected the embryos for study. KMN designed the experiment, helped analyze the results and helped write the manuscript. All authors read, provided comments and approved the final manuscript.

## Supplementary Material

Additional file 1 **Expression ratios (F/M) of all 883 features found to be statistically significantly expressed in at least one comparison**. Expression ratios and significance (p-values) for all 883 features found to be significantly differentially expressed between the sexes across all three time points and both genotypes. Click here for file

Additional file 2 **Table showing results from GLM analysis**. Results from GLM analysis, components that are statistically significant are shown in bold type. Genes identified from the microarray are above the line. Click here for file

Additional file 3 **Differences in expression between the sexes of the four candidate sex genes identifies from the microarray**. Expression plots from four of the candidate sex determining genes identified from the microarray. Click here for file

Additional file 4 **Expression profile of candidate sex genes identified from Baron et al 2005**. female/male ratio of candidate sex genes. Significant differences between the sexes within time points is shown by *, *= 0.05, ** = 0.01 Click here for file

Additional file 5 **Linkage mapping of candidate sex genes**. linage groups with mapped candidate sex genes (in red). Maps were constructed from a double-haploid cross between two populations of *O. mykiss*, see Nichols et al (2008) for details. Click here for file

Additional file 6 **Primer details from genes identified for RT-PCR analysis from the microarray**. Primer details and PCR conditions for amplification of genes identified from the microarray Click here for file

Additional file 7 **Primer details for mapping candidate sex genes**. Marker, primers and PCR conditions used for mapping candidate sex genes, PCR annealing temperatures are given in °C. Note that primers used for zonadhesin were the same as those reported in Additional File 6. Click here for file
