# Supplementary material for: Differential gene expression in male and female rainbow trout embryos prior to the onset of gross morphological differentiation of the gonads
Source: BMC Genomics. 2011 Aug 8;12:404. doi: 10.1186/1471-2164-12-404 (PMC3166948; doi:10.1186/1471-2164-12-404)
Supplement: Additional file 1 — Expression ratios (F/M) of all 883 features found to be statistically significantly expressed in at least one comparison. Expression ratios and significance (p-values) for all 883 features found to be significantly differentially expressed between the sexes across all three time points and both genotypes. [file 1471-2164-12-404-S1.DOCX]

Additional File 1. Expression ratios (F/M) of all 883 features found to be statistically significantly expressed in at least one comparison.

| ID | Blast hit | CW 15 | P value | Cw 19 | P value | Cw 28 | P value | OSU 15 | P value | OSU 19 | P value | OSU 28 | P value |
| --- | --- | --- | --- | --- | --- | --- | --- | --- | --- | --- | --- | --- | --- |
| CA769480 | Endothelial differentiation-related factor 1 homolog | 1.01 | 0.94 | 1.23 | 0.17 | 0.78 | 0.10 | 0.83 | 0.27 | 1.75 | 0.00 | 1.00 | 0.98 |
| CB493673 | UNKNOWN | 0.48 | 0.00 | 0.70 | 0.10 | 1.01 | 0.95 | 0.67 | 0.11 | 1.05 | 0.82 | 1.20 | 0.36 |
| CB511393 | UNKNOWN | 1.80 | 0.10 | 0.83 | 0.59 | 0.91 | 0.78 | 2.60 | 0.01 | 1.09 | 0.80 | 1.65 | 0.14 |
| CK991100 | Prolactin regulatory element-binding protein | 0.64 | 0.19 | 1.61 | 0.14 | 0.40 | 0.01 | 0.75 | 0.38 | 1.42 | 0.27 | 0.61 | 0.13 |
| CA044607 | UNKNOWN | 6.81 | 0.00 | 0.77 | 0.65 | 1.87 | 0.28 | 0.72 | 0.57 | 1.95 | 0.28 | 1.92 | 0.26 |
| CA059433 | H+/organic cation antiporter variant 2 [Rattus norvegicus] | 1.34 | 0.20 | 0.92 | 0.70 | 1.00 | 0.98 | 1.13 | 0.59 | 2.12 | 0.00 | 0.91 | 0.66 |
| CA061134 | UNKNOWN | 2.43 | 0.00 | 0.90 | 0.66 | 1.00 | 0.98 | 1.34 | 0.29 | 1.22 | 0.38 | 1.29 | 0.30 |
| CB493415 | Salmo salar partial mRNA for myosin regulatory light chain | 0.45 | 0.00 | 0.67 | 0.13 | 2.26 | 0.02 | 0.93 | 0.76 | 1.34 | 0.28 | 0.99 | 0.98 |
| CB517155 | Protein-glutamine gamma-glutamyltransferase 2 | 0.74 | 0.26 | 1.08 | 0.78 | 0.43 | 0.00 | 0.83 | 0.47 | 1.49 | 0.16 | 0.73 | 0.25 |
| CB496550 | COP9 signalosome complex subunit 5 | 1.50 | 0.23 | 0.47 | 0.01 | 0.73 | 0.27 | 2.35 | 0.02 | 0.61 | 0.08 | 1.35 | 0.29 |
| CK990594 | Salmo salar Tc1 transposon, tn6 gene | 0.40 | 0.00 | 0.71 | 0.20 | 0.74 | 0.29 | 0.64 | 0.14 | 1.05 | 0.84 | 1.03 | 0.91 |
| CB498218 | Creatine kinase M-type | 0.43 | 0.01 | 0.69 | 0.19 | 1.59 | 0.15 | 0.94 | 0.84 | 1.64 | 0.09 | 1.13 | 0.70 |
| CA040273 | UNKNOWN | 0.74 | 0.12 | 0.60 | 0.01 | 0.82 | 0.34 | 1.11 | 0.59 | 1.07 | 0.72 | 0.95 | 0.82 |
| CB498377 | Homeobox protein Nkx-2.5 | 1.11 | 0.58 | 0.93 | 0.73 | 0.78 | 0.24 | 0.53 | 0.00 | 0.72 | 0.12 | 1.07 | 0.75 |
| CB500124 | UNKNOWN | 1.23 | 0.64 | 0.43 | 0.10 | 1.22 | 0.64 | 0.96 | 0.93 | 0.25 | 0.01 | 1.00 | 0.99 |
| CA052422 | Ras-related C3 botulinum toxin substrate 2 precursor | 1.78 | 0.15 | 0.46 | 0.05 | 0.90 | 0.79 | 1.19 | 0.67 | 0.35 | 0.01 | 1.23 | 0.61 |
| CA059819 | RNA-binding protein 12 | 2.05 | 0.07 | 0.39 | 0.01 | 1.24 | 0.69 | 1.72 | 0.15 | 0.53 | 0.07 | 1.34 | 0.59 |
| CB497763 | Complement C3-1 | 1.00 | 0.98 | 0.62 | 0.03 | 0.93 | 0.72 | 0.57 | 0.01 | 0.86 | 0.48 | 0.74 | 0.14 |
| CA043326 | Heterogeneous nuclear ribonucleoprotein K | 0.68 | 0.12 | 0.47 | 0.00 | 0.91 | 0.70 | 0.87 | 0.56 | 0.56 | 0.02 | 0.88 | 0.61 |
| CB509827 | Fatty acid-binding protein, heart | 1.08 | 0.57 | 0.67 | 0.01 | 0.93 | 0.64 | 1.19 | 0.24 | 0.82 | 0.17 | 0.97 | 0.84 |
| CA059063 | UNKNOWN | 0.89 | 0.61 | 0.94 | 0.77 | 1.97 | 0.03 | 0.51 | 0.01 | 0.84 | 0.44 | 0.96 | 0.89 |
| CB503530 | Fos-related antigen 2 | 0.32 | 0.01 | 0.61 | 0.27 | 1.96 | 0.13 | 0.61 | 0.30 | 0.50 | 0.12 | 2.08 | 0.10 |
| CK990398 | Oncorhynchus mykiss SYPG1 (SYPG1), PHF1 (PHF1) | 1.20 | 0.51 | 0.60 | 0.09 | 1.23 | 0.48 | 0.72 | 0.23 | 0.45 | 0.01 | 1.74 | 0.07 |
| CA051935 | Isoleucyl-tRNA synthetase, mitochondrial precursor | 0.74 | 0.15 | 0.78 | 0.24 | 1.07 | 0.75 | 1.02 | 0.92 | 0.56 | 0.01 | 1.05 | 0.79 |
| CA049297 | CCR4-NOT transcription complex subunit 8 | 0.36 | 0.00 | 0.61 | 0.13 | 1.21 | 0.51 | 0.62 | 0.11 | 0.67 | 0.20 | 1.17 | 0.59 |
| CA054369 | UNKNOWN | 0.89 | 0.65 | 0.69 | 0.17 | 0.75 | 0.30 | 0.54 | 0.02 | 0.45 | 0.00 | 1.29 | 0.35 |
| CB507061 | Guanine nucleotide-binding protein subunit beta 1 | 1.07 | 0.77 | 0.59 | 0.02 | 1.29 | 0.37 | 0.90 | 0.67 | 0.72 | 0.13 | 2.44 | 0.00 |
| CA039346 | Ornithine aminotransferase, mitochondrial precursor | 6.05 | 0.00 | 0.57 | 0.08 | 1.11 | 0.74 | 1.57 | 0.22 | 0.83 | 0.55 | 2.49 | 0.01 |
| CB496795 | Serine/threonine-protein kinase Sgk3 | 0.76 | 0.50 | 0.79 | 0.54 | 2.33 | 0.03 | 0.73 | 0.43 | 0.55 | 0.12 | 2.91 | 0.01 |
| CK990247 | Dihydrolipoyl dehydrogenase, mitochondrial precursor | 0.63 | 0.27 | 0.34 | 0.01 | 1.29 | 0.54 | 0.45 | 0.06 | 0.85 | 0.70 | 1.44 | 0.38 |
| CB503485 | BTB/POZ domain-containing protein KCTD5 | 0.35 | 0.07 | 0.98 | 0.97 | 1.16 | 0.79 | 0.21 | 0.01 | 1.03 | 0.96 | 1.52 | 0.45 |
| CA044566 | Staphylococcal nuclease domain-containing protein 1 | 1.07 | 0.64 | 1.07 | 0.62 | 0.68 | 0.01 | 1.12 | 0.44 | 1.07 | 0.62 | 0.99 | 0.96 |
| CA052061 | Mixed lineage kinase domain-like protein | 0.80 | 0.18 | 1.02 | 0.88 | 0.85 | 0.28 | 0.63 | 0.01 | 1.14 | 0.35 | 1.14 | 0.38 |
| CA054140 | PREDICTED: similar to Homeodomain leucine zipper gene | 0.93 | 0.83 | 0.44 | 0.01 | 1.00 | 1.00 | 1.24 | 0.50 | 0.57 | 0.07 | 1.17 | 0.62 |
| CA059937 | UNKNOWN | 1.31 | 0.31 | 0.84 | 0.54 | 0.79 | 0.36 | 1.38 | 0.23 | 0.43 | 0.01 | 1.06 | 0.81 |
| CA041264 | UNKNOWN | 1.08 | 0.77 | 0.54 | 0.01 | 1.08 | 0.76 | 1.36 | 0.23 | 0.86 | 0.55 | 1.16 | 0.56 |
| CB511701 | Protein YIPF6 | 0.91 | 0.74 | 1.05 | 0.87 | 1.33 | 0.31 | 0.47 | 0.01 | 1.06 | 0.83 | 1.03 | 0.92 |
| CK990598 | Elastase-1 | 1.33 | 0.41 | 0.52 | 0.06 | 1.70 | 0.13 | 0.71 | 0.32 | 0.36 | 0.00 | 1.46 | 0.28 |
| CA045552 | UNKNOWN | 0.93 | 0.90 | 0.52 | 0.25 | 1.36 | 0.61 | 0.18 | 0.01 | 0.68 | 0.49 | 1.02 | 0.97 |
| CA044998 | Uncharacterized protein C4orf32 | 1.83 | 0.10 | 0.39 | 0.01 | 1.33 | 0.42 | 1.12 | 0.76 | 0.48 | 0.05 | 1.34 | 0.42 |
| CB502715 | Pancreatic secretory granule membrane glycoprotein | 0.98 | 0.96 | 0.31 | 0.01 | 1.88 | 0.12 | 1.09 | 0.82 | 0.23 | 0.00 | 1.23 | 0.61 |
| CA058391 | UNKNOWN | 1.01 | 0.98 | 0.90 | 0.79 | 1.70 | 0.21 | 0.30 | 0.01 | 0.73 | 0.45 | 2.06 | 0.09 |
| CB517268 | UNKNOWN | 0.14 | 0.01 | 0.47 | 0.21 | 0.79 | 0.69 | 0.15 | 0.01 | 0.69 | 0.54 | 1.16 | 0.80 |
| CA057082 | Circumsporozoite protein precursor | 0.27 | 0.01 | 0.71 | 0.45 | 0.88 | 0.78 | 0.62 | 0.32 | 0.94 | 0.89 | 0.94 | 0.89 |
| CK990989 | Elongation factor 1-alpha, oocyte form | 0.19 | 0.01 | 0.79 | 0.68 | 0.74 | 0.55 | 0.24 | 0.02 | 1.01 | 0.98 | 0.91 | 0.85 |
| CA057022 | Slc9a3r2 protein [Danio rerio] | 0.67 | 0.14 | 0.95 | 0.84 | 0.98 | 0.93 | 0.50 | 0.01 | 1.11 | 0.69 | 0.87 | 0.61 |
| CB515883 | Sodium/potassium-transporting ATPase subunit beta-233 | 0.53 | 0.12 | 0.67 | 0.30 | 1.14 | 0.73 | 0.21 | 0.00 | 0.69 | 0.34 | 1.44 | 0.35 |
| CB498116 | Troponin T, fast skeletal muscle | 0.42 | 0.00 | 0.82 | 0.48 | 1.31 | 0.31 | 0.61 | 0.08 | 1.62 | 0.10 | 1.11 | 0.70 |
| CB508291 | Oncorhynchus mykiss SYPG1 (SYPG1), PHF1 (PHF1 | 0.48 | 0.15 | 0.25 | 0.01 | 0.76 | 0.58 | 0.55 | 0.24 | 0.50 | 0.20 | 0.84 | 0.73 |
| CA055129 | UNKNOWN | 0.77 | 0.42 | 0.40 | 0.01 | 1.32 | 0.35 | 1.23 | 0.50 | 0.62 | 0.15 | 1.43 | 0.24 |
| CB512268 | Sodium/potassium/calcium exchanger 6 precursor | 0.48 | 0.05 | 0.69 | 0.34 | 0.99 | 0.99 | 0.37 | 0.01 | 0.56 | 0.14 | 0.84 | 0.62 |
| CA051319 | Mus musculus PAX interacting | 0.48 | 0.25 | 0.18 | 0.01 | 0.68 | 0.53 | 0.53 | 0.33 | 0.37 | 0.14 | 0.74 | 0.62 |
| CA056048 | UNKNOWN | 0.83 | 0.74 | 0.18 | 0.01 | 0.92 | 0.87 | 1.05 | 0.94 | 0.49 | 0.29 | 1.17 | 0.76 |
| CB499556 | GTP-binding protein era homolog | 0.60 | 0.24 | 0.67 | 0.33 | 1.92 | 0.15 | 0.28 | 0.01 | 0.73 | 0.45 | 2.57 | 0.04 |
| CA038261 | Oncorhynchus mykiss carbonyl reductase | 0.68 | 0.28 | 0.68 | 0.27 | 0.83 | 0.60 | 0.31 | 0.00 | 0.73 | 0.37 | 1.24 | 0.53 |
| CA054545 | UNKNOWN | 0.64 | 0.17 | 1.35 | 0.33 | 0.34 | 0.00 | 0.73 | 0.32 | 1.15 | 0.64 | 0.63 | 0.17 |
| CA038949 | UNKNOWN | 0.52 | 0.04 | 0.38 | 0.00 | 1.14 | 0.67 | 0.75 | 0.36 | 0.54 | 0.05 | 1.41 | 0.26 |
| CB511353 | Cytochrome c oxidase polypeptide | 1.31 | 0.65 | 0.24 | 0.01 | 0.52 | 0.28 | 0.47 | 0.18 | 0.36 | 0.07 | 1.60 | 0.39 |
| CA061989 | 40S ribosomal protein S27a | 0.85 | 0.62 | 0.34 | 0.00 | 0.86 | 0.65 | 1.01 | 0.97 | 0.52 | 0.06 | 1.40 | 0.32 |
| CA057270 | Splicing factor, arginine/serine-rich 2 | 0.48 | 0.02 | 0.59 | 0.10 | 1.11 | 0.75 | 0.39 | 0.00 | 0.95 | 0.87 | 1.66 | 0.14 |
| CB510302 | UNKNOWN | 1.07 | 0.91 | 0.43 | 0.09 | 1.65 | 0.34 | 0.22 | 0.00 | 0.65 | 0.38 | 1.06 | 0.91 |
| CB508919 | Ubiquitin-conjugating enzyme E2 D4 | 0.62 | 0.19 | 0.53 | 0.08 | 1.60 | 0.22 | 0.29 | 0.00 | 0.91 | 0.78 | 2.09 | 0.06 |
| CK990427 | UNKNOWN | 0.28 | 0.01 | 0.74 | 0.47 | 2.21 | 0.05 | 0.55 | 0.15 | 0.95 | 0.90 | 2.41 | 0.03 |
| CB517606 | 2-oxoisovalerate dehydrogenase subunit beta | 0.58 | 0.05 | 1.09 | 0.76 | 0.97 | 0.89 | 0.35 | 0.00 | 1.14 | 0.65 | 1.35 | 0.24 |
| CA063442 | UNKNOWN | 0.46 | 0.01 | 0.49 | 0.02 | 0.85 | 0.56 | 0.73 | 0.28 | 0.69 | 0.18 | 1.14 | 0.63 |
| CA055258 | Eukaryotic translation initiation factor 6 | 0.29 | 0.00 | 0.93 | 0.86 | 1.28 | 0.56 | 0.39 | 0.03 | 0.86 | 0.73 | 1.45 | 0.38 |
| CA059604 | Signal peptidase complex subunit 3 | 0.27 | 0.00 | 0.79 | 0.44 | 0.84 | 0.58 | 0.50 | 0.04 | 1.15 | 0.65 | 1.31 | 0.39 |
| CB505346 | Mon2 protein [Mus musculus] | 0.59 | 0.07 | 0.93 | 0.80 | 0.81 | 0.48 | 0.41 | 0.00 | 1.23 | 0.49 | 1.19 | 0.56 |
| CA038063 | unknown protein [Siniperca chuatsi] | 1.14 | 0.63 | 0.57 | 0.06 | 0.88 | 0.66 | 0.49 | 0.01 | 0.68 | 0.18 | 1.18 | 0.56 |
| CA037272 | UNKNOWN | 0.94 | 0.88 | 0.59 | 0.22 | 0.95 | 0.92 | 0.66 | 0.38 | 0.32 | 0.01 | 1.61 | 0.31 |
| CB507745 | UNKNOWN | 1.51 | 0.31 | 0.35 | 0.01 | 1.02 | 0.96 | 1.62 | 0.21 | 0.59 | 0.15 | 1.27 | 0.51 |
| CB515608 | PREDICTED: similar to LOC531467 protein | 0.89 | 0.74 | 0.63 | 0.14 | 1.97 | 0.05 | 1.06 | 0.88 | 0.88 | 0.69 | 2.36 | 0.01 |
| CA051196 | 60S acidic ribosomal protein P2 | 0.38 | 0.01 | 0.80 | 0.50 | 0.81 | 0.49 | 0.57 | 0.11 | 0.98 | 0.95 | 0.95 | 0.87 |
| CB509466 | O.mykiss vtg1 gene | 1.04 | 0.92 | 0.34 | 0.01 | 0.85 | 0.69 | 1.04 | 0.92 | 0.56 | 0.15 | 1.03 | 0.95 |
| CB507609 | 40S ribosomal protein S23 | 0.40 | 0.01 | 0.53 | 0.13 | 1.07 | 0.85 | 0.62 | 0.17 | 0.99 | 0.97 | 1.17 | 0.66 |
| CB515363 | Transmembrane protein 35 | 0.91 | 0.88 | 0.59 | 0.41 | 0.79 | 0.76 | 0.52 | 0.31 | 0.41 | 0.18 | 9.77 | 0.01 |
| CA056678 | Chronic lymphocytic leukemia deletion region gene 6 | 0.93 | 0.82 | 0.44 | 0.01 | 0.70 | 0.31 | 1.24 | 0.49 | 0.62 | 0.13 | 1.20 | 0.60 |
| CB513887 | UNKNOWN | 0.60 | 0.28 | 0.23 | 0.00 | 0.86 | 0.69 | 0.87 | 0.77 | 0.47 | 0.06 | 1.60 | 0.23 |
| CB517795 | VCX-C protein | 0.24 | 0.00 | 0.66 | 0.22 | 1.13 | 0.71 | 0.36 | 0.01 | 0.80 | 0.50 | 1.60 | 0.17 |
| CB517459 | Zinc finger protein 189 | 0.37 | 0.01 | 1.00 | 1.00 | 0.71 | 0.36 | 0.57 | 0.15 | 0.70 | 0.32 | 0.91 | 0.80 |
| CA042822 | Glucose-6-phosphate translocase | 0.80 | 0.50 | 0.61 | 0.19 | 1.12 | 0.74 | 0.36 | 0.00 | 0.62 | 0.21 | 1.15 | 0.66 |
| CB515643 | Ig kappa chain V-IV region JI precursor | 0.63 | 0.17 | 0.54 | 0.06 | 1.47 | 0.21 | 1.50 | 0.20 | 0.42 | 0.01 | 1.82 | 0.05 |
| CA052491 | Mps one binder kinase activator-like 2A | 0.64 | 0.15 | 0.75 | 0.36 | 0.89 | 0.68 | 0.59 | 0.09 | 0.44 | 0.01 | 1.15 | 0.64 |
| CB493611 | Regulator of G-protein signaling 16 | 0.32 | 0.01 | 0.51 | 0.08 | 1.33 | 0.45 | 0.53 | 0.14 | 0.57 | 0.13 | 1.89 | 0.09 |
| CA055115 | T-complex protein 1 subunit alpha | 1.20 | 0.63 | 0.44 | 0.03 | 1.82 | 0.16 | 0.37 | 0.01 | 0.75 | 0.46 | 1.56 | 0.29 |
| CA059968 | Keratin, type I cytoskeletal 18 | 0.35 | 0.01 | 0.78 | 0.51 | 0.67 | 0.30 | 0.62 | 0.19 | 0.79 | 0.53 | 0.71 | 0.38 |
| CA059450 | Estradiol 17-beta-dehydrogenase 12-B | 0.73 | 0.48 | 0.92 | 0.84 | 0.87 | 0.73 | 0.27 | 0.00 | 0.63 | 0.25 | 1.08 | 0.85 |
| CK991003 | UNKNOWN | 0.49 | 0.07 | 0.37 | 0.00 | 1.75 | 0.08 | 0.97 | 0.93 | 0.67 | 0.22 | 1.56 | 0.17 |
| CB517027 | Epithelial-cadherin precursor | 0.66 | 0.11 | 1.18 | 0.51 | 2.07 | 0.01 | 0.61 | 0.06 | 1.01 | 0.98 | 1.51 | 0.13 |
| CA045393 | UNKNOWN | 0.13 | 0.00 | 0.48 | 0.25 | 0.72 | 0.59 | 0.10 | 0.00 | 0.48 | 0.24 | 0.67 | 0.52 |
| CB490431 | Probable ATP-dependent RNA helicase DDX4 | 1.30 | 0.50 | 0.45 | 0.03 | 1.27 | 0.51 | 0.60 | 0.19 | 0.33 | 0.00 | 1.15 | 0.70 |
| CB496725 | Creatine kinase, sarcomeric mitochondrial precursor | 0.54 | 0.16 | 0.29 | 0.00 | 0.81 | 0.61 | 0.70 | 0.41 | 0.52 | 0.12 | 1.23 | 0.61 |
| CA064179 | Engulfment and cell motility protein 1 | 0.78 | 0.50 | 1.22 | 0.56 | 0.78 | 0.53 | 0.30 | 0.00 | 0.83 | 0.59 | 0.75 | 0.46 |
| CA061032 | UNKNOWN | 1.16 | 0.65 | 0.40 | 0.01 | 1.02 | 0.96 | 1.18 | 0.61 | 0.58 | 0.11 | 1.14 | 0.68 |
| CB494064 | 60S ribosomal protein L35 | 0.39 | 0.01 | 0.50 | 0.03 | 1.19 | 0.57 | 0.59 | 0.13 | 0.86 | 0.63 | 1.43 | 0.26 |
| CA048064 | UNKNOWN | 0.87 | 0.59 | 2.85 | 0.00 | 0.97 | 0.92 | 0.67 | 0.12 | 1.12 | 0.68 | 1.00 | 0.99 |
| CB498819 | UNKNOWN | 0.69 | 0.15 | 0.47 | 0.01 | 1.00 | 0.99 | 1.12 | 0.65 | 0.72 | 0.26 | 1.17 | 0.53 |
| CA054499 | Cell division control protein 42 homolog precursor | 0.75 | 0.33 | 1.49 | 0.21 | 0.81 | 0.50 | 0.45 | 0.01 | 1.13 | 0.69 | 1.32 | 0.38 |
| CB511680 | Lysozyme C II precursor | 0.52 | 0.04 | 0.69 | 0.25 | 1.08 | 0.82 | 0.35 | 0.00 | 0.78 | 0.45 | 1.14 | 0.69 |
| CA048888 | UNKNOWN | 0.21 | 0.02 | 0.70 | 0.55 | 1.53 | 0.47 | 0.11 | 0.01 | 0.78 | 0.70 | 1.49 | 0.50 |
| BU965792 | Myosin heavy chain, fast skeletal muscle | 0.26 | 0.01 | 0.50 | 0.14 | 0.85 | 0.76 | 0.76 | 0.59 | 1.32 | 0.55 | 1.08 | 0.88 |
| CA062276 | Pyruvate dehydrogenase E1 component subunit | 3.03 | 0.01 | 1.12 | 0.81 | 1.14 | 0.73 | 1.42 | 0.37 | 0.90 | 0.83 | 1.27 | 0.53 |
| CB502496 | PREDICTED: similar to FADS6 protein [Gallus gallus] | 1.62 | 0.26 | 0.33 | 0.01 | 1.17 | 0.71 | 0.92 | 0.85 | 0.41 | 0.04 | 1.58 | 0.27 |
| CB502142 | WD repeat protein 37 | 1.00 | 0.99 | 0.38 | 0.00 | 1.60 | 0.15 | 0.75 | 0.41 | 0.69 | 0.24 | 1.80 | 0.07 |
| CA768458 | UNKNOWN | 0.79 | 0.32 | 0.48 | 0.00 | 0.96 | 0.87 | 0.89 | 0.62 | 0.70 | 0.11 | 1.31 | 0.30 |
| CA041000 | Mps one binder kinase activator-like 1B | 0.56 | 0.13 | 0.63 | 0.17 | 0.75 | 0.35 | 0.34 | 0.01 | 0.54 | 0.07 | 1.03 | 0.92 |
| CA038646 | Periostin precursor | 0.79 | 0.48 | 4.02 | 0.00 | 0.79 | 0.47 | 0.50 | 0.04 | 1.52 | 0.30 | 0.98 | 0.95 |
| CA053841 | UNKNOWN | 0.98 | 0.96 | 0.41 | 0.01 | 0.85 | 0.67 | 0.93 | 0.81 | 0.62 | 0.13 | 1.36 | 0.40 |
| CA060895 | 60S ribosomal protein L6 | 0.61 | 0.11 | 0.41 | 0.01 | 1.01 | 0.98 | 0.78 | 0.42 | 0.91 | 0.78 | 1.33 | 0.32 |
| CA057752 | UNKNOWN | 0.42 | 0.01 | 0.68 | 0.16 | 0.92 | 0.76 | 0.48 | 0.02 | 1.28 | 0.36 | 0.94 | 0.82 |
| CK990945 | 39S ribosomal protein L46, mitochondrial precursor | 0.96 | 0.87 | 1.96 | 0.01 | 0.94 | 0.81 | 1.04 | 0.87 | 1.43 | 0.18 | 1.19 | 0.48 |
| CB506018 | Beta-galactoside-binding lectin | 3.35 | 0.00 | 0.69 | 0.30 | 1.15 | 0.70 | 1.88 | 0.12 | 0.95 | 0.88 | 1.41 | 0.33 |
| CK990220 | Fatty acid-binding protein, adipocyte | 1.69 | 0.01 | 1.10 | 0.61 | 0.96 | 0.82 | 0.86 | 0.43 | 0.90 | 0.55 | 0.83 | 0.32 |
| CA064271 | Transcriptional repressor protein YY1 | 0.97 | 0.90 | 2.47 | 0.01 | 1.04 | 0.90 | 1.06 | 0.81 | 2.18 | 0.02 | 1.10 | 0.72 |
| CK990270 | Protein BTG1 | 1.50 | 0.17 | 0.99 | 0.98 | 1.46 | 0.19 | 0.85 | 0.59 | 0.88 | 0.69 | 2.29 | 0.01 |
| CB496636 | Dehydrogenase/reductase SDR family member 7 | 2.57 | 0.02 | 1.03 | 0.95 | 1.10 | 0.82 | 2.81 | 0.01 | 0.86 | 0.70 | 1.57 | 0.25 |
| CA050613 | Lysosomal-associated transmembrane protein 4A | 1.99 | 0.00 | 1.07 | 0.68 | 0.98 | 0.92 | 1.50 | 0.04 | 0.93 | 0.69 | 0.81 | 0.28 |
| CB508432 | UNKNOWN | 1.24 | 0.23 | 0.64 | 0.02 | 0.94 | 0.74 | 1.26 | 0.21 | 0.62 | 0.01 | 1.49 | 0.04 |
| CA052263 | UNKNOWN | 1.37 | 0.21 | 1.00 | 1.00 | 0.52 | 0.01 | 1.75 | 0.03 | 1.01 | 0.95 | 0.76 | 0.22 |
| CB510930 | UNKNOWN | 1.61 | 0.14 | 0.38 | 0.00 | 0.93 | 0.82 | 1.31 | 0.39 | 0.65 | 0.18 | 0.89 | 0.70 |
| CB511080 | Mitochondrial carrier homolog 2 | 1.46 | 0.04 | 0.66 | 0.01 | 0.96 | 0.79 | 1.19 | 0.32 | 0.78 | 0.12 | 1.14 | 0.42 |
| CA056122 | UNKNOWN | 0.86 | 0.57 | 1.12 | 0.62 | 1.15 | 0.64 | 0.66 | 0.14 | 0.86 | 0.51 | 2.17 | 0.01 |
| CK990671 | Oncorhynchus mykiss SYPG1 (SYPG1), PHF1 (PHF1) | 2.44 | 0.11 | 0.26 | 0.01 | 0.92 | 0.88 | 1.16 | 0.78 | 0.51 | 0.21 | 1.07 | 0.91 |
| CB500867 | Dihydrolipoyl dehydrogenase, mitochondrial precursor | 1.07 | 0.75 | 0.51 | 0.01 | 1.01 | 0.96 | 1.13 | 0.58 | 0.81 | 0.36 | 1.55 | 0.04 |
| CB510978 | Tropomyosin-1 alpha chain | 0.43 | 0.01 | 0.58 | 0.10 | 1.03 | 0.91 | 0.71 | 0.24 | 1.26 | 0.47 | 1.17 | 0.54 |
| CB500706 | Rho GTPase-activating protein 11A precursor | 1.56 | 0.05 | 0.65 | 0.07 | 0.85 | 0.40 | 0.87 | 0.50 | 0.72 | 0.15 | 1.82 | 0.00 |
| CB496449 | Peroxiredoxin-5, mitochondrial precursor | 1.99 | 0.01 | 0.85 | 0.45 | 1.13 | 0.58 | 1.77 | 0.02 | 1.10 | 0.67 | 1.15 | 0.54 |
| CA061329 | UNKNOWN | 1.32 | 0.40 | 0.40 | 0.01 | 0.66 | 0.25 | 0.90 | 0.75 | 0.71 | 0.32 | 0.83 | 0.60 |
| CB498121 | Mitochondrial 28S ribosomal protein S21 | 2.35 | 0.01 | 0.45 | 0.01 | 1.24 | 0.45 | 2.50 | 0.00 | 0.62 | 0.11 | 1.15 | 0.61 |
| CB500896 | UNKNOWN | 1.65 | 0.06 | 0.56 | 0.01 | 1.12 | 0.61 | 0.88 | 0.62 | 0.67 | 0.08 | 2.11 | 0.00 |
| CB497962 | Septin-10 | 1.01 | 0.97 | 0.37 | 0.01 | 0.94 | 0.87 | 1.25 | 0.53 | 0.99 | 0.99 | 1.43 | 0.34 |
| CA058540 | UNKNOWN | 1.45 | 0.03 | 0.97 | 0.84 | 0.98 | 0.89 | 1.54 | 0.01 | 0.82 | 0.26 | 0.78 | 0.15 |
| CK990741 | 60S acidic ribosomal protein P1 | 1.56 | 0.13 | 0.46 | 0.01 | 1.05 | 0.87 | 1.66 | 0.08 | 0.38 | 0.00 | 0.90 | 0.71 |
| CB510090 | Cofilin-2 | 0.30 | 0.01 | 0.79 | 0.54 | 2.79 | 0.02 | 0.38 | 0.02 | 1.50 | 0.29 | 2.24 | 0.06 |
| CB502180 | UNKNOWN | 1.16 | 0.62 | 0.48 | 0.01 | 1.00 | 0.99 | 1.05 | 0.88 | 0.98 | 0.95 | 1.27 | 0.36 |
| CA041569 | 39S ribosomal protein L46, mitochondrial precursor | 0.76 | 0.36 | 0.38 | 0.00 | 1.16 | 0.62 | 0.88 | 0.67 | 0.53 | 0.06 | 1.74 | 0.07 |
| CA044816 | Macaca fascicularis brain cDNA, clone:QnpA-21739 | 0.99 | 0.96 | 1.41 | 0.20 | 1.28 | 0.36 | 0.78 | 0.36 | 1.31 | 0.31 | 2.11 | 0.01 |
| CB487825 | UNKNOWN | 0.48 | 0.04 | 1.03 | 0.94 | 1.86 | 0.07 | 0.56 | 0.11 | 1.08 | 0.81 | 2.37 | 0.01 |
| CA044537 | UNKNOWN | 0.91 | 0.70 | 0.55 | 0.01 | 0.62 | 0.06 | 0.97 | 0.91 | 0.88 | 0.58 | 1.12 | 0.64 |
| CA044880 | Glutathione S-transferase theta-1 | 0.38 | 0.01 | 0.76 | 0.41 | 1.48 | 0.24 | 0.51 | 0.05 | 0.77 | 0.43 | 1.72 | 0.11 |
| CB512328 | PREDICTED: KIAA0674 [Pan troglodytes] | 0.71 | 0.33 | 0.49 | 0.04 | 1.57 | 0.22 | 1.02 | 0.95 | 0.62 | 0.16 | 2.59 | 0.01 |
| CB505804 | UNKNOWN | 1.39 | 0.47 | 0.25 | 0.00 | 1.69 | 0.28 | 1.04 | 0.94 | 0.42 | 0.06 | 2.24 | 0.10 |
| CA050892 | UNKNOWN | 0.58 | 0.01 | 1.30 | 0.19 | 0.84 | 0.38 | 0.67 | 0.06 | 1.22 | 0.31 | 1.63 | 0.02 |
| CK990948 | NADH dehydrogenase [ubiquinone] 1 beta | 0.71 | 0.39 | 0.65 | 0.27 | 1.22 | 0.62 | 1.24 | 0.60 | 0.35 | 0.01 | 1.11 | 0.80 |
| CA059885 | Solute carrier family 22 member 20 | 0.74 | 0.26 | 2.28 | 0.01 | 0.76 | 0.36 | 0.84 | 0.52 | 0.81 | 0.46 | 0.92 | 0.77 |
| CA058395 | UNKNOWN | 0.81 | 0.53 | 0.50 | 0.01 | 0.80 | 0.38 | 0.93 | 0.84 | 0.76 | 0.29 | 1.08 | 0.75 |
| CB496364 | Protein FAM46C | 0.67 | 0.11 | 1.90 | 0.01 | 1.04 | 0.87 | 0.63 | 0.06 | 1.01 | 0.97 | 0.88 | 0.58 |
| CK990969 | UNKNOWN | 0.90 | 0.63 | 0.80 | 0.32 | 1.04 | 0.85 | 0.92 | 0.71 | 0.92 | 0.69 | 1.78 | 0.01 |
| CA046635 | UNKNOWN | 0.99 | 0.95 | 1.02 | 0.91 | 1.04 | 0.87 | 1.03 | 0.89 | 0.87 | 0.49 | 2.01 | 0.01 |
| CA044989 | Plastin-1 | 1.36 | 0.41 | 0.19 | 0.00 | 1.01 | 0.97 | 1.49 | 0.29 | 0.48 | 0.17 | 1.36 | 0.44 |
| CA060416 | UNKNOWN | 0.90 | 0.53 | 1.34 | 0.13 | 1.20 | 0.27 | 0.74 | 0.07 | 1.22 | 0.30 | 2.33 | 0.00 |
| CB501661 | UNKNOWN | 3.73 | 0.01 | 0.64 | 0.37 | 1.45 | 0.45 | 0.89 | 0.83 | 0.42 | 0.08 | 1.77 | 0.26 |
| CK991016 | 40S ribosomal protein SA | 0.25 | 0.01 | 1.34 | 0.58 | 2.36 | 0.08 | 0.27 | 0.02 | 1.99 | 0.19 | 4.33 | 0.00 |
| CB507253 | pfam00909, Ammonium_transp, Ammonium Transporter | 0.91 | 0.83 | 0.27 | 0.01 | 1.16 | 0.75 | 0.66 | 0.33 | 0.45 | 0.08 | 3.19 | 0.02 |
| CA045910 | Salmo salar zonadhesin-like gene, complete cds and 3' | 0.80 | 0.45 | 0.37 | 0.00 | 0.86 | 0.58 | 1.15 | 0.64 | 0.51 | 0.03 | 1.40 | 0.23 |
| CB515130 | Vacuolar protein sorting-associated protein 26A | 0.46 | 0.03 | 0.39 | 0.01 | 1.28 | 0.50 | 0.33 | 0.00 | 0.81 | 0.53 | 2.47 | 0.02 |
| CB509409 | Apoptosis regulatory protein Siva | 0.59 | 0.17 | 0.40 | 0.01 | 1.60 | 0.21 | 0.82 | 0.57 | 0.69 | 0.28 | 1.46 | 0.31 |
| CB515819 | UNKNOWN | 0.32 | 0.02 | 1.49 | 0.37 | 3.54 | 0.01 | 0.35 | 0.03 | 1.85 | 0.17 | 3.44 | 0.01 |
| CB514957 | Nucleolar protein 5 | 0.62 | 0.05 | 1.45 | 0.13 | 0.96 | 0.86 | 0.53 | 0.01 | 1.71 | 0.03 | 0.91 | 0.71 |
| CA043220 | Platelet glycoprotein 4 | 0.53 | 0.03 | 1.02 | 0.93 | 1.28 | 0.38 | 0.45 | 0.01 | 1.01 | 0.98 | 1.36 | 0.28 |
| CB492444 | 60S ribosomal protein L8 | 0.35 | 0.01 | 0.87 | 0.72 | 1.67 | 0.23 | 0.41 | 0.03 | 1.60 | 0.23 | 1.96 | 0.11 |
| CA061276 | Iron-sulfur cluster assembly enzyme ISCU, | 0.85 | 0.52 | 0.51 | 0.01 | 1.18 | 0.49 | 1.07 | 0.78 | 0.69 | 0.16 | 1.35 | 0.22 |
| CA064485 | Transmembrane protein 118 | 0.87 | 0.69 | 2.34 | 0.01 | 0.66 | 0.23 | 1.24 | 0.53 | 1.04 | 0.91 | 1.14 | 0.68 |
| CA041902 | Elongation factor G 1 | 1.04 | 0.94 | 0.84 | 0.69 | 1.41 | 0.44 | 0.31 | 0.01 | 1.12 | 0.81 | 1.58 | 0.30 |
| CB498670 | Proteasome maturation protein | 0.33 | 0.02 | 0.82 | 0.64 | 1.53 | 0.33 | 0.16 | 0.00 | 0.89 | 0.79 | 2.04 | 0.11 |
| CB498104 | Si:dkey-78d16.1 protein [Danio rerio] | 1.40 | 0.52 | 0.23 | 0.00 | 0.98 | 0.97 | 1.26 | 0.66 | 0.38 | 0.10 | 1.32 | 0.61 |
| CA049006 | SUMO-activating enzyme subunit 2 | 0.74 | 0.11 | 2.30 | 0.00 | 1.02 | 0.93 | 0.59 | 0.01 | 1.33 | 0.19 | 0.88 | 0.50 |
| CA048635 | Peroxisomal NADH pyrophosphatase NUDT12 | 0.87 | 0.71 | 3.87 | 0.00 | 0.99 | 0.97 | 0.75 | 0.46 | 1.57 | 0.24 | 0.93 | 0.85 |
| CA050851 | Serine/threonine-protein kinase Nek6 | 0.71 | 0.24 | 2.13 | 0.00 | 1.03 | 0.91 | 1.41 | 0.25 | 1.22 | 0.43 | 1.03 | 0.93 |
| CA063605 | UNKNOWN | 0.91 | 0.56 | 1.65 | 0.01 | 1.13 | 0.46 | 0.88 | 0.40 | 1.33 | 0.13 | 1.13 | 0.45 |
| CA042392 | Glycerol-3-phosphate acyltransferase, | 0.67 | 0.10 | 1.00 | 1.00 | 1.21 | 0.42 | 1.04 | 0.88 | 1.15 | 0.53 | 1.88 | 0.01 |
| CB510882 | Intermediate filament protein ON3 | 0.37 | 0.00 | 0.64 | 0.14 | 1.15 | 0.64 | 0.84 | 0.56 | 1.12 | 0.70 | 1.24 | 0.45 |
| CB497475 | CDC5L, KIAA0432, PCDC5RP: Cell division cycle 5-like | 1.29 | 0.25 | 2.57 | 0.00 | 0.96 | 0.83 | 0.94 | 0.78 | 1.32 | 0.27 | 0.89 | 0.57 |
| CB515716 | Vesicular integral-membrane protein VIP36 precursor | 0.71 | 0.17 | 1.05 | 0.83 | 0.83 | 0.41 | 0.53 | 0.01 | 0.97 | 0.90 | 0.80 | 0.35 |
| CA060563 | UNKNOWN | 1.24 | 0.34 | 0.55 | 0.01 | 1.15 | 0.55 | 0.75 | 0.20 | 0.51 | 0.00 | 1.81 | 0.01 |
| CA060280 | UNKNOWN | 0.63 | 0.10 | 0.78 | 0.36 | 0.65 | 0.09 | 0.42 | 0.00 | 0.87 | 0.60 | 1.37 | 0.21 |
| CA040903 | L-fucose kinase | 1.42 | 0.21 | 0.46 | 0.01 | 1.23 | 0.47 | 1.22 | 0.48 | 0.61 | 0.06 | 1.55 | 0.13 |
| CB493361 | toxin-1 [Oncorhynchus mykiss] | 0.86 | 0.61 | 0.55 | 0.04 | 1.24 | 0.45 | 1.21 | 0.51 | 0.46 | 0.01 | 1.42 | 0.22 |
| CB507696 | Rhamnose-binding lectin precursor | 0.76 | 0.46 | 1.36 | 0.40 | 2.64 | 0.01 | 0.81 | 0.58 | 0.93 | 0.84 | 1.33 | 0.43 |
| CA063335 | UNKNOWN | 1.19 | 0.50 | 0.86 | 0.53 | 1.23 | 0.40 | 0.74 | 0.23 | 1.09 | 0.72 | 1.89 | 0.01 |
| CA047497 | SJCHGC05055 protein [Schistosoma japonicum] | 0.85 | 0.45 | 1.94 | 0.01 | 0.87 | 0.48 | 0.65 | 0.05 | 1.21 | 0.43 | 1.00 | 0.98 |
| CB505502 | Collagen alpha-1(I) chain precursor | 0.40 | 0.00 | 0.86 | 0.53 | 0.52 | 0.02 | 1.00 |  | 1.00 |  | 1.00 |  |
| CA044590 | 7-dehydrocholesterol reductase | 0.84 | 0.55 | 0.46 | 0.01 | 0.60 | 0.10 | 0.80 | 0.45 | 0.81 | 0.47 | 0.99 | 0.97 |
| CB492638 | FK506-binding protein 2 precursor | 0.65 | 0.03 | 0.78 | 0.16 | 1.05 | 0.83 | 0.59 | 0.01 | 1.08 | 0.65 | 1.49 | 0.07 |
| CB489719 | Actin, aortic smooth muscle | 0.94 | 0.84 | 0.38 | 0.00 | 0.90 | 0.73 | 1.71 | 0.10 | 1.14 | 0.67 | 1.22 | 0.52 |
| CB496456 | Proteasome subunit alpha type 3 | 0.42 | 0.00 | 0.64 | 0.10 | 1.36 | 0.28 | 0.70 | 0.17 | 1.13 | 0.64 | 1.09 | 0.77 |
| CA046405 | Plecoglossus altivelis 32.1 kDa connexin mRNA, complete | 3.06 | 0.01 | 0.82 | 0.64 | 1.52 | 0.36 | 1.59 | 0.32 | 1.27 | 0.57 | 1.54 | 0.34 |
| CA041804 | Hemoglobin subunit beta-1 | 1.04 | 0.81 | 1.38 | 0.04 | 0.60 | 0.03 | 1.46 | 0.04 | 0.83 | 0.25 | 0.45 | 0.00 |
| CB496913 | Parvalbumin, thymic | 1.00 | 0.99 | 0.54 | 0.03 | 0.98 | 0.94 | 0.99 | 0.98 | 0.44 | 0.01 | 1.02 | 0.96 |
| CK990752 | UNKNOWN | 1.02 | 0.96 | 0.33 | 0.00 | 1.85 | 0.13 | 1.33 | 0.43 | 0.63 | 0.21 | 1.01 | 0.98 |
| CA043217 | UNKNOWN | 0.94 | 0.87 | 0.83 | 0.64 | 1.20 | 0.70 | 1.23 | 0.62 | 0.23 | 0.00 | 1.10 | 0.83 |
| CB491826 | Glyceraldehyde-3-phosphate dehydrogenase, cytosolic 1 | 0.54 | 0.01 | 0.87 | 0.57 | 0.73 | 0.17 | 0.80 | 0.34 | 1.16 | 0.55 | 0.98 | 0.92 |
| BU965773 | Protein kinase C and casein kinase substrate | 2.14 | 0.01 | 1.14 | 0.62 | 0.77 | 0.27 | 2.01 | 0.02 | 1.15 | 0.60 | 1.05 | 0.83 |
| CA062869 | DNA-binding protein inhibitor ID-3 | 0.92 | 0.66 | 1.27 | 0.22 | 0.64 | 0.01 | 0.73 | 0.12 | 1.34 | 0.14 | 1.01 | 0.96 |
| CA042558 | UNKNOWN | 0.70 | 0.22 | 0.64 | 0.15 | 1.56 | 0.15 | 0.47 | 0.01 | 0.90 | 0.73 | 1.55 | 0.16 |
| CA064574 | Delta(3,5)-Delta(2,4)-dienoyl-CoA isomerase | 1.56 | 0.33 | 1.26 | 0.55 | 1.54 | 0.31 | 0.25 | 0.01 | 0.74 | 0.44 | 1.09 | 0.84 |
| CA058389 | Creatine kinase B-type | 0.63 | 0.11 | 0.48 | 0.01 | 0.69 | 0.19 | 1.02 | 0.94 | 0.85 | 0.56 | 1.02 | 0.95 |
| CB498091 | Coatomer subunit alpha | 2.05 | 0.02 | 0.42 | 0.01 | 0.97 | 0.91 | 0.98 | 0.94 | 0.52 | 0.05 | 1.95 | 0.04 |
| CA051333 | Developmentally-regulated RNA-binding protein 1 | 0.72 | 0.13 | 0.98 | 0.94 | 1.65 | 0.03 | 0.75 | 0.18 | 0.89 | 0.59 | 1.81 | 0.01 |
| CA042983 | Diamine acetyltransferase 1 | 0.90 | 0.75 | 0.98 | 0.95 | 2.79 | 0.00 | 0.90 | 0.76 | 1.13 | 0.75 | 2.11 | 0.03 |
| CB494479 | Transcription factor ETV6 | 2.54 | 0.01 | 0.73 | 0.40 | 0.72 | 0.37 | 1.38 | 0.36 | 1.11 | 0.78 | 0.85 | 0.67 |
| CB505559 | Phosphoglycerate kinase | 0.47 | 0.02 | 0.50 | 0.02 | 1.99 | 0.02 | 0.46 | 0.01 | 0.68 | 0.19 | 2.44 | 0.00 |
| CB510630 | UNKNOWN | 0.97 | 0.93 | 0.32 | 0.00 | 1.07 | 0.82 | 1.47 | 0.19 | 0.91 | 0.79 | 1.29 | 0.40 |
| CA063031 | Fibronectin type-III domain-containing protein 3a | 0.48 | 0.01 | 0.83 | 0.44 | 0.97 | 0.91 | 0.71 | 0.23 | 1.32 | 0.26 | 1.10 | 0.71 |
| CA057269 | Intraflagellar transport 52 homolog | 0.52 | 0.03 | 0.85 | 0.58 | 1.64 | 0.05 | 0.71 | 0.23 | 1.44 | 0.24 | 2.06 | 0.01 |
| CB492685 | Barrier-to-autointegration factor | 1.13 | 0.73 | 0.47 | 0.03 | 1.59 | 0.17 | 0.36 | 0.01 | 0.56 | 0.09 | 1.30 | 0.43 |
| CA769854 | Fatty acid-binding protein, heart | 0.81 | 0.39 | 0.91 | 0.69 | 1.43 | 0.12 | 0.68 | 0.11 | 1.07 | 0.78 | 1.82 | 0.01 |
| CA044021 | UNKNOWN | 0.96 | 0.90 | 0.39 | 0.01 | 2.21 | 0.03 | 1.23 | 0.56 | 0.76 | 0.39 | 2.89 | 0.00 |
| CA045260 | UNKNOWN | 1.22 | 0.47 | 0.64 | 0.13 | 1.76 | 0.06 | 0.89 | 0.68 | 0.64 | 0.13 | 2.88 | 0.00 |
| CA063447 | UNKNOWN | 1.01 | 0.97 | 0.43 | 0.00 | 0.76 | 0.24 | 1.10 | 0.66 | 0.61 | 0.02 | 1.14 | 0.58 |
| CB515607 | Eukaryotic initiation factor 4A-I | 0.69 | 0.28 | 1.23 | 0.53 | 2.66 | 0.01 | 0.53 | 0.07 | 1.44 | 0.28 | 3.51 | 0.00 |
| CB513814 | UNKNOWN | 1.23 | 0.47 | 3.25 | 0.00 | 0.98 | 0.93 | 0.80 | 0.42 | 0.78 | 0.35 | 0.77 | 0.35 |
| CA047762 | Transcription factor BTF3 homolog 4 | 1.62 | 0.16 | 0.38 | 0.01 | 0.43 | 0.02 | 1.28 | 0.47 | 0.66 | 0.22 | 0.68 | 0.29 |
| CB494389 | Creatine kinase M-type | 1.00 | 0.99 | 0.53 | 0.01 | 0.82 | 0.45 | 1.69 | 0.05 | 1.22 | 0.39 | 0.95 | 0.85 |
| CB493642 | Serine/threonine-protein kinase PINK1, | 0.94 | 0.80 | 0.60 | 0.01 | 0.87 | 0.50 | 1.01 | 0.96 | 0.92 | 0.68 | 1.27 | 0.23 |
| CB515642 | UNKNOWN | 0.73 | 0.30 | 0.36 | 0.00 | 0.94 | 0.84 | 0.76 | 0.37 | 0.53 | 0.04 | 1.61 | 0.14 |
| CB517814 | UNKNOWN | 0.91 | 0.74 | 0.43 | 0.01 | 1.35 | 0.33 | 1.58 | 0.12 | 0.73 | 0.28 | 2.17 | 0.02 |
| CA045222 | PREDICTED: similar to MGC82565 protein isoform 1 | 1.85 | 0.37 | 0.15 | 0.00 | 0.86 | 0.81 | 1.63 | 0.45 | 0.66 | 0.50 | 0.52 | 0.30 |
| CA063614 | UNKNOWN | 0.38 | 0.01 | 0.59 | 0.09 | 1.25 | 0.44 | 0.76 | 0.40 | 1.22 | 0.51 | 1.51 | 0.15 |
| CB507933 | DPH3 homolog | 1.75 | 0.11 | 0.68 | 0.28 | 1.25 | 0.49 | 1.31 | 0.49 | 0.42 | 0.01 | 0.97 | 0.93 |
| CA046429 | Pituitary tumor-transforming gene 1 protein-interacting | 1.22 | 0.61 | 0.45 | 0.02 | 2.25 | 0.04 | 1.10 | 0.83 | 0.43 | 0.02 | 3.76 | 0.00 |
| CA059508 | P2Y purinoceptor 8 | 0.58 | 0.05 | 0.43 | 0.00 | 0.77 | 0.21 | 0.40 | 0.00 | 1.21 | 0.36 | 0.94 | 0.76 |
| CB500424 | UNKNOWN | 0.61 | 0.09 | 0.51 | 0.01 | 0.67 | 0.18 | 0.75 | 0.32 | 0.75 | 0.26 | 1.19 | 0.56 |
| CA057485 | ATP-binding cassette sub-family F member 1 | 0.68 | 0.09 | 0.88 | 0.51 | 0.73 | 0.09 | 0.53 | 0.01 | 1.06 | 0.76 | 1.16 | 0.44 |
| CB509750 | HD domain-containing protein 3 | 0.85 | 0.38 | 0.65 | 0.02 | 1.72 | 0.01 | 0.68 | 0.03 | 0.76 | 0.14 | 0.99 | 0.95 |
| CB498293 | Creatine kinase B-type | 0.82 | 0.44 | 0.86 | 0.56 | 2.21 | 0.01 | 0.84 | 0.48 | 1.69 | 0.05 | 0.98 | 0.95 |
| CA052154 | Very low-density lipoprotein receptor precursor | 0.69 | 0.17 | 1.93 | 0.01 | 0.89 | 0.63 | 0.92 | 0.77 | 0.91 | 0.70 | 1.29 | 0.32 |
| CA054446 | Small ubiquitin-related modifier 3 precursor | 0.38 | 0.01 | 0.32 | 0.00 | 0.84 | 0.62 | 0.59 | 0.17 | 0.72 | 0.37 | 1.03 | 0.94 |
| CK990602 | SJCHGC04882 protein [Schistosoma japonicum] | 0.49 | 0.00 | 0.80 | 0.29 | 0.97 | 0.89 | 0.84 | 0.45 | 1.12 | 0.58 | 1.04 | 0.85 |
| CA056008 | UNKNOWN | 1.07 | 0.75 | 1.82 | 0.01 | 1.44 | 0.17 | 1.05 | 0.82 | 1.85 | 0.01 | 0.97 | 0.91 |
| CB516595 | Derlin-2 | 1.13 | 0.68 | 2.28 | 0.01 | 0.90 | 0.70 | 1.00 | 1.00 | 1.40 | 0.24 | 1.37 | 0.28 |
| CA042998 | UNKNOWN | 0.66 | 0.10 | 0.75 | 0.28 | 1.37 | 0.23 | 0.49 | 0.01 | 0.83 | 0.46 | 1.17 | 0.54 |
| CA768205 | PREDICTED: Danio rerio hypothetical | 0.84 | 0.45 | 0.51 | 0.01 | 0.93 | 0.78 | 0.90 | 0.65 | 0.90 | 0.63 | 1.32 | 0.29 |
| CA060800 | PREDICTED: similar to renal organic anion transporter | 1.06 | 0.89 | 0.22 | 0.00 | 0.60 | 0.16 | 1.29 | 0.53 | 0.34 | 0.01 | 1.12 | 0.75 |
| CB496960 | 52 kDa repressor of the inhibitor of the protein kinase | 0.28 | 0.00 | 0.70 | 0.29 | 1.34 | 0.39 | 0.45 | 0.03 | 0.63 | 0.19 | 1.42 | 0.31 |
| CB494616 | Uncharacterized protein C1orf52 homolog | 1.27 | 0.34 | 0.53 | 0.01 | 0.89 | 0.58 | 0.85 | 0.53 | 0.60 | 0.03 | 1.38 | 0.13 |
| CB509951 | Integral membrane protein 2B | 1.68 | 0.01 | 1.02 | 0.94 | 0.97 | 0.87 | 1.31 | 0.18 | 0.93 | 0.75 | 1.23 | 0.30 |
| CA057637 | Importin-7 | 1.18 | 0.55 | 0.56 | 0.01 | 0.80 | 0.34 | 0.98 | 0.95 | 0.71 | 0.13 | 1.44 | 0.14 |
| CA051997 | UNKNOWN | 1.05 | 0.82 | 1.76 | 0.01 | 0.90 | 0.61 | 0.62 | 0.05 | 1.09 | 0.70 | 0.92 | 0.70 |
| CA048055 | Electron transfer flavoprotein-ubiquinone oxidoreductase, | 1.20 | 0.39 | 1.92 | 0.01 | 0.96 | 0.86 | 1.11 | 0.62 | 1.94 | 0.01 | 0.78 | 0.24 |
| CK991193 | Proteasome subunit beta type 6 precursor | 0.62 | 0.08 | 0.87 | 0.61 | 1.34 | 0.28 | 0.49 | 0.01 | 0.91 | 0.71 | 0.99 | 0.96 |
| CA044407 | Oncorhynchus mykiss MHC class Ib antigen | 1.97 | 0.00 | 0.80 | 0.36 | 0.82 | 0.40 | 0.88 | 0.58 | 0.74 | 0.20 | 1.08 | 0.75 |
| CA052033 | UNKNOWN | 1.13 | 0.65 | 1.65 | 0.07 | 0.64 | 0.08 | 1.00 | 1.00 | 1.48 | 0.15 | 0.49 | 0.01 |
| CK990492 | Sodium/potassium-transporting ATPase subunit alpha-1 | 0.92 | 0.71 | 0.60 | 0.03 | 0.94 | 0.79 | 0.61 | 0.04 | 0.51 | 0.00 | 1.12 | 0.62 |
| CB492698 | Microsomal glutathione S-transferase 3 | 0.82 | 0.36 | 1.10 | 0.66 | 1.16 | 0.51 | 0.51 | 0.00 | 1.07 | 0.78 | 1.14 | 0.55 |
| CB511458 | Transforming protein RhoA precursor | 0.81 | 0.51 | 0.90 | 0.71 | 0.88 | 0.66 | 0.82 | 0.53 | 0.46 | 0.01 | 1.16 | 0.59 |
| CA060215 | Targeting protein for Xklp2 | 0.70 | 0.07 | 1.59 | 0.03 | 0.52 | 0.00 | 0.73 | 0.11 | 1.75 | 0.01 | 1.22 | 0.32 |
| CA043333 | Inositol-trisphosphate 3-kinase A | 0.90 | 0.54 | 1.57 | 0.01 | 1.02 | 0.92 | 1.04 | 0.84 | 1.48 | 0.03 | 1.10 | 0.61 |
| CA063139 | UNKNOWN | 0.86 | 0.60 | 2.19 | 0.01 | 0.85 | 0.57 | 0.64 | 0.13 | 1.55 | 0.12 | 1.79 | 0.04 |
| CA043517 | Protein ariadne-2 homolog | 1.11 | 0.74 | 1.48 | 0.23 | 0.86 | 0.64 | 0.85 | 0.61 | 1.06 | 0.85 | 2.31 | 0.01 |
| CA054819 | Platelet-activating factor acetylhydrolase precursor | 1.00 | 0.99 | 2.09 | 0.00 | 0.93 | 0.76 | 0.98 | 0.93 | 1.36 | 0.20 | 0.87 | 0.54 |
| CB500554 | surface protein PspC [Streptococcus pneumoniae] | 1.00 | 0.99 | 0.84 | 0.53 | 1.52 | 0.11 | 0.91 | 0.72 | 2.55 | 0.00 | 1.27 | 0.35 |
| CB501803 | UNKNOWN | 1.34 | 0.23 | 0.52 | 0.01 | 1.14 | 0.56 | 1.07 | 0.78 | 1.01 | 0.96 | 1.25 | 0.32 |
| CA041783 | UNKNOWN | 0.45 | 0.00 | 0.65 | 0.05 | 1.05 | 0.82 | 0.71 | 0.16 | 0.80 | 0.31 | 1.06 | 0.78 |
| CA057987 | Triosephosphate isomerase | 0.77 | 0.32 | 1.22 | 0.43 | 0.92 | 0.73 | 0.44 | 0.00 | 1.39 | 0.21 | 1.11 | 0.68 |
| CA063770 | UNKNOWN | 1.30 | 0.43 | 0.41 | 0.00 | 0.71 | 0.24 | 1.88 | 0.06 | 0.67 | 0.19 | 1.08 | 0.79 |
| CB499801 | Pre-B-cell leukemia transcription factor 2 | 1.10 | 0.61 | 0.61 | 0.01 | 1.07 | 0.70 | 1.05 | 0.77 | 1.12 | 0.54 | 1.43 | 0.05 |
| CA047650 | UNKNOWN | 1.03 | 0.94 | 0.37 | 0.01 | 0.59 | 0.11 | 1.32 | 0.37 | 0.62 | 0.18 | 0.96 | 0.90 |
| CB517071 | Exosome complex exonuclease RRP4 | 0.47 | 0.00 | 0.85 | 0.31 | 0.99 | 0.95 | 0.67 | 0.06 | 1.26 | 0.17 | 1.44 | 0.03 |
| CB512317 | Coiled-coil domain-containing protein 43 | 0.93 | 0.71 | 0.99 | 0.95 | 0.77 | 0.19 | 0.65 | 0.03 | 1.61 | 0.01 | 1.66 | 0.01 |
| CB496526 | Troponin I, fast skeletal muscle | 2.66 | 0.01 | 0.90 | 0.79 | 0.93 | 0.87 | 0.54 | 0.12 | 1.14 | 0.74 | 1.64 | 0.25 |
| CA060592 | Stonustoxin subunit beta | 0.63 | 0.12 | 0.45 | 0.00 | 0.95 | 0.84 | 0.98 | 0.95 | 0.73 | 0.23 | 1.00 | 1.00 |
| CB509084 | Tropomyosin-1 alpha chain | 0.66 | 0.17 | 0.46 | 0.01 | 0.78 | 0.45 | 0.93 | 0.81 | 0.91 | 0.76 | 0.92 | 0.80 |
| CA050552 | UNKNOWN | 1.32 | 0.37 | 3.02 | 0.00 | 0.76 | 0.38 | 0.97 | 0.93 | 1.79 | 0.11 | 0.86 | 0.62 |
| CB496860 | NADH dehydrogenase [ubiquinone] 1 alpha subcomplex | 1.29 | 0.33 | 0.66 | 0.08 | 0.75 | 0.22 | 0.85 | 0.53 | 0.45 | 0.00 | 0.88 | 0.58 |
| CB493848 | Protein ZNF452 | 1.35 | 0.25 | 0.48 | 0.01 | 1.35 | 0.30 | 0.97 | 0.89 | 0.48 | 0.01 | 1.37 | 0.28 |
| CA053524 | Programmed cell death protein 10 | 0.92 | 0.73 | 2.02 | 0.01 | 0.96 | 0.86 | 1.09 | 0.72 | 1.45 | 0.16 | 1.53 | 0.11 |
| CB498959 | EH domain-containing protein 1 | 1.33 | 0.28 | 2.15 | 0.01 | 1.11 | 0.67 | 1.07 | 0.80 | 1.36 | 0.24 | 1.41 | 0.16 |
| CA048825 | BOLA class I histocompatibility antigen, alpha chain BL3-7 | 0.77 | 0.36 | 0.46 | 0.01 | 0.73 | 0.31 | 1.14 | 0.65 | 1.09 | 0.76 | 1.15 | 0.65 |
| CA052231 | UV excision repair protein RAD23 homolog B | 1.48 | 0.13 | 0.48 | 0.01 | 1.05 | 0.82 | 1.10 | 0.71 | 0.84 | 0.48 | 0.96 | 0.86 |
| CA051623 | Ephexin-1 | 0.85 | 0.66 | 2.73 | 0.01 | 0.77 | 0.48 | 0.73 | 0.40 | 1.83 | 0.08 | 0.95 | 0.89 |
| CA037712 | UNKNOWN | 1.00 | 0.99 | 1.13 | 0.76 | 1.38 | 0.47 | 3.18 | 0.01 | 0.68 | 0.34 | 1.47 | 0.38 |
| CA048973 | Protein disulfide-isomerase A4 precursor | 2.08 | 0.00 | 1.04 | 0.86 | 1.08 | 0.75 | 1.48 | 0.10 | 1.01 | 0.96 | 1.25 | 0.38 |
| CA051713 | UNKNOWN | 1.01 | 0.95 | 1.81 | 0.00 | 0.96 | 0.85 | 1.00 | 1.00 | 1.91 | 0.00 | 0.98 | 0.91 |
| CB510789 | selenoprotein W1 [Danio rerio] | 1.69 | 0.25 | 0.31 | 0.00 | 1.08 | 0.85 | 1.02 | 0.95 | 1.60 | 0.19 | 0.80 | 0.57 |
| CA043734 | Copper transport protein ATOX1 | 2.62 | 0.00 | 1.69 | 0.02 | 1.10 | 0.66 | 0.98 | 0.92 | 1.50 | 0.07 | 1.05 | 0.81 |
| CA053425 | Polymerase delta-interacting protein 2 | 1.28 | 0.21 | 0.84 | 0.33 | 0.53 | 0.00 | 1.32 | 0.16 | 1.06 | 0.74 | 0.96 | 0.84 |
| CB493171 | Glutamine synthetase | 1.88 | 0.21 | 0.26 | 0.01 | 1.06 | 0.92 | 1.00 | 1.00 | 0.81 | 0.68 | 0.79 | 0.69 |
| CA052620 | Plasma membrane calcium-transporting ATPase 3 | 0.45 | 0.01 | 0.98 | 0.93 | 0.91 | 0.68 | 0.32 | 0.00 | 0.94 | 0.82 | 0.99 | 0.96 |
| CA053558 | High affinity interleukin-8 receptor A | 1.23 | 0.38 | 0.74 | 0.16 | 0.65 | 0.08 | 1.98 | 0.01 | 0.77 | 0.22 | 0.80 | 0.36 |
| CA051591 | Splicing factor, proline- and glutamine-rich | 1.07 | 0.74 | 0.77 | 0.18 | 0.56 | 0.01 | 1.19 | 0.37 | 1.02 | 0.91 | 1.09 | 0.69 |
| CK991277 | UNKNOWN | 0.66 | 0.09 | 0.56 | 0.01 | 0.84 | 0.48 | 0.97 | 0.91 | 0.91 | 0.66 | 1.04 | 0.88 |
| CB497970 | Complement C3-1 | 0.69 | 0.27 | 0.48 | 0.03 | 1.03 | 0.93 | 0.39 | 0.01 | 0.68 | 0.24 | 1.27 | 0.49 |
| CA064190 | Coiled-coil domain-containing protein 127 | 1.00 | 0.99 | 2.11 | 0.01 | 1.00 | 0.99 | 1.22 | 0.51 | 1.85 | 0.04 | 1.09 | 0.78 |
| CB498429 | Baculoviral IAP repeat-containing protein 6 | 0.69 | 0.24 | 0.92 | 0.80 | 1.11 | 0.71 | 0.68 | 0.20 | 0.81 | 0.51 | 2.08 | 0.01 |
| CB505799 | Translation machinery-associated protein 20 | 0.97 | 0.93 | 0.67 | 0.35 | 1.28 | 0.52 | 1.43 | 0.38 | 0.40 | 0.02 | 2.77 | 0.01 |
| CB511865 | UNKNOWN | 0.70 | 0.14 | 1.98 | 0.00 | 0.95 | 0.82 | 1.18 | 0.49 | 1.10 | 0.67 | 1.10 | 0.66 |
| CA041584 | Uncharacterized protein C20orf24 homolog | 3.18 | 0.00 | 0.66 | 0.27 | 0.96 | 0.92 | 1.66 | 0.19 | 0.79 | 0.53 | 1.33 | 0.45 |
| CA061354 | UNKNOWN | 0.67 | 0.32 | 1.02 | 0.96 | 3.48 | 0.01 | 0.54 | 0.13 | 1.64 | 0.21 | 3.04 | 0.02 |
| CA047068 | Sodium/potassium-transporting ATPase subunit beta-3 | 0.67 | 0.46 | 0.64 | 0.31 | 1.59 | 0.25 | 0.23 | 0.00 | 0.88 | 0.76 | 1.21 | 0.63 |
| CB501837 | Glyceraldehyde-3-phosphate dehydrogenase | 0.77 | 0.37 | 0.48 | 0.01 | 1.49 | 0.22 | 0.91 | 0.74 | 0.70 | 0.22 | 1.01 | 0.97 |
| CK991084 | UNKNOWN | 1.38 | 0.54 | 0.16 | 0.01 | 1.08 | 0.91 | 1.43 | 0.53 | 0.57 | 0.40 | 1.67 | 0.44 |
| CA044864 | Malate dehydrogenase, cytoplasmic | 0.51 | 0.01 | 0.61 | 0.07 | 0.90 | 0.63 | 0.55 | 0.02 | 1.10 | 0.72 | 1.14 | 0.55 |
| CA056647 | Iron(III)-zinc(II) purple acid phosphatase precursor | 1.04 | 0.89 | 0.51 | 0.01 | 0.80 | 0.41 | 1.18 | 0.55 | 0.70 | 0.18 | 1.15 | 0.60 |
| CB497894 | Fibrinogen beta chain precursor | 1.67 | 0.29 | 0.24 | 0.00 | 0.92 | 0.87 | 0.99 | 0.98 | 0.43 | 0.08 | 2.13 | 0.15 |
| CB511204 | UNKNOWN | 1.30 | 0.55 | 0.22 | 0.00 | 1.26 | 0.62 | 1.71 | 0.23 | 0.56 | 0.19 | 1.50 | 0.39 |
| CB504375 | PREDICTED: similar to Myh11 protein [Danio rerio] | 0.87 | 0.71 | 0.33 | 0.01 | 1.30 | 0.46 | 1.14 | 0.72 | 0.40 | 0.04 | 1.80 | 0.10 |
| CB502589 | UNKNOWN | 1.17 | 0.74 | 0.28 | 0.01 | 0.68 | 0.42 | 1.52 | 0.38 | 0.35 | 0.03 | 0.83 | 0.69 |
| CB509406 | Phosphoribosyl pyrophosphate synthetase-associated | 1.27 | 0.48 | 0.88 | 0.73 | 2.45 | 0.02 | 1.13 | 0.72 | 1.83 | 0.10 | 4.04 | 0.00 |
| CK990520 | UNKNOWN | 1.47 | 0.16 | 0.42 | 0.00 | 1.10 | 0.72 | 0.84 | 0.53 | 0.55 | 0.02 | 1.27 | 0.39 |
| CA044186 | UNKNOWN | 1.11 | 0.76 | 0.40 | 0.01 | 0.95 | 0.87 | 0.99 | 0.99 | 0.79 | 0.46 | 1.21 | 0.53 |
| CA042416 | Salmo salar zonadhesin-like gene, complete cds and 3' | 1.40 | 0.58 | 0.28 | 0.01 | 0.59 | 0.29 | 1.27 | 0.66 | 0.45 | 0.11 | 0.85 | 0.74 |
| CB493689 | Protein kinase C-binding protein NELL2 precursor | 1.66 | 0.06 | 1.16 | 0.56 | 1.09 | 0.75 | 0.45 | 0.00 | 0.84 | 0.50 | 0.87 | 0.62 |
| CB511043 | LIM domain-binding protein 3 | 0.80 | 0.52 | 0.33 | 0.00 | 0.82 | 0.53 | 0.86 | 0.65 | 0.98 | 0.96 | 1.07 | 0.83 |
| CA064173 | Cell cycle control protein 50A | 0.88 | 0.65 | 0.49 | 0.01 | 0.77 | 0.37 | 0.90 | 0.69 | 0.88 | 0.64 | 1.11 | 0.71 |
| CK990936 | UNKNOWN | 0.79 | 0.67 | 0.53 | 0.25 | 1.16 | 0.77 | 0.53 | 0.28 | 0.19 | 0.00 | 1.76 | 0.27 |
| CA058822 | Spectrin beta chain, brain 1 | 0.51 | 0.01 | 0.96 | 0.85 | 0.69 | 0.11 | 0.77 | 0.29 | 1.08 | 0.76 | 1.14 | 0.57 |
| CA045825 | COMM domain-containing protein 6 | 0.47 | 0.01 | 0.52 | 0.01 | 0.85 | 0.46 | 0.68 | 0.15 | 1.14 | 0.56 | 1.21 | 0.41 |
| CA062226 | RP11-297I6.1 [Homo sapiens] | 1.43 | 0.31 | 0.33 | 0.00 | 0.66 | 0.25 | 1.10 | 0.78 | 0.42 | 0.02 | 1.90 | 0.08 |
| CA064001 | UNKNOWN | 1.35 | 0.19 | 0.82 | 0.34 | 0.57 | 0.01 | 1.01 | 0.95 | 1.01 | 0.96 | 1.17 | 0.46 |
| CA037988 | Pachymedusa dacnicolor partial mRNA for ribosomal | 0.71 | 0.18 | 0.45 | 0.00 | 0.77 | 0.27 | 0.75 | 0.27 | 1.24 | 0.35 | 0.91 | 0.68 |
| CA055512 | UNKNOWN | 0.94 | 0.87 | 0.37 | 0.01 | 0.75 | 0.41 | 0.93 | 0.85 | 0.53 | 0.09 | 1.35 | 0.39 |
| CA061144 | UNKNOWN | 0.76 | 0.43 | 0.42 | 0.01 | 0.92 | 0.81 | 1.10 | 0.79 | 0.72 | 0.32 | 1.24 | 0.55 |
| CA053211 | Ras-related protein Rab-28 | 1.12 | 0.63 | 0.83 | 0.42 | 0.97 | 0.92 | 0.44 | 0.00 | 0.69 | 0.12 | 1.01 | 0.96 |
| CA063981 | NAD(P) transhydrogenase, mitochondrial precursor | 0.85 | 0.56 | 1.38 | 0.26 | 0.88 | 0.64 | 0.40 | 0.00 | 1.08 | 0.79 | 1.12 | 0.68 |
| CA043772 | UNKNOWN | 0.60 | 0.11 | 0.42 | 0.01 | 0.92 | 0.76 | 0.77 | 0.42 | 1.13 | 0.70 | 1.10 | 0.75 |
| CA059430 | Histone-binding protein RBBP7 | 0.79 | 0.49 | 0.47 | 0.01 | 0.86 | 0.60 | 0.63 | 0.19 | 1.13 | 0.68 | 1.01 | 0.97 |
| CB493305 | 60S ribosomal protein L24 | 0.74 | 0.32 | 0.48 | 0.01 | 0.83 | 0.54 | 0.92 | 0.78 | 1.11 | 0.70 | 1.08 | 0.80 |
| CA051646 | UNKNOWN | 0.74 | 0.26 | 0.47 | 0.01 | 0.96 | 0.89 | 0.66 | 0.12 | 0.88 | 0.61 | 1.03 | 0.90 |
| CK991176 | Alpha-enolase | 0.85 | 0.52 | 0.30 | 0.00 | 1.09 | 0.75 | 0.88 | 0.61 | 0.77 | 0.33 | 1.24 | 0.43 |
| CB512123 | PREDICTED: Danio rerio similar to Zinc finger and BTB | 0.87 | 0.68 | 2.93 | 0.00 | 0.59 | 0.12 | 0.99 | 0.97 | 1.44 | 0.28 | 1.06 | 0.86 |
| CA045168 | Olfactomedin precursor | 1.13 | 0.49 | 1.55 | 0.01 | 0.92 | 0.64 | 1.43 | 0.05 | 1.37 | 0.06 | 1.16 | 0.41 |
| CA057204 | Interleukin-31 receptor A precursor | 1.78 | 0.01 | 1.84 | 0.00 | 0.57 | 0.01 | 1.21 | 0.37 | 1.93 | 0.00 | 0.86 | 0.45 |
| CB498234 | Cytochrome c oxidase subunit VIIa-related protein, | 2.84 | 0.00 | 1.03 | 0.92 | 1.21 | 0.49 | 0.99 | 0.97 | 0.92 | 0.77 | 0.78 | 0.36 |
| CB496784 | Laminin subunit beta-2 precursor | 3.50 | 0.01 | 1.03 | 0.95 | 1.08 | 0.86 | 1.49 | 0.37 | 1.28 | 0.57 | 0.90 | 0.81 |
| CB494112 | Ribosome-binding protein 1 | 1.67 | 0.04 | 0.96 | 0.85 | 1.08 | 0.74 | 1.15 | 0.55 | 1.89 | 0.01 | 1.13 | 0.61 |
| CA057516 | Cytochrome P450 4F3 | 1.52 | 0.09 | 1.75 | 0.03 | 0.78 | 0.28 | 1.04 | 0.86 | 1.99 | 0.01 | 0.94 | 0.80 |
| CB510621 | UNKNOWN | 0.51 | 0.01 | 0.62 | 0.06 | 0.98 | 0.93 | 0.92 | 0.74 | 1.20 | 0.46 | 1.18 | 0.53 |
| CA054565 | Poly [ADP-ribose] polymerase 6 | 1.35 | 0.22 | 2.41 | 0.00 | 0.60 | 0.02 | 0.72 | 0.18 | 1.51 | 0.10 | 1.15 | 0.52 |
| CB512514 | Ubiquitin-conjugating enzyme E2 L3 | 0.95 | 0.88 | 0.95 | 0.87 | 0.82 | 0.48 | 1.20 | 0.54 | 2.22 | 0.01 | 1.66 | 0.08 |
| CA063416 | Nuclear cap-binding protein subunit 2 | 1.06 | 0.76 | 1.29 | 0.13 | 0.52 | 0.00 | 0.88 | 0.51 | 0.98 | 0.89 | 0.89 | 0.52 |
| CA048406 | Legumain precursor | 1.26 | 0.50 | 2.21 | 0.01 | 0.64 | 0.14 | 2.06 | 0.04 | 1.44 | 0.22 | 0.85 | 0.57 |
| CB507529 | UNKNOWN | 1.20 | 0.58 | 2.33 | 0.01 | 1.10 | 0.82 | 1.26 | 0.50 | 1.20 | 0.57 | 1.35 | 0.47 |
| CA037365 | Very-long-chain acyl-CoA synthetase | 1.11 | 0.75 | 2.51 | 0.01 | 0.74 | 0.38 | 0.99 | 0.97 | 1.00 | 1.00 | 1.08 | 0.83 |
| CK990246 | Phosphatidic acid phosphatase type 2 domain-containing | 1.49 | 0.46 | 1.05 | 0.92 | 0.18 | 0.01 | 0.99 | 0.99 | 1.50 | 0.41 | 2.06 | 0.26 |
| CA063526 | Oncorhynchus mykiss G-protein (P-ras) mRNA, complete | 1.20 | 0.54 | 0.39 | 0.01 | 1.08 | 0.80 | 1.12 | 0.70 | 0.86 | 0.65 | 0.91 | 0.77 |
| CA058100 | Stathmin | 1.00 | 0.99 | 0.55 | 0.01 | 0.81 | 0.33 | 0.73 | 0.15 | 0.94 | 0.78 | 1.00 | 0.98 |
| CA770586 | Formin-like protein 1 | 2.08 | 0.13 | 1.92 | 0.16 | 0.72 | 0.48 | 3.51 | 0.01 | 0.78 | 0.59 | 0.85 | 0.72 |
| CN442539 | Proteasome subunit beta type 7 precursor | 1.26 | 0.48 | 2.28 | 0.01 | 0.72 | 0.30 | 1.31 | 0.40 | 1.41 | 0.28 | 0.99 | 0.98 |
| CA052415 | Prohibitin-2 | 0.93 | 0.78 | 2.47 | 0.00 | 0.80 | 0.41 | 1.31 | 0.29 | 2.44 | 0.00 | 1.02 | 0.95 |
| CA054307 | S-adenosylmethionine synthetase isoform type-2 | 0.84 | 0.25 | 1.10 | 0.55 | 0.66 | 0.02 | 0.81 | 0.18 | 1.52 | 0.01 | 0.87 | 0.39 |
| CB509737 | LIM domain-binding protein 3 | 2.18 | 0.01 | 0.79 | 0.38 | 0.90 | 0.70 | 1.59 | 0.10 | 1.17 | 0.55 | 1.23 | 0.43 |
| CA050114 | Myosin-9 | 0.90 | 0.65 | 1.00 | 0.99 | 1.37 | 0.16 | 1.22 | 0.38 | 2.11 | 0.00 | 0.84 | 0.45 |
| CA039066 | Tripeptidyl-peptidase 1 precursor | 3.08 | 0.05 | 0.92 | 0.89 | 1.17 | 0.77 | 1.08 | 0.90 | 0.16 | 0.01 | 2.03 | 0.19 |
| CA042788 | Oncorhynchus mykiss SYPG1 (SYPG1), PHF1 (PHF1 | 1.05 | 0.83 | 0.58 | 0.01 | 0.76 | 0.20 | 1.26 | 0.30 | 0.94 | 0.75 | 0.74 | 0.16 |
| CB514304 | UNKNOWN | 1.32 | 0.43 | 0.38 | 0.01 | 1.00 | 1.00 | 0.64 | 0.21 | 0.59 | 0.11 | 1.36 | 0.45 |
| CA042670 | NEDD4 family-interacting protein 1 | 0.70 | 0.19 | 0.76 | 0.30 | 0.83 | 0.45 | 0.73 | 0.21 | 1.11 | 0.67 | 0.53 | 0.01 |
| CK990799 | Salmo salar clone BE7 beta-2 microglobulin (B2m) mRNA, | 1.00 | 1.00 | 2.66 | 0.00 | 0.82 | 0.44 | 0.88 | 0.64 | 1.75 | 0.03 | 1.02 | 0.95 |
| CA062651 | UNKNOWN | 0.65 | 0.16 | 0.46 | 0.01 | 0.66 | 0.21 | 1.10 | 0.76 | 0.58 | 0.06 | 0.78 | 0.45 |
| CA064455 | UNKNOWN | 0.67 | 0.12 | 2.50 | 0.00 | 0.74 | 0.22 | 1.06 | 0.82 | 1.43 | 0.12 | 0.82 | 0.41 |
| CK991356 | Transgelin | 0.58 | 0.04 | 0.40 | 0.00 | 1.11 | 0.68 | 0.98 | 0.93 | 0.76 | 0.33 | 1.37 | 0.21 |
| CA055494 | hCG32827, isoform CRA_c [Homo sapiens] | 1.10 | 0.70 | 2.29 | 0.00 | 0.76 | 0.30 | 1.10 | 0.69 | 1.56 | 0.10 | 0.91 | 0.71 |
| CB510635 | UNKNOWN | 1.83 | 0.03 | 2.01 | 0.01 | 1.00 | 1.00 | 1.07 | 0.80 | 1.92 | 0.02 | 0.81 | 0.39 |
| CB491775 | Geminin | 1.14 | 0.69 | 2.59 | 0.00 | 0.97 | 0.93 | 1.16 | 0.65 | 1.72 | 0.09 | 1.40 | 0.29 |
| CA059047 | Uncharacterized protein C1orf187 precursor | 1.05 | 0.85 | 1.41 | 0.20 | 1.26 | 0.39 | 0.53 | 0.01 | 1.41 | 0.20 | 1.13 | 0.63 |
| CB500540 | UNKNOWN | 1.15 | 0.63 | 0.67 | 0.15 | 1.07 | 0.80 | 2.16 | 0.01 | 0.91 | 0.73 | 1.14 | 0.64 |
| CB517843 | Oncorhynchus mykiss Onmy-LDA gene for MHC class I | 0.42 | 0.00 | 0.64 | 0.07 | 0.87 | 0.60 | 0.59 | 0.04 | 0.85 | 0.50 | 1.31 | 0.32 |
| CB514508 | Ependymin-2 precursor | 0.77 | 0.53 | 1.14 | 0.76 | 1.11 | 0.79 | 0.30 | 0.01 | 0.97 | 0.94 | 0.94 | 0.87 |
| CK990672 | UNKNOWN | 2.71 | 0.01 | 0.48 | 0.04 | 1.17 | 0.64 | 1.68 | 0.15 | 0.57 | 0.11 | 1.06 | 0.87 |
| CA058013 | RING finger protein 180 | 1.17 | 0.63 | 0.36 | 0.01 | 0.99 | 0.99 | 1.15 | 0.67 | 0.57 | 0.10 | 1.27 | 0.41 |
| CA038719 | Complement C3-1 | 1.37 | 0.40 | 0.47 | 0.04 | 1.44 | 0.31 | 3.41 | 0.00 | 0.42 | 0.02 | 1.31 | 0.45 |
| CA062067 | Protein SOF1 | 1.36 | 0.49 | 0.32 | 0.01 | 1.19 | 0.73 | 0.96 | 0.92 | 0.52 | 0.11 | 0.97 | 0.96 |
| CA064573 | Guanine nucleotide-binding protein G(i), alpha-2 subunit | 2.19 | 0.01 | 1.51 | 0.24 | 0.96 | 0.90 | 0.91 | 0.75 | 0.92 | 0.81 | 1.00 | 0.99 |
| CB510226 | Parvalbumin-2 | 0.48 | 0.32 | 0.74 | 0.61 | 0.98 | 0.97 | 7.51 | 0.01 | 0.51 | 0.26 | 1.44 | 0.54 |
| CA043931 | cAMP-dependent protein kinase type II-alpha regulatory | 0.69 | 0.29 | 0.32 | 0.00 | 1.03 | 0.92 | 0.60 | 0.15 | 0.73 | 0.35 | 1.64 | 0.14 |
| CA057032 | High mobility group protein B1 | 0.44 | 0.00 | 0.44 | 0.00 | 0.97 | 0.90 | 0.67 | 0.11 | 0.84 | 0.43 | 1.26 | 0.33 |
| CB493710 | PREDICTED: Danio rerio hypothetical LOC556945 | 0.75 | 0.40 | 0.39 | 0.00 | 1.15 | 0.64 | 0.74 | 0.35 | 0.64 | 0.15 | 2.14 | 0.02 |
| CB508304 | rRNA-processing protein FCF1 homolog | 1.19 | 0.61 | 0.49 | 0.04 | 1.44 | 0.27 | 1.38 | 0.35 | 0.33 | 0.00 | 1.81 | 0.08 |
| CA058670 | Stabilin-2 precursor | 1.17 | 0.73 | 0.19 | 0.00 | 0.91 | 0.82 | 1.03 | 0.94 | 0.30 | 0.00 | 1.12 | 0.77 |
| CA054281 | S.salar genes encoding alpha-globin and beta-globin, | 0.87 | 0.42 | 1.75 | 0.01 | 1.12 | 0.52 | 0.79 | 0.17 | 1.26 | 0.26 | 0.97 | 0.84 |
| CB511307 | Lipocalin precursor | 0.65 | 0.07 | 0.55 | 0.01 | 0.80 | 0.36 | 0.54 | 0.01 | 0.56 | 0.01 | 0.82 | 0.41 |
| CA042703 | UNKNOWN | 0.51 | 0.06 | 0.40 | 0.01 | 0.86 | 0.53 | 1.16 | 0.67 | 0.65 | 0.17 | 1.23 | 0.39 |
| CB493496 | PREDICTED: similar to adrenal gland protein AD-002 [ | 1.00 | 1.00 | 0.45 | 0.00 | 0.84 | 0.53 | 1.22 | 0.42 | 0.91 | 0.71 | 1.16 | 0.58 |
| CA057536 | Lin-7 homolog B | 1.62 | 0.01 | 1.14 | 0.46 | 0.79 | 0.18 | 1.06 | 0.73 | 0.92 | 0.62 | 1.14 | 0.46 |
| CA057034 | UNKNOWN | 1.67 | 0.01 | 0.78 | 0.26 | 0.76 | 0.15 | 1.29 | 0.19 | 0.92 | 0.71 | 1.15 | 0.46 |
| CB510654 | UNKNOWN | 1.50 | 0.56 | 0.09 | 0.00 | 1.83 | 0.34 | 3.85 | 0.07 | 0.34 | 0.12 | 0.78 | 0.69 |
| CB515945 | RNA-binding protein 5 | 0.98 | 0.95 | 0.94 | 0.83 | 1.54 | 0.17 | 1.13 | 0.69 | 1.23 | 0.46 | 2.37 | 0.01 |
| CA045255 | UNKNOWN | 0.82 | 0.76 | 0.14 | 0.00 | 1.50 | 0.51 | 0.53 | 0.33 | 0.43 | 0.18 | 1.39 | 0.60 |
| CA056706 | Tektin-4 | 1.39 | 0.52 | 0.18 | 0.00 | 1.00 | 1.00 | 1.55 | 0.40 | 0.33 | 0.03 | 1.65 | 0.31 |
| CB517135 | Probable E3 ubiquitin-protein ligase HERC2 | 1.26 | 0.51 | 0.38 | 0.01 | 1.35 | 0.39 | 1.13 | 0.73 | 0.55 | 0.09 | 1.52 | 0.23 |
| CB507931 | Ubiquitin-conjugating enzyme E2 G1 | 1.36 | 0.48 | 0.23 | 0.00 | 0.85 | 0.69 | 0.84 | 0.69 | 0.72 | 0.48 | 1.17 | 0.71 |
| CA047472 | Oncorhynchus nerka isolate pG-ON6-11 transposon Tc1- | 2.28 | 0.01 | 0.57 | 0.06 | 0.64 | 0.10 | 1.44 | 0.22 | 0.91 | 0.75 | 1.16 | 0.58 |
| CB509569 | Sarcoplasmic/endoplasmic reticulum calcium ATPase 1 | 0.80 | 0.46 | 0.41 | 0.01 | 0.53 | 0.05 | 0.85 | 0.59 | 1.36 | 0.32 | 1.05 | 0.88 |
| CA048910 | UNKNOWN | 1.05 | 0.81 | 1.06 | 0.72 | 0.81 | 0.31 | 1.11 | 0.57 | 0.96 | 0.83 | 1.76 | 0.01 |
| CA041782 | Putative serine protease K12H4.7 precursor | 1.35 | 0.44 | 2.72 | 0.01 | 0.98 | 0.97 | 1.30 | 0.49 | 1.15 | 0.71 | 0.98 | 0.96 |
| CA054105 | UNKNOWN | 0.91 | 0.71 | 0.58 | 0.04 | 1.10 | 0.76 | 1.20 | 0.49 | 1.07 | 0.80 | 2.48 | 0.01 |
| CB507120 | Hepatitis B virus X-interacting protein | 1.38 | 0.38 | 0.69 | 0.30 | 0.86 | 0.66 | 1.04 | 0.92 | 0.33 | 0.00 | 0.77 | 0.46 |
| CB511485 | UNKNOWN | 1.07 | 0.85 | 0.26 | 0.00 | 0.67 | 0.26 | 1.20 | 0.62 | 0.48 | 0.05 | 1.76 | 0.12 |
| CA041207 | UNKNOWN | 0.98 | 0.95 | 0.47 | 0.00 | 0.54 | 0.03 | 1.24 | 0.43 | 0.86 | 0.55 | 0.90 | 0.70 |
| CB496828 | envelope protein [Atlantic salmon swim bladder sarcoma | 1.25 | 0.49 | 0.32 | 0.00 | 0.71 | 0.30 | 1.68 | 0.11 | 0.65 | 0.21 | 0.99 | 0.97 |
| CB494075 | Myristoylated alanine-rich C-kinase substrate | 0.69 | 0.11 | 0.50 | 0.00 | 0.90 | 0.64 | 0.82 | 0.38 | 0.99 | 0.95 | 0.97 | 0.89 |
| CA057966 | Oncorhynchus mykiss genes, MHC class I b region, | 2.04 | 0.02 | 2.57 | 0.00 | 1.22 | 0.53 | 1.70 | 0.08 | 2.29 | 0.01 | 1.63 | 0.13 |
| CA050786 | Interleukin-2 receptor subunit beta precursor | 0.76 | 0.46 | 2.58 | 0.01 | 0.60 | 0.14 | 0.99 | 0.98 | 1.86 | 0.10 | 0.73 | 0.36 |
| CA059768 | Zinc finger protein 205 | 1.46 | 0.23 | 2.57 | 0.00 | 0.89 | 0.70 | 1.32 | 0.38 | 1.53 | 0.17 | 0.97 | 0.91 |
| CA054784 | Exocyst complex component 3 | 1.02 | 0.92 | 1.58 | 0.03 | 0.71 | 0.13 | 0.91 | 0.67 | 1.49 | 0.06 | 0.56 | 0.01 |
| CK991076 | type IV antifreeze protein precursor [Danio rerio] | 0.46 | 0.00 | 0.96 | 0.86 | 1.00 | 1.00 | 0.88 | 0.58 | 1.20 | 0.46 | 0.95 | 0.81 |
| CA058337 | NADPH--cytochrome P450 reductase | 1.37 | 0.30 | 1.55 | 0.17 | 0.77 | 0.42 | 0.93 | 0.80 | 2.27 | 0.01 | 1.07 | 0.82 |
| CB514000 | Protein-lysine 6-oxidase precursor | 0.83 | 0.40 | 2.21 | 0.00 | 0.64 | 0.05 | 0.80 | 0.32 | 1.42 | 0.12 | 0.97 | 0.90 |
| CA060271 | Proteasome subunit beta type 5 precursor | 0.79 | 0.28 | 0.93 | 0.68 | 0.62 | 0.01 | 0.88 | 0.57 | 1.23 | 0.24 | 0.99 | 0.95 |
| CA064217 | UNKNOWN | 1.39 | 0.23 | 2.12 | 0.01 | 0.87 | 0.59 | 1.19 | 0.53 | 1.65 | 0.09 | 1.07 | 0.81 |
| CA058187 | Krueppel-like factor 6 | 1.83 | 0.03 | 2.10 | 0.01 | 0.58 | 0.05 | 1.56 | 0.11 | 1.46 | 0.17 | 0.58 | 0.05 |
| CB509993 | Brain protein 44-like protein | 1.29 | 0.43 | 1.02 | 0.95 | 0.84 | 0.57 | 1.48 | 0.24 | 2.43 | 0.01 | 0.93 | 0.83 |
| CB495063 | Transmembrane emp24 domain-containing protein 4 | 1.13 | 0.64 | 1.98 | 0.01 | 0.91 | 0.72 | 0.91 | 0.72 | 1.38 | 0.22 | 1.04 | 0.88 |
| CB500149 | UNKNOWN | 1.11 | 0.69 | 2.39 | 0.00 | 0.72 | 0.26 | 0.77 | 0.34 | 1.14 | 0.62 | 0.88 | 0.67 |
| CB516952 | 14-3-3 protein epsilon | 0.51 | 0.01 | 0.63 | 0.07 | 1.04 | 0.88 | 0.70 | 0.14 | 1.26 | 0.35 | 1.19 | 0.49 |
| CA050686 | 2-oxoglutarate dehydrogenase E1 component, | 1.20 | 0.35 | 1.15 | 0.49 | 0.52 | 0.00 | 1.07 | 0.75 | 1.12 | 0.59 | 0.80 | 0.27 |
| CB493062 | Alcohol dehydrogenase class 3 | 1.46 | 0.29 | 0.97 | 0.93 | 1.54 | 0.25 | 1.23 | 0.56 | 3.11 | 0.00 | 0.81 | 0.56 |
| CA054657 | UNKNOWN | 1.55 | 0.10 | 0.90 | 0.68 | 0.46 | 0.01 | 1.34 | 0.26 | 0.93 | 0.78 | 0.69 | 0.18 |
| CA063716 | UNKNOWN | 0.45 | 0.01 | 0.71 | 0.19 | 1.00 | 0.99 | 0.71 | 0.28 | 1.14 | 0.62 | 1.30 | 0.31 |
| CB510232 | SH2 domain-containing adapter protein E | 2.66 | 0.10 | 0.27 | 0.02 | 0.87 | 0.78 | 2.39 | 0.12 | 0.23 | 0.01 | 1.02 | 0.98 |
| CB494370 | Cyclin-I | 1.63 | 0.33 | 0.61 | 0.32 | 0.79 | 0.63 | 3.63 | 0.01 | 0.55 | 0.23 | 1.09 | 0.87 |
| CK990496 | Cathepsin L precursor | 0.72 | 0.38 | 0.30 | 0.00 | 1.01 | 0.98 | 1.12 | 0.76 | 0.30 | 0.00 | 1.61 | 0.22 |
| CA043518 | Aldose reductase | 1.14 | 0.71 | 0.57 | 0.12 | 0.85 | 0.65 | 0.41 | 0.03 | 0.39 | 0.01 | 1.63 | 0.17 |
| CA063844 | Cytochrome c oxidase subunit VIIa-related protein | 2.10 | 0.11 | 0.58 | 0.18 | 0.87 | 0.73 | 2.22 | 0.11 | 0.38 | 0.01 | 0.89 | 0.77 |
| CB508332 | UNKNOWN | 2.48 | 0.10 | 0.41 | 0.04 | 1.25 | 0.61 | 5.31 | 0.00 | 0.29 | 0.01 | 0.85 | 0.70 |
| CB494193 | Interferon-induced 17 kDa protein precursor | 1.04 | 0.92 | 0.65 | 0.28 | 2.11 | 0.07 | 0.29 | 0.01 | 0.89 | 0.76 | 1.57 | 0.26 |
| CB510525 | Guanine nucleotide-binding protein G(t) subunit alpha | 2.43 | 0.12 | 0.21 | 0.01 | 1.28 | 0.67 | 4.36 | 0.01 | 0.17 | 0.00 | 1.56 | 0.44 |
| CB505648 | UNKNOWN | 1.03 | 0.93 | 0.34 | 0.01 | 1.33 | 0.46 | 0.89 | 0.78 | 0.61 | 0.24 | 1.68 | 0.19 |
| CB510503 | Oncorhynchus mykiss mRNA for type II keratin E1 (E1 | 0.45 | 0.01 | 0.60 | 0.06 | 1.03 | 0.91 | 0.93 | 0.80 | 0.94 | 0.80 | 0.93 | 0.77 |
| CB511329 | PREDICTED: Danio rerio similar to TRIP12 protein | 0.81 | 0.49 | 0.38 | 0.00 | 1.33 | 0.42 | 1.08 | 0.80 | 0.72 | 0.25 | 1.45 | 0.30 |
| CB517288 | UNKNOWN | 0.67 | 0.40 | 0.26 | 0.00 | 0.90 | 0.81 | 0.81 | 0.65 | 0.61 | 0.27 | 1.14 | 0.77 |
| CA051796 | UNKNOWN | 0.89 | 0.77 | 0.35 | 0.00 | 0.75 | 0.44 | 1.17 | 0.68 | 0.62 | 0.17 | 0.87 | 0.71 |
| CB511359 | UNKNOWN | 3.36 | 0.02 | 0.45 | 0.06 | 2.17 | 0.12 | 1.38 | 0.53 | 0.27 | 0.00 | 2.43 | 0.07 |
| CA058492 | novel protein [Xenopus tropicalis] | 1.15 | 0.88 | 0.17 | 0.00 | 1.39 | 0.57 | 1.63 | 0.49 | 0.22 | 0.02 | 1.80 | 0.31 |
| CB496738 | mRNA-binding protein expressed during iron starvation | 3.80 | 0.01 | 1.15 | 0.76 | 1.68 | 0.28 | 1.80 | 0.20 | 0.63 | 0.34 | 2.20 | 0.10 |
| CA043922 | Salmo salar ppar gamma gene for peroxisome proliferator- | 1.41 | 0.32 | 0.44 | 0.01 | 0.90 | 0.74 | 1.80 | 0.09 | 0.55 | 0.05 | 1.18 | 0.59 |
| CB498135 | Actin, alpha skeletal muscle | 0.52 | 0.04 | 0.82 | 0.49 | 2.35 | 0.01 | 0.84 | 0.57 | 1.29 | 0.38 | 0.98 | 0.96 |
| CB515951 | GrpE protein homolog 1, mitochondrial precursor | 1.78 | 0.29 | 0.42 | 0.09 | 0.87 | 0.78 | 0.60 | 0.40 | 0.24 | 0.01 | 1.47 | 0.43 |
| CA056895 | PREDICTED: similar to C6orf49 protein | 0.51 | 0.01 | 1.01 | 0.97 | 1.03 | 0.90 | 0.59 | 0.05 | 1.27 | 0.28 | 1.24 | 0.31 |
| CB513818 | Oncorhynchus mykiss SYPG1 (SYPG1), PHF1 (PHF1), | 0.86 | 0.59 | 0.50 | 0.01 | 0.88 | 0.64 | 1.03 | 0.92 | 0.89 | 0.67 | 1.18 | 0.54 |
| CA055445 | UNKNOWN | 2.18 | 0.07 | 0.67 | 0.29 | 1.17 | 0.67 | 0.95 | 0.90 | 0.38 | 0.01 | 1.72 | 0.15 |
| CB515373 | Heat shock factor protein 2 | 1.38 | 0.41 | 0.45 | 0.05 | 1.26 | 0.59 | 1.91 | 0.11 | 0.34 | 0.01 | 1.00 | 1.00 |
| CA062114 | Elastase-1 | 3.19 | 0.01 | 0.66 | 0.33 | 0.78 | 0.61 | 2.25 | 0.07 | 0.74 | 0.48 | 0.74 | 0.53 |
| CK990305 | Protein RCC2 homolog | 1.40 | 0.41 | 1.14 | 0.78 | 1.15 | 0.76 | 0.71 | 0.40 | 3.86 | 0.01 | 1.76 | 0.22 |
| CA062473 | FYN-binding protein | 1.29 | 0.38 | 2.34 | 0.00 | 0.58 | 0.06 | 1.33 | 0.32 | 1.84 | 0.04 | 1.25 | 0.43 |
| DN047593 | Tumor necrosis factor, alpha-induced protein 8-like | 1.21 | 0.62 | 3.30 | 0.01 | 1.07 | 0.85 | 1.25 | 0.56 | 0.81 | 0.62 | 0.91 | 0.80 |
| CB492157 | UNKNOWN | 1.94 | 0.05 | 2.82 | 0.00 | 0.88 | 0.69 | 1.49 | 0.25 | 1.32 | 0.41 | 0.87 | 0.68 |
| CA056114 | Protein pob | 3.27 | 0.01 | 0.86 | 0.77 | 0.89 | 0.78 | 2.06 | 0.11 | 0.50 | 0.17 | 0.99 | 0.98 |
| CA051087 | Grancalcin | 1.88 | 0.01 | 1.09 | 0.71 | 0.71 | 0.11 | 0.95 | 0.83 | 1.41 | 0.13 | 0.80 | 0.27 |
| CB515410 | Uncharacterized protein C10orf84 homolog | 3.16 | 0.01 | 0.97 | 0.94 | 0.97 | 0.94 | 1.80 | 0.15 | 1.01 | 0.99 | 1.33 | 0.51 |
| CA048694 | Tripartite motif-containing protein 29 | 2.51 | 0.01 | 1.49 | 0.20 | 0.85 | 0.61 | 2.81 | 0.00 | 1.23 | 0.50 | 0.74 | 0.32 |
| CA061219 | Nonspecific lipid-transfer protein | 1.12 | 0.71 | 1.09 | 0.79 | 1.51 | 0.19 | 2.22 | 0.01 | 1.31 | 0.39 | 1.02 | 0.96 |
| CA059717 | Histone H2AV | 0.74 | 0.20 | 0.54 | 0.01 | 0.90 | 0.61 | 1.03 | 0.89 | 0.82 | 0.39 | 0.91 | 0.65 |
| CA058558 | COP9 signalosome complex subunit 3 | 1.35 | 0.20 | 1.73 | 0.01 | 0.74 | 0.14 | 1.24 | 0.35 | 1.23 | 0.28 | 0.78 | 0.24 |
| CB493974 | 26S proteasome non-ATPase regulatory subunit 11 | 2.46 | 0.00 | 1.04 | 0.86 | 0.62 | 0.04 | 1.70 | 0.04 | 1.65 | 0.02 | 0.84 | 0.44 |
| CA049909 | Developmentally-regulated RNA-binding protein 1 | 1.58 | 0.11 | 3.74 | 0.00 | 0.85 | 0.56 | 1.43 | 0.21 | 1.82 | 0.06 | 0.89 | 0.68 |
| CA050491 | 26S proteasome non-ATPase regulatory subunit 11 | 1.10 | 0.67 | 1.96 | 0.00 | 1.17 | 0.51 | 1.40 | 0.15 | 1.51 | 0.07 | 0.86 | 0.53 |
| CA038875 | Induced myeloid leukemia cell differentiation protein Mcl- | 2.30 | 0.00 | 0.88 | 0.60 | 0.93 | 0.80 | 1.46 | 0.12 | 0.98 | 0.95 | 0.81 | 0.44 |
| CB494235 | Actin-related protein 2/3 complex subunit 1A | 1.67 | 0.03 | 1.97 | 0.01 | 0.78 | 0.30 | 1.60 | 0.05 | 1.67 | 0.03 | 0.86 | 0.51 |
| CA058199 | Dynactin subunit 3 | 0.95 | 0.72 | 1.40 | 0.01 | 0.94 | 0.66 | 0.91 | 0.53 | 1.28 | 0.05 | 0.91 | 0.48 |
| CB501290 | Transposable element Tcb2 transposase | 0.61 | 0.06 | 0.52 | 0.01 | 0.62 | 0.06 | 0.84 | 0.49 | 1.23 | 0.40 | 1.01 | 0.98 |
| CA052316 | UNKNOWN | 1.03 | 0.91 | 0.45 | 0.01 | 0.68 | 0.16 | 1.15 | 0.62 | 0.83 | 0.54 | 1.05 | 0.87 |
| CA058803 | Spindlin-1 | 1.21 | 0.44 | 1.82 | 0.01 | 0.82 | 0.36 | 1.57 | 0.07 | 1.45 | 0.08 | 0.70 | 0.11 |
| CB497486 | UNKNOWN | 1.19 | 0.57 | 0.45 | 0.01 | 0.87 | 0.62 | 1.21 | 0.53 | 0.84 | 0.53 | 1.50 | 0.15 |
| CA050905 | diaphorase (NADH) (cytochrome b-5 reductase) | 0.61 | 0.06 | 2.98 | 0.00 | 0.77 | 0.28 | 0.89 | 0.62 | 1.33 | 0.37 | 0.83 | 0.44 |
| CB497161 | Oncorhynchus mykiss clone B21 VHSV-induced mRNA, | 0.77 | 0.41 | 0.44 | 0.01 | 1.02 | 0.95 | 1.11 | 0.76 | 0.78 | 0.40 | 1.33 | 0.42 |
| CA060163 | Ribosomal protein S6 kinase beta-2 | 1.37 | 0.36 | 0.37 | 0.00 | 0.96 | 0.91 | 0.88 | 0.70 | 0.54 | 0.06 | 1.43 | 0.29 |
| CA056618 | Aminopeptidase B | 0.90 | 0.74 | 2.29 | 0.01 | 1.11 | 0.75 | 0.65 | 0.17 | 1.58 | 0.12 | 0.94 | 0.86 |
| CA050681 | UNKNOWN | 0.34 | 0.01 | 0.58 | 0.09 | 1.27 | 0.50 | 0.41 | 0.03 | 0.86 | 0.63 | 1.40 | 0.34 |
| CA047947 | Takifugu rubripes protocadherin gene locus 2, complete | 1.22 | 0.47 | 2.02 | 0.01 | 0.88 | 0.65 | 0.76 | 0.32 | 1.27 | 0.39 | 0.96 | 0.88 |
| CA059883 | Protein Tob1 | 0.89 | 0.59 | 1.95 | 0.00 | 0.76 | 0.18 | 0.73 | 0.13 | 1.01 | 0.95 | 0.80 | 0.29 |
| CB511864 | Monocyte to macrophage differentiation protein | 0.89 | 0.73 | 3.59 | 0.00 | 1.17 | 0.64 | 1.07 | 0.84 | 1.03 | 0.93 | 0.87 | 0.69 |
| CA057580 | Xanthine dehydrogenase/oxidase | 1.34 | 0.43 | 0.33 | 0.00 | 0.98 | 0.96 | 0.88 | 0.73 | 0.41 | 0.02 | 1.03 | 0.93 |
| CA064496 | RNA 3'-terminal phosphate cyclase | 1.25 | 0.32 | 2.54 | 0.00 | 1.06 | 0.80 | 0.93 | 0.77 | 1.47 | 0.11 | 0.92 | 0.69 |
| CA038180 | NADH dehydrogenase [ubiquinone] 1 alpha subcomplex | 0.48 | 0.01 | 0.79 | 0.41 | 0.86 | 0.62 | 0.41 | 0.00 | 1.03 | 0.92 | 1.66 | 0.11 |
| CA051166 | UNKNOWN | 1.06 | 0.88 | 0.65 | 0.30 | 2.23 | 0.07 | 2.26 | 0.06 | 0.95 | 0.90 | 3.16 | 0.01 |
| CK990503 | UNKNOWN | 1.39 | 0.42 | 0.56 | 0.12 | 1.40 | 0.37 | 0.37 | 0.01 | 0.99 | 0.98 | 1.38 | 0.39 |
| CA050773 | UNKNOWN | 1.04 | 0.89 | 0.43 | 0.01 | 0.95 | 0.85 | 1.31 | 0.35 | 0.88 | 0.70 | 1.11 | 0.72 |
| CB497603 | Acyl-CoA-binding protein | 1.94 | 0.01 | 1.92 | 0.01 | 0.97 | 0.88 | 1.14 | 0.58 | 1.16 | 0.55 | 1.04 | 0.85 |
| CB512197 | Oncorhynchus mykiss SYPG1 (SYPG1), PHF1 (PHF1) | 2.16 | 0.01 | 0.97 | 0.91 | 0.97 | 0.91 | 1.72 | 0.04 | 0.96 | 0.86 | 1.32 | 0.31 |
| CA044503 | Small inducible cytokine B14 precursor | 13.59 | 0.00 | 0.99 | 0.99 | 1.54 | 0.57 | 1.68 | 0.51 | 0.93 | 0.92 | 0.94 | 0.93 |
| CK990484 | UNKNOWN | 0.95 | 0.86 | 0.46 | 0.01 | 0.81 | 0.45 | 1.12 | 0.70 | 0.64 | 0.13 | 1.22 | 0.46 |
| CB502615 | Phospholipase AdRab-B precursor | 1.43 | 0.30 | 0.30 | 0.00 | 0.94 | 0.86 | 1.10 | 0.79 | 0.72 | 0.36 | 1.64 | 0.15 |
| CB497660 | Superoxide dismutase [Mn], mitochondrial precursor | 0.97 | 0.94 | 0.42 | 0.01 | 0.98 | 0.96 | 0.84 | 0.61 | 0.64 | 0.19 | 1.33 | 0.40 |
| CA037123 | NADH dehydrogenase [ubiquinone] iron-sulfur protein 3, | 2.93 | 0.00 | 0.34 | 0.01 | 0.73 | 0.37 | 3.30 | 0.00 | 0.45 | 0.04 | 1.14 | 0.71 |
| CA059209 | Ependymin precursor | 1.01 | 0.97 | 3.39 | 0.00 | 0.69 | 0.35 | 0.80 | 0.55 | 1.61 | 0.24 | 0.95 | 0.89 |
| CA038163 | Complement C3-1 | 4.34 | 0.00 | 0.62 | 0.24 | 1.13 | 0.78 | 1.99 | 0.13 | 0.93 | 0.86 | 1.39 | 0.45 |
| CB515166 | Oncorhynchus mykiss C5a receptor (C5aR) gene, complete | 0.79 | 0.47 | 0.43 | 0.01 | 1.01 | 0.98 | 1.45 | 0.25 | 0.70 | 0.25 | 1.35 | 0.37 |
| CB492414 | T-complex protein 1 subunit delta | 0.57 | 0.15 | 0.35 | 0.01 | 0.90 | 0.79 | 0.58 | 0.16 | 0.71 | 0.40 | 1.17 | 0.67 |
| CA037175 | UNKNOWN | 1.04 | 0.93 | 1.76 | 0.29 | 0.75 | 0.63 | 4.69 | 0.00 | 1.14 | 0.81 | 0.54 | 0.31 |
| CA058709 | UNKNOWN | 1.14 | 0.69 | 0.39 | 0.00 | 1.07 | 0.84 | 1.49 | 0.21 | 0.85 | 0.60 | 1.58 | 0.20 |
| CA770402 | 60S ribosomal protein L15 | 1.05 | 0.89 | 0.31 | 0.01 | 0.95 | 0.91 | 1.04 | 0.92 | 0.69 | 0.40 | 0.96 | 0.92 |
| CB493442 | Sorcin | 6.40 | 0.00 | 1.31 | 0.62 | 0.65 | 0.42 | 3.40 | 0.04 | 1.14 | 0.80 | 0.75 | 0.59 |
| CA063127 | Armadillo repeat-containing protein 6 | 1.48 | 0.14 | 1.10 | 0.71 | 1.49 | 0.15 | 1.03 | 0.90 | 0.97 | 0.90 | 0.49 | 0.01 |
| CB499194 | 5-aminolevulinate synthase, nonspecific, mitochondrial | 2.60 | 0.01 | 0.99 | 0.98 | 0.75 | 0.39 | 1.77 | 0.12 | 0.86 | 0.65 | 1.03 | 0.93 |
| CA061170 | Mitogen-activated protein-binding protein-interacting | 0.67 | 0.05 | 2.29 | 0.00 | 0.87 | 0.48 | 1.01 | 0.95 | 1.03 | 0.88 | 0.97 | 0.88 |
| CB507979 | UNKNOWN | 1.27 | 0.28 | 0.73 | 0.15 | 0.92 | 0.68 | 1.53 | 0.06 | 0.51 | 0.00 | 1.54 | 0.05 |
| CA045072 | Staphylococcal nuclease domain-containing protein 1 | 0.60 | 0.31 | 0.46 | 0.08 | 1.66 | 0.24 | 0.22 | 0.00 | 1.14 | 0.75 | 1.01 | 0.99 |
| CA046853 | UNKNOWN | 0.72 | 0.28 | 0.42 | 0.00 | 0.74 | 0.28 | 0.76 | 0.37 | 0.70 | 0.19 | 0.86 | 0.59 |
| CA057288 | Brix domain-containing protein 1 | 1.15 | 0.72 | 1.30 | 0.53 | 1.02 | 0.96 | 1.15 | 0.72 | 3.01 | 0.01 | 1.50 | 0.36 |
| CB510581 | UNKNOWN | 1.12 | 0.82 | 0.70 | 0.49 | 1.78 | 0.23 | 4.25 | 0.01 | 0.93 | 0.88 | 0.77 | 0.58 |
| CA038906 | Haptoglobin precursor | 0.84 | 0.49 | 0.40 | 0.00 | 0.81 | 0.40 | 0.79 | 0.35 | 0.90 | 0.68 | 1.19 | 0.49 |
| CB508690 | HEAT repeat-containing protein 3 | 1.28 | 0.30 | 1.69 | 0.04 | 0.74 | 0.17 | 0.90 | 0.66 | 2.53 | 0.00 | 1.00 | 0.98 |
| CB511019 | Salmo salar zonadhesin-like gene, complete cds and 3' | 0.73 | 0.21 | 0.40 | 0.00 | 0.86 | 0.45 | 1.21 | 0.45 | 0.83 | 0.37 | 1.43 | 0.09 |
| CB501013 | Hemoglobin subunit beta | 0.83 | 0.43 | 0.54 | 0.01 | 0.70 | 0.22 | 1.21 | 0.43 | 0.72 | 0.13 | 0.96 | 0.88 |
| CA052736 | Vacuolar ATP synthase subunit D | 0.96 | 0.82 | 2.12 | 0.00 | 0.81 | 0.31 | 0.73 | 0.14 | 1.60 | 0.03 | 0.91 | 0.65 |
| CB488187 | Translocator protein | 1.22 | 0.41 | 1.22 | 0.37 | 0.91 | 0.67 | 0.62 | 0.06 | 2.08 | 0.00 | 1.17 | 0.49 |
| CA045023 | Basigin precursor | 0.98 | 0.95 | 2.29 | 0.00 | 0.70 | 0.17 | 0.83 | 0.45 | 1.54 | 0.07 | 0.81 | 0.41 |
| CA056748 | Rattus norvegicus actin, alpha 1, skeletal muscle (Acta1) | 0.47 | 0.00 | 0.74 | 0.23 | 1.10 | 0.74 | 0.82 | 0.43 | 1.28 | 0.31 | 1.17 | 0.57 |
| CA042944 | Apolipoprotein B-100 precursor | 0.80 | 0.19 | 1.58 | 0.01 | 0.68 | 0.16 | 1.35 | 0.08 | 1.32 | 0.10 | 0.46 | 0.01 |
| CA052937 | UNKNOWN | 1.52 | 0.12 | 1.82 | 0.02 | 1.01 | 0.98 | 1.39 | 0.22 | 2.05 | 0.01 | 0.84 | 0.53 |
| CA046950 | UNKNOWN | 1.08 | 0.75 | 2.73 | 0.00 | 0.79 | 0.34 | 0.72 | 0.20 | 1.73 | 0.03 | 1.11 | 0.68 |
| CA057203 | ADP/ATP translocase 2 | 0.63 | 0.10 | 0.97 | 0.90 | 0.85 | 0.51 | 0.95 | 0.86 | 1.88 | 0.01 | 1.07 | 0.79 |
| CA054244 | Hematological and neurological expressed 1-like protein | 0.83 | 0.45 | 0.89 | 0.58 | 1.09 | 0.76 | 1.12 | 0.63 | 1.79 | 0.01 | 1.58 | 0.10 |
| CB508909 | 26S proteasome non-ATPase regulatory subunit 9 | 1.09 | 0.73 | 1.57 | 0.08 | 0.82 | 0.44 | 1.14 | 0.61 | 1.99 | 0.01 | 0.78 | 0.32 |
| CB512550 | Peroxiredoxin-5, mitochondrial precursor | 1.55 | 0.17 | 1.66 | 0.13 | 0.69 | 0.24 | 0.72 | 0.29 | 2.58 | 0.01 | 0.95 | 0.87 |
| CB510383 | Beta crystallin S | 0.28 | 0.02 | 1.50 | 0.47 | 1.24 | 0.68 | 0.26 | 0.01 | 0.99 | 0.98 | 0.84 | 0.74 |
| CB492794 | HD domain-containing protein 3 | 1.30 | 0.39 | 1.32 | 0.31 | 0.76 | 0.36 | 1.05 | 0.86 | 2.34 | 0.00 | 0.80 | 0.45 |
| CB512292 | Cytochrome c oxidase subunit VIb isoform 1 | 1.15 | 0.45 | 1.64 | 0.01 | 0.84 | 0.37 | 1.07 | 0.71 | 1.37 | 0.09 | 0.94 | 0.75 |
| CK990458 | Pre-mRNA branch site protein p14 | 0.95 | 0.84 | 2.06 | 0.00 | 0.83 | 0.46 | 0.72 | 0.21 | 1.63 | 0.03 | 0.93 | 0.78 |
| CB492334 | UNKNOWN | 1.20 | 0.41 | 1.92 | 0.01 | 0.69 | 0.08 | 0.65 | 0.06 | 1.51 | 0.07 | 1.26 | 0.27 |
| CB499379 | TGF-beta receptor type-1 precursor | 1.66 | 0.04 | 2.56 | 0.00 | 0.72 | 0.19 | 1.21 | 0.43 | 1.60 | 0.07 | 0.87 | 0.59 |
| CB494406 | Fidgetin | 1.17 | 0.57 | 1.91 | 0.03 | 0.84 | 0.55 | 0.98 | 0.94 | 2.26 | 0.01 | 0.92 | 0.78 |
| CA056954 | UNKNOWN | 1.45 | 0.25 | 2.36 | 0.01 | 1.37 | 0.36 | 0.92 | 0.80 | 2.30 | 0.01 | 1.29 | 0.46 |
| CA058532 | UNKNOWN | 0.90 | 0.79 | 0.34 | 0.01 | 0.57 | 0.12 | 1.36 | 0.44 | 0.71 | 0.39 | 0.96 | 0.90 |
| CA052877 | Glutathione peroxidase 3 precursor | 0.89 | 0.67 | 2.05 | 0.01 | 0.80 | 0.42 | 0.97 | 0.92 | 1.28 | 0.35 | 0.99 | 0.99 |
| CA055640 | UNKNOWN | 1.45 | 0.34 | 2.56 | 0.01 | 0.94 | 0.86 | 1.56 | 0.26 | 1.80 | 0.09 | 0.90 | 0.76 |
| CA044615 | Alcohol dehydrogenase class 3 | 0.48 | 0.01 | 0.82 | 0.46 | 0.80 | 0.43 | 0.74 | 0.29 | 1.65 | 0.07 | 0.79 | 0.39 |
| CA063413 | Mitochondrial carrier homolog 2 | 0.96 | 0.84 | 1.46 | 0.06 | 0.91 | 0.58 | 0.65 | 0.02 | 1.64 | 0.01 | 1.02 | 0.92 |
| CB511643 | Lysozyme g | 1.04 | 0.91 | 2.41 | 0.01 | 0.84 | 0.62 | 1.13 | 0.72 | 1.40 | 0.32 | 1.03 | 0.94 |
| CA046166 | Baculoviral IAP repeat-containing protein 6 | 1.93 | 0.02 | 1.98 | 0.02 | 0.88 | 0.64 | 1.10 | 0.74 | 2.21 | 0.01 | 1.06 | 0.82 |
| CA037364 | UNKNOWN | 1.07 | 0.72 | 0.80 | 0.21 | 0.60 | 0.01 | 1.15 | 0.44 | 1.04 | 0.82 | 0.85 | 0.39 |
| DN047544 | Cell division cycle protein 20 homolog | 1.03 | 0.87 | 1.72 | 0.01 | 0.77 | 0.19 | 1.11 | 0.60 | 1.57 | 0.02 | 0.98 | 0.92 |
| CA037902 | UNKNOWN | 1.16 | 0.67 | 1.73 | 0.07 | 1.40 | 0.26 | 0.51 | 0.06 | 2.18 | 0.01 | 0.89 | 0.69 |
| CB493105 | UNKNOWN | 1.05 | 0.86 | 2.48 | 0.00 | 0.60 | 0.06 | 0.75 | 0.25 | 2.16 | 0.00 | 0.61 | 0.06 |
| CA057213 | Aldehyde dehydrogenase, mitochondrial precursor | 3.23 | 0.00 | 1.45 | 0.29 | 1.08 | 0.84 | 2.17 | 0.05 | 2.04 | 0.05 | 0.64 | 0.24 |
| CA054749 | Mitochondrial 28S ribosomal protein S30 | 0.91 | 0.65 | 1.21 | 0.39 | 0.38 | 0.00 | 1.00 | 0.98 | 1.57 | 0.05 | 0.74 | 0.22 |
| CB497287 | Cytochrome c oxidase subunit VIIa-related protein | 1.25 | 0.22 | 1.79 | 0.00 | 0.76 | 0.13 | 0.93 | 0.69 | 1.58 | 0.01 | 0.74 | 0.09 |
| CB496523 | 15-hydroxyprostaglandin dehydrogenase [NAD+] | 1.40 | 0.52 | 4.66 | 0.00 | 0.72 | 0.55 | 1.03 | 0.96 | 1.83 | 0.23 | 0.69 | 0.50 |
| CA046402 | Ras-related protein Rab-11B | 1.04 | 0.89 | 2.29 | 0.01 | 0.86 | 0.58 | 1.03 | 0.91 | 1.44 | 0.26 | 0.92 | 0.77 |
| CA053858 | UNKNOWN | 0.82 | 0.48 | 3.06 | 0.00 | 0.70 | 0.21 | 0.98 | 0.94 | 1.59 | 0.13 | 0.90 | 0.70 |
| CA058656 | Adaptin ear-binding coat-associated protein 2 | 1.36 | 0.35 | 2.33 | 0.01 | 1.13 | 0.70 | 1.39 | 0.31 | 2.38 | 0.01 | 1.23 | 0.50 |
| CB516664 | Malate dehydrogenase, cytoplasmic | 1.33 | 0.18 | 1.51 | 0.06 | 0.86 | 0.45 | 0.90 | 0.62 | 1.80 | 0.01 | 0.86 | 0.45 |
| CB514264 | DCN1-like protein 1 | 1.53 | 0.19 | 1.83 | 0.06 | 1.22 | 0.52 | 1.39 | 0.31 | 2.23 | 0.01 | 1.40 | 0.28 |
| DN048059 | UNKNOWN | 1.07 | 0.78 | 1.89 | 0.01 | 0.85 | 0.45 | 1.10 | 0.69 | 1.45 | 0.09 | 0.97 | 0.90 |
| CA060056 | Ornithine decarboxylase antizyme 2 | 1.45 | 0.47 | 1.13 | 0.80 | 0.95 | 0.91 | 4.78 | 0.00 | 1.80 | 0.24 | 1.12 | 0.81 |
| CA039064 | UNKNOWN | 2.59 | 0.01 | 1.38 | 0.25 | 0.63 | 0.11 | 2.29 | 0.02 | 1.77 | 0.04 | 1.40 | 0.22 |
| CA040414 | Apolipoprotein Eb precursor | 1.34 | 0.33 | 2.81 | 0.00 | 1.06 | 0.84 | 1.40 | 0.27 | 1.82 | 0.04 | 1.01 | 0.97 |
| CA052080 | NF-kappa-B inhibitor-interacting Ras-like protein 2 | 1.01 | 0.97 | 1.81 | 0.01 | 0.83 | 0.38 | 1.03 | 0.88 | 1.74 | 0.01 | 0.84 | 0.39 |
| CA041428 | UNKNOWN | 1.90 | 0.01 | 1.99 | 0.00 | 0.90 | 0.67 | 1.26 | 0.32 | 1.68 | 0.03 | 0.85 | 0.49 |
| CB509812 | UNKNOWN | 0.87 | 0.73 | 1.43 | 0.39 | 0.78 | 0.52 | 1.52 | 0.29 | 3.37 | 0.01 | 0.75 | 0.46 |
| CB517506 | Interleukin-13 receptor alpha-2 chain precursor | 2.10 | 0.03 | 3.43 | 0.00 | 1.07 | 0.85 | 1.31 | 0.41 | 2.22 | 0.02 | 1.05 | 0.88 |
| CB493923 | UNKNOWN | 1.30 | 0.29 | 1.27 | 0.31 | 1.06 | 0.80 | 1.10 | 0.69 | 1.84 | 0.01 | 0.93 | 0.74 |
| CA057718 | UNKNOWN | 1.51 | 0.14 | 2.13 | 0.01 | 0.78 | 0.38 | 1.43 | 0.20 | 1.79 | 0.05 | 0.70 | 0.21 |
| CA063549 | Apolipoprotein F precursor | 1.42 | 0.55 | 0.92 | 0.88 | 1.02 | 0.97 | 10.15 | 0.00 | 0.63 | 0.37 | 1.09 | 0.88 |
| CA051597 | Proliferation-associated protein 2G4 | 1.19 | 0.35 | 1.01 | 0.97 | 0.93 | 0.73 | 1.05 | 0.78 | 1.82 | 0.00 | 0.81 | 0.31 |
| CA047240 | UNKNOWN | 2.68 | 0.01 | 2.46 | 0.00 | 1.46 | 0.28 | 1.30 | 0.42 | 1.94 | 0.03 | 1.27 | 0.49 |
| CA062036 | UNKNOWN | 0.38 | 0.01 | 0.60 | 0.15 | 0.79 | 0.49 | 0.72 | 0.36 | 0.96 | 0.90 | 1.15 | 0.69 |
| CA040492 | Ig mu chain C region membrane-bound form | 2.48 | 0.01 | 1.47 | 0.30 | 0.79 | 0.50 | 1.04 | 0.92 | 1.40 | 0.32 | 1.38 | 0.35 |
| CK990341 | UNKNOWN | 1.55 | 0.04 | 2.21 | 0.00 | 0.98 | 0.92 | 1.47 | 0.07 | 1.25 | 0.26 | 0.92 | 0.72 |
| CB516482 | FACT complex subunit SSRP1 | 2.79 | 0.01 | 1.29 | 0.54 | 0.71 | 0.38 | 1.29 | 0.52 | 2.29 | 0.05 | 1.02 | 0.96 |
| CB487670 | Proteasome activator complex subunit 3 | 0.83 | 0.47 | 2.89 | 0.00 | 0.76 | 0.29 | 0.58 | 0.04 | 1.65 | 0.04 | 1.01 | 0.98 |
| BU965768 | NADH dehydrogenase [ubiquinone] 1 alpha subcomplex | 1.19 | 0.63 | 0.81 | 0.52 | 0.92 | 0.82 | 0.86 | 0.68 | 2.35 | 0.01 | 0.80 | 0.54 |
| CA767615 | Cytoplasmic FMR1-interacting protein 1 homolog | 1.14 | 0.77 | 2.01 | 0.12 | 0.90 | 0.80 | 0.55 | 0.17 | 3.30 | 0.01 | 1.26 | 0.57 |
| CB502337 | Coronin-1A | 2.33 | 0.01 | 1.01 | 0.97 | 1.04 | 0.92 | 1.40 | 0.30 | 1.64 | 0.10 | 0.66 | 0.23 |
| CA062360 | OCIAD1 protein [Bos taurus] | 1.30 | 0.20 | 1.76 | 0.01 | 0.74 | 0.12 | 1.08 | 0.72 | 1.28 | 0.21 | 0.78 | 0.20 |
| CB509753 | UNKNOWN | 1.04 | 0.84 | 1.57 | 0.03 | 0.74 | 0.15 | 1.25 | 0.27 | 2.04 | 0.00 | 0.84 | 0.38 |
| CB498685 | N-acetyltransferase 5 | 2.23 | 0.00 | 1.65 | 0.06 | 1.14 | 0.60 | 1.13 | 0.61 | 1.02 | 0.95 | 0.87 | 0.58 |
| CB498304 | Mitochondrial inner membrane protein | 0.94 | 0.79 | 2.16 | 0.01 | 0.83 | 0.51 | 0.91 | 0.71 | 1.86 | 0.02 | 0.79 | 0.40 |
| CA052785 | G protein-regulated inducer of neurite outgrowth 2 | 1.73 | 0.14 | 3.68 | 0.00 | 0.81 | 0.56 | 2.11 | 0.06 | 1.14 | 0.74 | 0.87 | 0.71 |
| CA062062 | Hyccin | 2.81 | 0.02 | 0.78 | 0.53 | 0.99 | 0.97 | 3.02 | 0.01 | 0.71 | 0.40 | 0.86 | 0.71 |
| CB490042 | Tubulin-specific chaperone A | 1.74 | 0.06 | 2.32 | 0.01 | 1.03 | 0.93 | 1.26 | 0.42 | 1.36 | 0.30 | 1.53 | 0.13 |
| CA048889 | Ras-related protein Rab-37 | 1.29 | 0.61 | 3.07 | 0.01 | 1.04 | 0.92 | 1.07 | 0.89 | 2.07 | 0.08 | 0.76 | 0.49 |
| CB512132 | Probable G-protein coupled receptor 3 | 3.45 | 0.01 | 1.29 | 0.59 | 0.81 | 0.62 | 2.05 | 0.16 | 1.14 | 0.78 | 1.41 | 0.43 |
| CK991151 | Transcription factor HES-1 | 0.36 | 0.10 | 2.65 | 0.08 | 0.75 | 0.60 | 0.98 | 0.98 | 4.94 | 0.01 | 0.28 | 0.04 |
| CA053081 | General negative regulator of transcription subunit 1 | 0.82 | 0.52 | 1.12 | 0.74 | 1.13 | 0.70 | 0.98 | 0.94 | 1.50 | 0.22 | 2.45 | 0.01 |
| CA051876 | 60S ribosomal protein L19 | 0.17 | 0.01 | 0.20 | 0.04 | 0.69 | 0.61 | 0.22 | 0.03 | 0.24 | 0.06 | 0.62 | 0.50 |
| CA057496 | U3 small nucleolar RNA-interacting protein 2 | 0.60 | 0.04 | 1.30 | 0.32 | 0.99 | 0.95 | 0.58 | 0.03 | 2.59 | 0.00 | 0.87 | 0.51 |
| CK991257 | DNA-directed RNA polymerases I, II, and III subunit | 0.92 | 0.79 | 0.44 | 0.01 | 1.14 | 0.66 | 1.15 | 0.64 | 0.87 | 0.67 | 0.68 | 0.20 |
| CA062809 | Polyadenylate-binding protein 4 | 0.60 | 0.07 | 0.53 | 0.01 | 0.64 | 0.11 | 0.73 | 0.25 | 1.65 | 0.05 | 0.62 | 0.08 |
| CB509706 | Parvalbumin-2 | 5.97 | 0.01 | 0.25 | 0.02 | 0.77 | 0.68 | 0.98 | 0.98 | 1.47 | 0.51 | 0.93 | 0.91 |
| CA041318 | Vacuolar ATP synthase subunit G 1 | 1.16 | 0.69 | 0.39 | 0.01 | 0.79 | 0.47 | 0.66 | 0.27 | 0.72 | 0.33 | 1.54 | 0.20 |
| CK990668 | Dehydrogenase/reductase SDR family member 4 | 1.46 | 0.18 | 1.94 | 0.01 | 0.70 | 0.12 | 1.04 | 0.88 | 2.24 | 0.00 | 0.85 | 0.48 |
| CA054316 | Cullin-associated NEDD8-dissociated protein 1 | 1.14 | 0.64 | 1.90 | 0.01 | 0.99 | 0.96 | 0.70 | 0.20 | 1.65 | 0.04 | 1.09 | 0.74 |
| CB494343 | Adenosylhomocysteinase B | 1.03 | 0.95 | 0.21 | 0.01 | 0.51 | 0.19 | 2.04 | 0.21 | 0.85 | 0.77 | 1.05 | 0.92 |
| CB503498 | Creatine kinase, testis isozyme | 0.48 | 0.01 | 0.78 | 0.37 | 0.94 | 0.83 | 0.76 | 0.32 | 1.72 | 0.06 | 1.05 | 0.87 |
| CB511669 | Protein BCCIP homolog | 0.53 | 0.21 | 0.87 | 0.78 | 0.92 | 0.89 | 0.23 | 0.01 | 1.22 | 0.69 | 0.57 | 0.39 |
| CB496453 | Gap junction beta-4 protein | 2.04 | 0.23 | 1.43 | 0.50 | 0.92 | 0.88 | 6.27 | 0.01 | 0.55 | 0.27 | 0.61 | 0.40 |
| CA044387 | Salmo salar MHC class I (UBA) mRNA, UBA*1401 allele | 1.48 | 0.30 | 3.02 | 0.00 | 0.96 | 0.91 | 1.16 | 0.70 | 1.08 | 0.83 | 1.12 | 0.72 |
| CA063727 | Mitochondrial carnitine/acylcarnitine carrier protein | 1.57 | 0.33 | 0.24 | 0.00 | 0.59 | 0.23 | 1.77 | 0.25 | 0.69 | 0.40 | 0.72 | 0.47 |
| CA046770 | UNKNOWN | 1.11 | 0.72 | 2.06 | 0.01 | 1.19 | 0.55 | 1.28 | 0.41 | 1.42 | 0.20 | 0.98 | 0.93 |
| CA044652 | UNKNOWN | 0.58 | 0.23 | 0.28 | 0.01 | 0.92 | 0.86 | 0.71 | 0.44 | 0.63 | 0.33 | 1.66 | 0.28 |
| CA044594 | UNKNOWN | 1.83 | 0.40 | 1.04 | 0.95 | 1.32 | 0.66 | 4.69 | 0.01 | 1.33 | 0.62 | 0.86 | 0.81 |
| CA039920 | PREDICTED: similar to A-kinase anchoring protein | 0.84 | 0.46 | 0.67 | 0.06 | 0.51 | 0.01 | 1.17 | 0.49 | 0.83 | 0.38 | 0.62 | 0.04 |
| CA060511 | UNKNOWN | 1.34 | 0.44 | 1.32 | 0.45 | 0.75 | 0.62 | 1.04 | 0.91 | 1.14 | 0.72 | 3.16 | 0.01 |
| CK991135 | 3-ketoacyl-CoA thiolase, mitochondrial | 1.40 | 0.31 | 1.49 | 0.18 | 0.44 | 0.01 | 1.35 | 0.36 | 1.67 | 0.09 | 0.52 | 0.03 |
| CB512165 | UNKNOWN | 0.86 | 0.67 | 2.79 | 0.00 | 0.69 | 0.27 | 1.10 | 0.80 | 1.29 | 0.44 | 0.73 | 0.34 |
| CA047629 | Retinal pigment epithelium-specific 65 kDa protein | 0.84 | 0.51 | 0.89 | 0.70 | 0.48 | 0.01 | 1.39 | 0.21 | 0.73 | 0.30 | 0.69 | 0.18 |
| CB488623 | Fatty acid-binding protein 1, liver | 1.05 | 0.91 | 2.47 | 0.08 | 2.94 | 0.01 | 0.68 | 0.39 | 2.14 | 0.13 | 3.51 | 0.00 |
| CA053442 | Medium-chain specific acyl-CoA dehydrogenase | 0.92 | 0.86 | 1.86 | 0.20 | 0.74 | 0.54 | 1.30 | 0.56 | 4.50 | 0.00 | 0.74 | 0.53 |
| CA042004 | High-affinity copper uptake protein 1 | 0.91 | 0.83 | 5.38 | 0.00 | 1.04 | 0.92 | 2.30 | 0.06 | 1.63 | 0.24 | 0.87 | 0.73 |
| CB510480 | UNKNOWN | 0.96 | 0.91 | 1.91 | 0.11 | 0.57 | 0.14 | 0.91 | 0.79 | 3.70 | 0.00 | 0.73 | 0.39 |
| CA046805 | UNKNOWN | 1.27 | 0.55 | 2.67 | 0.01 | 0.71 | 0.38 | 1.11 | 0.80 | 2.01 | 0.08 | 1.02 | 0.96 |
| CA063936 | UNKNOWN | 1.59 | 0.31 | 1.45 | 0.41 | 0.33 | 0.01 | 0.93 | 0.88 | 1.35 | 0.50 | 0.49 | 0.09 |
| CA037513 | Glutathione peroxidase 2 | 1.56 | 0.31 | 2.83 | 0.03 | 0.36 | 0.03 | 0.99 | 0.98 | 4.09 | 0.00 | 0.66 | 0.37 |
| CA051444 | Vacuolar ATP synthase catalytic subunit A | 1.15 | 0.73 | 2.00 | 0.06 | 0.35 | 0.01 | 0.76 | 0.50 | 1.00 | 1.00 | 0.56 | 0.14 |
| CA055654 | Arachidonate 5-lipoxygenase | 1.54 | 0.45 | 0.16 | 0.00 | 1.03 | 0.96 | 1.14 | 0.82 | 0.49 | 0.24 | 1.73 | 0.36 |
| CA052310 | 5'-3' exoribonuclease 2 | 1.05 | 0.86 | 0.85 | 0.57 | 0.63 | 0.10 | 1.03 | 0.92 | 0.48 | 0.01 | 0.65 | 0.13 |
| CB511091 | U3 small nucleolar ribonucleoprotein protein IMP4 | 1.35 | 0.40 | 2.94 | 0.00 | 1.07 | 0.84 | 1.02 | 0.96 | 2.17 | 0.03 | 1.02 | 0.96 |
| CA050867 | Probable FAD synthetase | 1.46 | 0.41 | 1.70 | 0.34 | 0.28 | 0.01 | 0.77 | 0.56 | 3.48 | 0.03 | 0.44 | 0.07 |
| CB493248 | UNKNOWN | 1.08 | 0.83 | 2.16 | 0.04 | 1.18 | 0.67 | 1.03 | 0.93 | 2.83 | 0.01 | 1.56 | 0.27 |
| CA056057 | UNKNOWN | 2.15 | 0.26 | 0.73 | 0.63 | 0.15 | 0.01 | 1.15 | 0.84 | 1.18 | 0.80 | 0.39 | 0.15 |
| CA052387 | UNKNOWN | 0.72 | 0.72 | 0.31 | 0.21 | 1.73 | 0.55 | 13.96 | 0.01 | 1.25 | 0.81 | 1.15 | 0.88 |
| CA769320 | Fatty acid-binding protein, intestinal | 1.50 | 0.42 | 0.79 | 0.62 | 0.91 | 0.86 | 0.23 | 0.01 | 0.77 | 0.58 | 0.99 | 0.99 |
| CA045033 | Trypsin-1 precursor | 0.93 | 0.87 | 1.90 | 0.16 | 1.56 | 0.33 | 0.80 | 0.65 | 4.20 | 0.00 | 1.39 | 0.47 |
| CA061330 | Ig kappa chain V-IV region JI precursor | 0.98 | 0.92 | 1.23 | 0.38 | 0.59 | 0.01 | 1.43 | 0.15 | 1.42 | 0.14 | 0.87 | 0.44 |
| CB493343 | smart00034, CLECT, C-type lectin (CTL) | 0.87 | 0.72 | 3.09 | 0.00 | 0.93 | 0.83 | 0.47 | 0.08 | 1.88 | 0.08 | 0.81 | 0.55 |
| CB501621 | UNKNOWN | 1.28 | 0.58 | 1.79 | 0.22 | 1.26 | 0.59 | 0.98 | 0.97 | 4.95 | 0.00 | 1.13 | 0.78 |
| CA050997 | 40S ribosomal protein S3a | 0.47 | 0.15 | 0.81 | 0.63 | 0.22 | 0.01 | 0.67 | 0.45 | 1.17 | 0.72 | 0.36 | 0.05 |
| CA056640 | UNKNOWN | 0.40 | 0.01 | 0.52 | 0.06 | 0.95 | 0.88 | 0.51 | 0.06 | 0.73 | 0.35 | 1.71 | 0.15 |
| CB515011 | Galectin-3-binding protein precursor | 1.03 | 0.95 | 4.21 | 0.01 | 0.50 | 0.16 | 1.02 | 0.97 | 2.52 | 0.10 | 0.57 | 0.26 |
| CB516729 | ER lumen protein retaining receptor 2 | 0.90 | 0.84 | 1.07 | 0.88 | 0.15 | 0.01 | 0.81 | 0.68 | 1.24 | 0.61 | 0.12 | 0.01 |
| CA061356 | UNKNOWN | 1.41 | 0.36 | 1.74 | 0.11 | 1.92 | 0.12 | 1.74 | 0.14 | 2.43 | 0.01 | 1.87 | 0.14 |
| CK990923 | Zinc finger protein 330 | 0.49 | 0.03 | 2.09 | 0.03 | 1.66 | 0.13 | 0.87 | 0.66 | 2.75 | 0.00 | 1.39 | 0.31 |
| CK991137 | PREDICTED: similar to Twsg1b protein, partial | 1.70 | 0.22 | 1.06 | 0.88 | 0.81 | 0.59 | 0.85 | 0.70 | 3.21 | 0.00 | 1.05 | 0.90 |
| CB498622 | UNKNOWN | 0.69 | 0.54 | 0.23 | 0.03 | 1.22 | 0.70 | 0.70 | 0.53 | 0.15 | 0.01 | 1.43 | 0.49 |
| CB510736 | DNA-binding protein inhibitor ID-2 | 4.20 | 0.00 | 0.48 | 0.11 | 1.42 | 0.48 | 0.67 | 0.39 | 0.59 | 0.25 | 1.81 | 0.23 |
| CA041690 | Guanine nucleotide-binding protein-like 3-like protein | 0.93 | 0.90 | 0.39 | 0.10 | 1.29 | 0.67 | 0.92 | 0.89 | 0.24 | 0.01 | 1.36 | 0.60 |
| CB509719 | PREDICTED: similar to CC chemokine SCYA103 | 0.65 | 0.10 | 1.18 | 0.50 | 0.79 | 0.39 | 0.46 | 0.00 | 1.53 | 0.09 | 1.15 | 0.60 |
| CA059845 | Triosephosphate isomerase | 0.96 | 0.90 | 0.37 | 0.00 | 0.63 | 0.15 | 0.92 | 0.80 | 0.45 | 0.02 | 0.83 | 0.54 |
| CA054327 | G-protein-signaling modulator 1 | 0.72 | 0.23 | 2.75 | 0.00 | 0.75 | 0.33 | 0.69 | 0.19 | 1.25 | 0.43 | 1.31 | 0.35 |
| CA059521 | UNKNOWN | 1.02 | 0.96 | 0.35 | 0.00 | 1.04 | 0.92 | 0.92 | 0.81 | 0.69 | 0.24 | 1.95 | 0.08 |
| CA043314 | UNKNOWN | 0.40 | 0.01 | 0.49 | 0.02 | 0.68 | 0.19 | 0.45 | 0.02 | 0.63 | 0.11 | 0.97 | 0.90 |
| CA051564 | Transcription factor PU.1 | 0.87 | 0.66 | 2.19 | 0.01 | 0.51 | 0.03 | 0.85 | 0.60 | 1.03 | 0.93 | 0.89 | 0.71 |
| CA062112 | Oncorhynchus mykiss SYPG1 (SYPG1), PHF1 (PHF1) | 0.79 | 0.42 | 0.44 | 0.01 | 0.80 | 0.42 | 1.07 | 0.82 | 0.92 | 0.75 | 0.93 | 0.81 |
| CA064589 | Transposable element Tc1 transposase | 0.47 | 0.00 | 0.54 | 0.01 | 0.90 | 0.57 | 0.80 | 0.31 | 0.93 | 0.75 | 1.00 | 0.99 |
| CB511682 | Nuclear cap-binding protein subunit 1 | 1.41 | 0.34 | 0.39 | 0.01 | 0.65 | 0.24 | 1.38 | 0.37 | 0.73 | 0.37 | 0.98 | 0.96 |
| CA054206 | Serine/threonine-protein phosphatase 2A 65 kDa | 0.95 | 0.88 | 0.40 | 0.01 | 0.84 | 0.61 | 1.22 | 0.62 | 1.07 | 0.84 | 0.78 | 0.46 |
| CA062844 | Septin-5 | 1.19 | 0.66 | 0.37 | 0.01 | 0.92 | 0.86 | 1.06 | 0.89 | 0.75 | 0.43 | 1.25 | 0.60 |
| CA055971 | G2/mitotic-specific cyclin-B1 | 0.77 | 0.30 | 0.99 | 0.98 | 0.46 | 0.01 | 0.76 | 0.29 | 1.38 | 0.19 | 1.05 | 0.86 |
| CB492719 | Voltage-dependent anion-selective channel protein 2 | 0.55 | 0.01 | 0.83 | 0.43 | 0.93 | 0.72 | 0.72 | 0.15 | 1.43 | 0.15 | 1.38 | 0.13 |
| CB492692 | Peroxisomal trans-2-enoyl-CoA reductase | 0.77 | 0.15 | 1.53 | 0.02 | 0.85 | 0.32 | 0.50 | 0.00 | 1.37 | 0.08 | 0.95 | 0.76 |
| CA041001 | UNKNOWN | 0.48 | 0.04 | 0.30 | 0.00 | 0.87 | 0.67 | 0.62 | 0.18 | 0.57 | 0.10 | 1.16 | 0.67 |
| DN048069 | UNKNOWN | 1.33 | 0.45 | 2.76 | 0.01 | 0.48 | 0.05 | 1.12 | 0.76 | 1.52 | 0.25 | 0.46 | 0.04 |
| CA050193 | Granulins precursor | 0.83 | 0.75 | 0.75 | 0.65 | 1.10 | 0.88 | 1.30 | 0.66 | 0.85 | 0.80 | 4.65 | 0.01 |
| CA040196 | Integral membrane protein 2B | 1.58 | 0.02 | 0.88 | 0.48 | 0.78 | 0.23 | 1.32 | 0.12 | 0.61 | 0.01 | 0.66 | 0.05 |
| CA047907 | Heterogeneous nuclear ribonucleoprotein A3 homolog 1 | 3.01 | 0.01 | 0.91 | 0.81 | 1.03 | 0.95 | 1.85 | 0.17 | 1.54 | 0.29 | 1.29 | 0.53 |
| CK991073 | Hemoglobin subunit alpha | 2.73 | 0.01 | 1.56 | 0.21 | 1.69 | 0.11 | 1.08 | 0.84 | 0.89 | 0.73 | 0.84 | 0.59 |
| CB497591 | Protein max | 1.03 | 0.89 | 2.12 | 0.00 | 1.23 | 0.26 | 0.83 | 0.41 | 1.24 | 0.24 | 0.93 | 0.69 |
| CA769463 | EH domain-containing protein 4 | 2.69 | 0.01 | 1.69 | 0.15 | 0.99 | 0.98 | 1.67 | 0.17 | 1.11 | 0.78 | 1.01 | 0.98 |
| CA045890 | UNKNOWN | 2.35 | 0.01 | 1.42 | 0.29 | 1.94 | 0.02 | 1.76 | 0.07 | 1.26 | 0.49 | 1.38 | 0.22 |
| CA039531 | Fibrinogen gamma chain precursor | 1.42 | 0.23 | 2.35 | 0.01 | 1.51 | 0.15 | 1.00 | 0.99 | 1.32 | 0.37 | 1.03 | 0.93 |
| CA054243 | UNKNOWN | 1.30 | 0.58 | 1.07 | 0.87 | 0.23 | 0.00 | 1.38 | 0.50 | 1.26 | 0.59 | 0.82 | 0.66 |
| CK990831 | Leucine-rich repeat flightless-interacting protein 2 | 1.63 | 0.12 | 1.17 | 0.57 | 0.42 | 0.01 | 0.92 | 0.78 | 1.19 | 0.54 | 0.83 | 0.54 |
| CB499653 | Enhancer of mRNA-decapping protein 4 | 1.61 | 0.28 | 1.26 | 0.59 | 0.25 | 0.01 | 1.01 | 0.99 | 1.13 | 0.77 | 0.89 | 0.83 |
| CB512291 | Mitochondrial import inner membrane translocase | 1.61 | 0.12 | 1.97 | 0.01 | 0.87 | 0.59 | 0.78 | 0.40 | 1.68 | 0.06 | 0.95 | 0.86 |
| CB492399 | Glutathione peroxidase 3 precursor | 1.92 | 0.03 | 3.02 | 0.00 | 0.73 | 0.28 | 1.03 | 0.92 | 1.38 | 0.27 | 0.93 | 0.81 |
| CA057512 | E3 ubiquitin-protein ligase MARCH5 | 1.25 | 0.45 | 2.10 | 0.01 | 0.91 | 0.74 | 0.96 | 0.91 | 2.00 | 0.02 | 1.02 | 0.95 |
| CA057098 | Barrier-to-autointegration factor | 1.65 | 0.11 | 0.85 | 0.58 | 0.36 | 0.00 | 1.09 | 0.77 | 0.83 | 0.51 | 0.58 | 0.09 |
| CA061046 | UNKNOWN | 1.78 | 0.08 | 1.56 | 0.20 | 0.37 | 0.01 | 1.31 | 0.41 | 1.14 | 0.70 | 0.86 | 0.66 |
| CB494405 | UNKNOWN | 1.20 | 0.42 | 1.53 | 0.06 | 0.65 | 0.06 | 0.72 | 0.15 | 1.83 | 0.01 | 0.82 | 0.36 |
| CB493478 | Calponin-3 | 1.25 | 0.46 | 2.62 | 0.00 | 0.99 | 0.96 | 0.80 | 0.45 | 2.12 | 0.02 | 0.71 | 0.28 |
| CA053092 | Pre-mRNA-splicing factor 18 | 0.87 | 0.70 | 2.77 | 0.01 | 0.77 | 0.54 | 0.73 | 0.38 | 2.18 | 0.04 | 1.25 | 0.61 |
| CA769275 | Long-chain specific acyl-CoA dehydrogenase, | 1.45 | 0.28 | 2.76 | 0.00 | 1.32 | 0.41 | 0.95 | 0.88 | 1.60 | 0.16 | 1.00 | 1.00 |
| CB510272 | PREDICTED: Danio rerio titin-like (ttnl), mRNA | 1.39 | 0.35 | 1.21 | 0.53 | 0.40 | 0.01 | 0.71 | 0.34 | 1.87 | 0.05 | 0.83 | 0.57 |
| CA043437 | Oncorhynchus keta VT-II gene for vasotocin, complete cds | 2.41 | 0.00 | 1.12 | 0.67 | 0.82 | 0.40 | 0.98 | 0.92 | 1.19 | 0.49 | 0.81 | 0.37 |
| CA060035 | UNKNOWN | 1.71 | 0.27 | 1.87 | 0.19 | 0.19 | 0.00 | 0.96 | 0.93 | 1.98 | 0.16 | 0.57 | 0.23 |
| CB498538 | Sarcoplasmic/endoplasmic reticulum calcium ATPase 1 | 1.08 | 0.81 | 0.89 | 0.70 | 0.67 | 0.18 | 0.85 | 0.60 | 2.46 | 0.01 | 0.86 | 0.60 |
| CK990765 | UNKNOWN | 1.15 | 0.52 | 1.78 | 0.01 | 0.72 | 0.13 | 1.09 | 0.69 | 1.27 | 0.26 | 1.01 | 0.98 |
| CA056419 | UNKNOWN | 1.03 | 0.93 | 0.42 | 0.01 | 0.51 | 0.03 | 0.80 | 0.48 | 0.67 | 0.21 | 0.91 | 0.74 |
| CA058992 | Salmo salar aryl hydrocarbon receptor 2b (AhR2) gene | 1.76 | 0.27 | 1.91 | 0.19 | 0.19 | 0.01 | 1.66 | 0.32 | 2.55 | 0.06 | 0.09 | 0.00 |
| CA769983 | H-2 class II histocompatibility antigen gamma chain | 1.81 | 0.11 | 2.43 | 0.01 | 1.12 | 0.74 | 1.27 | 0.52 | 1.11 | 0.74 | 0.58 | 0.10 |
| CA057469 | Dipeptidyl peptidase 9 | 1.32 | 0.45 | 0.56 | 0.11 | 0.38 | 0.01 | 1.53 | 0.25 | 0.82 | 0.57 | 0.52 | 0.07 |
| CA051510 | UNKNOWN | 1.35 | 0.35 | 2.58 | 0.01 | 0.95 | 0.87 | 1.02 | 0.95 | 2.13 | 0.03 | 0.73 | 0.32 |
| CB509917 | ATP-binding cassette sub-family F member 2 | 1.21 | 0.29 | 1.50 | 0.01 | 0.69 | 0.06 | 1.31 | 0.13 | 1.67 | 0.00 | 0.86 | 0.44 |
| CA041706 | CCAAT/enhancer-binding protein delta | 1.37 | 0.46 | 1.14 | 0.75 | 0.48 | 0.09 | 0.94 | 0.89 | 1.64 | 0.24 | 0.32 | 0.01 |
| CA039055 | Complement factor B precursor | 1.53 | 0.10 | 1.83 | 0.01 | 0.81 | 0.36 | 0.98 | 0.93 | 1.58 | 0.05 | 0.99 | 0.96 |
| CA040413 | Trypsin-3 precursor | 1.30 | 0.39 | 2.91 | 0.00 | 1.15 | 0.63 | 1.18 | 0.59 | 1.55 | 0.11 | 0.75 | 0.33 |
| CB494098 | Mannosyl-oligosaccharide glucosidase | 1.28 | 0.22 | 1.31 | 0.14 | 1.01 | 0.97 | 1.11 | 0.61 | 1.61 | 0.01 | 0.74 | 0.11 |
| CB494043 | Ubiquitin-conjugating enzyme E2 N | 0.81 | 0.63 | 0.96 | 0.92 | 0.35 | 0.01 | 1.01 | 0.98 | 1.56 | 0.27 | 0.59 | 0.19 |
| CA057716 | Splicing factor arginine/serine-rich 11 | 1.28 | 0.18 | 0.98 | 0.92 | 1.23 | 0.26 | 1.04 | 0.84 | 1.64 | 0.01 | 0.95 | 0.77 |
| CA051515 | Cellular retinaldehyde-binding protein | 1.23 | 0.71 | 5.32 | 0.00 | 0.98 | 0.98 | 1.46 | 0.50 | 0.91 | 0.86 | 1.47 | 0.46 |
| CA040505 | Oncorhynchus mykiss VHSV-induced protein-10 mRNA | 3.45 | 0.00 | 2.07 | 0.06 | 1.33 | 0.54 | 1.66 | 0.20 | 1.26 | 0.54 | 1.73 | 0.19 |
| CA062766 | Hemicentin-1 precursor | 1.81 | 0.10 | 0.62 | 0.16 | 0.35 | 0.01 | 0.99 | 0.98 | 1.22 | 0.54 | 0.50 | 0.10 |
| CA039330 | Protein NipSnap2 | 1.52 | 0.19 | 0.94 | 0.85 | 0.40 | 0.00 | 1.74 | 0.11 | 1.00 | 0.99 | 0.54 | 0.04 |
| CA059041 | Dihydropyrimidinase-related protein 3 | 1.25 | 0.45 | 0.79 | 0.40 | 0.61 | 0.06 | 1.37 | 0.25 | 1.03 | 0.93 | 0.46 | 0.01 |
| CA063234 | Cornichon homolog 4 | 2.16 | 0.01 | 1.23 | 0.46 | 0.26 | 0.00 | 1.78 | 0.06 | 1.35 | 0.29 | 0.26 | 0.00 |
| CB517961 | Histone H3.3 | 0.76 | 0.20 | 0.58 | 0.01 | 0.87 | 0.51 | 1.05 | 0.82 | 1.01 | 0.97 | 0.69 | 0.07 |
| CA038694 | UNKNOWN | 1.76 | 0.35 | 2.49 | 0.18 | 0.22 | 0.01 | 1.10 | 0.87 | 1.09 | 0.90 | 0.60 | 0.38 |
| CA054110 | Tether containing UBX domain for GLUT4 | 0.89 | 0.83 | 0.37 | 0.07 | 0.51 | 0.20 | 1.46 | 0.50 | 0.25 | 0.01 | 0.91 | 0.86 |
| CB492003 | Serine/threonine-protein kinase 12 | 1.32 | 0.38 | 2.35 | 0.01 | 0.68 | 0.21 | 1.23 | 0.51 | 1.51 | 0.18 | 0.64 | 0.15 |
| CB503189 | 60S ribosomal protein L12 | 0.77 | 0.71 | 0.79 | 0.69 | 0.12 | 0.01 | 0.97 | 0.97 | 0.92 | 0.88 | 0.11 | 0.01 |
| CA044542 | Hypoxia up-regulated protein 1 precursor | 0.88 | 0.78 | 1.75 | 0.26 | 0.32 | 0.03 | 0.52 | 0.17 | 1.70 | 0.28 | 0.24 | 0.01 |
| CA051939 | UNKNOWN | 0.89 | 0.69 | 2.12 | 0.01 | 0.80 | 0.44 | 0.92 | 0.78 | 1.76 | 0.06 | 0.75 | 0.31 |
| CA039857 | DNA repair endonuclease XPF | 3.08 | 0.01 | 1.26 | 0.59 | 0.82 | 0.61 | 1.42 | 0.39 | 1.05 | 0.90 | 0.81 | 0.58 |
| CA057723 | UNKNOWN | 0.66 | 0.28 | 2.80 | 0.01 | 0.70 | 0.33 | 0.90 | 0.77 | 1.44 | 0.32 | 0.62 | 0.20 |
| CA055827 | UNKNOWN | 1.52 | 0.38 | 3.14 | 0.01 | 1.31 | 0.53 | 1.72 | 0.26 | 1.21 | 0.64 | 0.79 | 0.58 |
| CB486907 | WD repeat protein 61 | 0.76 | 0.38 | 2.50 | 0.00 | 0.72 | 0.28 | 0.68 | 0.23 | 2.19 | 0.01 | 0.92 | 0.79 |
| CA062569 | UNKNOWN | 1.07 | 0.85 | 1.35 | 0.39 | 0.37 | 0.01 | 1.13 | 0.74 | 1.26 | 0.50 | 0.85 | 0.66 |
| CB492169 | 40S ribosomal protein S15 | 1.33 | 0.31 | 0.72 | 0.29 | 0.62 | 0.13 | 1.38 | 0.26 | 0.90 | 0.73 | 0.43 | 0.01 |
| CA052492 | Regulator of G-protein signaling 5 | 2.45 | 0.01 | 0.98 | 0.95 | 0.66 | 0.20 | 1.18 | 0.61 | 0.69 | 0.21 | 0.78 | 0.44 |
| CA045270 | U3 small nucleolar ribonucleoprotein protein IMP4 | 2.24 | 0.00 | 1.27 | 0.25 | 1.03 | 0.87 | 0.84 | 0.39 | 1.43 | 0.09 | 1.07 | 0.72 |
| CB511433 | UNKNOWN | 1.05 | 0.84 | 0.54 | 0.01 | 0.87 | 0.56 | 0.99 | 0.96 | 1.15 | 0.55 | 0.74 | 0.20 |
| CB498219 | 24-dehydrocholesterol reductase precursor | 3.39 | 0.00 | 0.97 | 0.93 | 0.53 | 0.07 | 1.84 | 0.12 | 1.01 | 0.99 | 0.63 | 0.18 |
| CB492261 | S.alpinus DNA for IgM heavy chain constant region | 0.78 | 0.40 | 2.58 | 0.00 | 1.12 | 0.70 | 0.56 | 0.06 | 1.69 | 0.07 | 1.03 | 0.93 |
| CA057532 | UNKNOWN | 0.77 | 0.58 | 3.41 | 0.01 | 0.71 | 0.41 | 1.09 | 0.86 | 1.93 | 0.12 | 1.00 | 0.99 |
| CA037885 | Cytochrome c oxidase polypeptide VIa, mitochondrial | 1.23 | 0.63 | 0.82 | 0.66 | 0.26 | 0.00 | 1.02 | 0.96 | 0.99 | 0.98 | 0.42 | 0.05 |
| CB510287 | Myosin light chain 3, skeletal muscle isoform | 0.90 | 0.82 | 0.43 | 0.06 | 0.74 | 0.54 | 0.29 | 0.01 | 1.87 | 0.16 | 0.85 | 0.73 |
| CB492678 | Profilin-2 | 0.66 | 0.66 | 0.46 | 0.42 | 0.09 | 0.01 | 0.87 | 0.89 | 0.71 | 0.72 | 0.27 | 0.15 |
| CB494071 | 14-3-3-like protein GF14-F | 1.80 | 0.19 | 0.26 | 0.00 | 0.81 | 0.62 | 1.14 | 0.77 | 1.06 | 0.89 | 0.77 | 0.55 |
| CA053153 | Uncharacterized protein C9orf97 homolog | 1.84 | 0.13 | 2.61 | 0.00 | 1.13 | 0.73 | 1.54 | 0.23 | 1.61 | 0.13 | 0.82 | 0.56 |
| CA059752 | Macrophage erythroblast attacher | 0.86 | 0.71 | 0.74 | 0.44 | 0.97 | 0.93 | 1.53 | 0.32 | 0.36 | 0.01 | 0.93 | 0.86 |
| CA054769 | Carbonyl reductase [NADPH] 1 | 1.02 | 0.94 | 0.44 | 0.01 | 0.62 | 0.18 | 0.75 | 0.36 | 1.00 | 1.00 | 0.82 | 0.56 |
| CA063023 | Lamin-B receptor | 1.07 | 0.82 | 2.66 | 0.00 | 0.91 | 0.71 | 1.41 | 0.28 | 1.51 | 0.17 | 0.70 | 0.16 |
| CA057486 | Lathosterol oxidase | 1.39 | 0.37 | 2.38 | 0.01 | 1.16 | 0.67 | 1.00 | 0.99 | 1.19 | 0.59 | 0.61 | 0.18 |
| CA039482 | Endothelial differentiation-related factor 1 homolog | 1.45 | 0.13 | 2.25 | 0.00 | 0.93 | 0.75 | 1.41 | 0.16 | 1.15 | 0.54 | 0.93 | 0.75 |
| CB508092 | UNKNOWN | 0.59 | 0.61 | 0.04 | 0.01 | 0.77 | 0.77 | 3.62 | 0.33 | 0.61 | 0.68 | 0.64 | 0.62 |
| CB517495 | Nuclear pore complex protein Nup88 | 0.55 | 0.43 | 1.32 | 0.69 | 1.36 | 0.65 | 0.10 | 0.01 | 0.89 | 0.89 | 0.96 | 0.96 |
| CA048604 | UNKNOWN | 1.46 | 0.34 | 4.43 | 0.00 | 1.05 | 0.90 | 1.03 | 0.93 | 1.52 | 0.32 | 0.75 | 0.49 |
| CA051536 | Mitochondrial 28S ribosomal protein S29 | 2.01 | 0.00 | 1.40 | 0.08 | 1.73 | 0.01 | 1.44 | 0.06 | 0.87 | 0.47 | 0.86 | 0.42 |
| CA060972 | EGF-like module-containing mucin-like hormone receptor | 1.47 | 0.16 | 0.46 | 0.00 | 0.70 | 0.18 | 1.51 | 0.14 | 0.55 | 0.03 | 1.23 | 0.43 |
| CA041777 | U2 small nuclear ribonucleoprotein B'' | 1.20 | 0.54 | 1.85 | 0.01 | 0.85 | 0.48 | 0.73 | 0.29 | 1.62 | 0.05 | 1.01 | 0.97 |
| CA054490 | Transmembrane protein 85 | 1.33 | 0.16 | 2.11 | 0.00 | 0.98 | 0.94 | 0.97 | 0.89 | 1.27 | 0.22 | 0.93 | 0.77 |
| CA043697 | Ribonuclease UK114 | 1.44 | 0.23 | 2.69 | 0.00 | 1.23 | 0.48 | 1.14 | 0.66 | 1.19 | 0.58 | 0.91 | 0.75 |
| CB497308 | Tubulin alpha chain | 1.20 | 0.45 | 0.53 | 0.00 | 0.67 | 0.07 | 0.85 | 0.49 | 0.72 | 0.10 | 0.48 | 0.00 |
| CB511584 | Oncorhynchus tshawytscha virus-inducible stress protein | 1.38 | 0.32 | 2.62 | 0.00 | 1.30 | 0.38 | 1.00 | 1.00 | 2.02 | 0.02 | 1.14 | 0.66 |
| CA043836 | Phosducin-like protein 3 | 1.14 | 0.49 | 1.55 | 0.01 | 1.11 | 0.58 | 1.26 | 0.22 | 1.54 | 0.02 | 1.02 | 0.91 |
| CA769703 | Ubiquinol-cytochrome-c reductase complex core protein 2 | 1.28 | 0.76 | 1.36 | 0.68 | 0.07 | 0.00 | 1.27 | 0.77 | 1.67 | 0.49 | 0.60 | 0.52 |
| CA043311 | UNKNOWN | 1.33 | 0.42 | 3.26 | 0.00 | 0.67 | 0.23 | 0.97 | 0.94 | 2.65 | 0.01 | 0.62 | 0.15 |
| DR695395 | UNKNOWN | 1.46 | 0.38 | 3.00 | 0.01 | 0.50 | 0.10 | 0.83 | 0.69 | 1.41 | 0.40 | 0.89 | 0.77 |
| CA050636 | Bifunctional methylenetetrahydrofolate | 0.73 | 0.32 | 2.29 | 0.01 | 1.13 | 0.68 | 0.51 | 0.04 | 1.82 | 0.06 | 1.62 | 0.11 |
| CA050751 | Asparaginyl-tRNA synthetase, cytoplasmic | 0.93 | 0.73 | 1.92 | 0.00 | 1.01 | 0.96 | 0.74 | 0.17 | 1.58 | 0.03 | 1.07 | 0.74 |
| CA041728 | UNKNOWN | 0.57 | 0.13 | 0.87 | 0.72 | 1.24 | 0.57 | 0.35 | 0.01 | 1.24 | 0.57 | 0.84 | 0.64 |
| CA059711 | UNKNOWN | 1.38 | 0.28 | 2.39 | 0.00 | 0.70 | 0.22 | 1.77 | 0.06 | 1.40 | 0.25 | 0.80 | 0.44 |
| CA053693 | UNKNOWN | 1.77 | 0.17 | 3.79 | 0.00 | 0.75 | 0.47 | 0.73 | 0.43 | 2.20 | 0.05 | 0.94 | 0.87 |
| CB496736 | Cytochrome c oxidase subunit 5A, mitochondrial | 2.51 | 0.02 | 1.05 | 0.88 | 1.17 | 0.67 | 1.17 | 0.66 | 1.35 | 0.38 | 3.09 | 0.00 |
| CA063839 | Ketohexokinase | 1.04 | 0.90 | 2.94 | 0.00 | 1.22 | 0.55 | 0.57 | 0.11 | 1.64 | 0.14 | 1.03 | 0.93 |
| CA046470 | Oncorhynchus mykiss CD59-like protein (CD59) mRNA, | 1.21 | 0.67 | 4.27 | 0.01 | 0.86 | 0.74 | 0.88 | 0.78 | 2.46 | 0.07 | 0.97 | 0.94 |
| CB516208 | Ubiquitin-like protein 7 | 0.97 | 0.94 | 3.31 | 0.00 | 1.28 | 0.52 | 0.85 | 0.67 | 2.36 | 0.04 | 0.73 | 0.42 |
| CA050956 | UNKNOWN | 0.83 | 0.54 | 2.06 | 0.01 | 1.29 | 0.36 | 0.49 | 0.02 | 1.42 | 0.21 | 2.24 | 0.01 |
| CA050921 | Tubulin epsilon chain | 1.22 | 0.40 | 1.81 | 0.01 | 0.56 | 0.02 | 1.05 | 0.81 | 1.39 | 0.13 | 1.09 | 0.74 |
| CB502598 | Tubulin-specific chaperone A | 0.98 | 0.97 | 1.05 | 0.91 | 1.17 | 0.67 | 1.39 | 0.40 | 3.41 | 0.00 | 0.81 | 0.57 |
| CB499793 | Lipoprotein lipase precursor | 1.09 | 0.76 | 2.12 | 0.01 | 0.89 | 0.69 | 0.69 | 0.19 | 1.45 | 0.17 | 1.15 | 0.63 |
| BU965649 | Glucose-6-phosphate isomerase | 1.22 | 0.59 | 3.44 | 0.00 | 0.61 | 0.12 | 1.45 | 0.31 | 1.55 | 0.17 | 0.98 | 0.94 |
| CB501058 | putative acyl-CoA dehydrogenase | 0.87 | 0.71 | 4.41 | 0.00 | 0.81 | 0.56 | 0.69 | 0.33 | 2.34 | 0.03 | 1.45 | 0.30 |
| CK991256 | Subunit of the THO complex | 1.64 | 0.22 | 2.55 | 0.06 | 0.25 | 0.00 | 0.74 | 0.45 | 0.84 | 0.71 | 0.90 | 0.81 |
| CB509577 | Prolargin precursor | 2.11 | 0.30 | 0.24 | 0.05 | 0.26 | 0.10 | 1.46 | 0.59 | 0.17 | 0.01 | 0.85 | 0.84 |
| CA052201 | Patched domain-containing protein 1 | 1.24 | 0.47 | 1.23 | 0.47 | 0.77 | 0.41 | 1.13 | 0.67 | 2.12 | 0.01 | 0.57 | 0.08 |
| CA050426 | Hemoglobin subunit alpha | 0.85 | 0.55 | 0.94 | 0.78 | 0.91 | 0.74 | 0.95 | 0.84 | 1.48 | 0.09 | 0.44 | 0.01 |
| CB493852 | Proteasome activator complex subunit 1 | 1.92 | 0.01 | 1.79 | 0.03 | 0.81 | 0.35 | 0.74 | 0.21 | 1.62 | 0.06 | 0.72 | 0.16 |
| CB493927 | RNA polymerase II mediator complex subunit 10 | 1.31 | 0.36 | 1.44 | 0.21 | 0.76 | 0.34 | 0.93 | 0.81 | 2.11 | 0.01 | 0.76 | 0.35 |
| CA037505 | UNKNOWN | 1.17 | 0.53 | 1.11 | 0.70 | 1.28 | 0.27 | 0.52 | 0.01 | 1.05 | 0.87 | 1.26 | 0.31 |
| CB496419 | F-actin capping protein subunit alpha-1 | 0.86 | 0.64 | 1.59 | 0.19 | 0.89 | 0.70 | 0.37 | 0.00 | 1.35 | 0.38 | 1.12 | 0.71 |
| CB494589 | Glycogen phosphorylase, muscle form | 1.48 | 0.47 | 1.43 | 0.49 | 0.46 | 0.15 | 1.01 | 0.98 | 3.96 | 0.01 | 0.40 | 0.09 |
| CB498109 | SWI/SNF-related matrix-associated | 0.82 | 0.66 | 0.89 | 0.82 | 0.86 | 0.74 | 0.20 | 0.00 | 1.31 | 0.57 | 0.88 | 0.78 |
| CB515375 | Hemoglobin subunit alpha-4 | 0.82 | 0.53 | 0.77 | 0.33 | 1.30 | 0.55 | 0.93 | 0.81 | 0.97 | 0.91 | 0.26 | 0.00 |
| CB497015 | UNKNOWN | 1.49 | 0.21 | 2.32 | 0.01 | 0.88 | 0.70 | 1.29 | 0.42 | 1.81 | 0.05 | 1.10 | 0.78 |
| CA045932 | Probable NADPH reductase TAH18 | 1.83 | 0.03 | 1.02 | 0.93 | 0.78 | 0.41 | 1.05 | 0.84 | 1.33 | 0.28 | 2.45 | 0.00 |
| CA063158 | Inositol-3-phosphate synthase | 1.67 | 0.16 | 1.12 | 0.76 | 1.29 | 0.46 | 1.62 | 0.18 | 0.37 | 0.01 | 0.88 | 0.72 |
| CB497151 | Bos taurus COL12 mRNA for type XII collagen NC1 | 1.13 | 0.64 | 0.49 | 0.00 | 0.85 | 0.55 | 1.10 | 0.71 | 0.87 | 0.55 | 0.75 | 0.27 |
| CB509890 | Natterin-like protein | 1.19 | 0.67 | 0.33 | 0.01 | 0.91 | 0.81 | 1.52 | 0.32 | 0.62 | 0.20 | 1.01 | 0.98 |
| CB504002 | 40S ribosomal protein S18 | 0.69 | 0.31 | 0.43 | 0.01 | 0.84 | 0.59 | 1.12 | 0.78 | 0.60 | 0.12 | 0.87 | 0.67 |
| CA053670 | Glucosidase 2 subunit beta precursor | 1.26 | 0.43 | 1.04 | 0.87 | 0.98 | 0.94 | 0.80 | 0.44 | 1.95 | 0.01 | 1.01 | 0.97 |
| CB497273 | 40S ribosomal protein S10 | 1.12 | 0.61 | 1.05 | 0.82 | 1.01 | 0.97 | 0.82 | 0.38 | 1.82 | 0.01 | 0.96 | 0.86 |
| CB510170 | Sarcoplasmic/endoplasmic reticulum calcium ATPase 1 | 1.04 | 0.94 | 0.58 | 0.28 | 1.35 | 0.63 | 0.24 | 0.01 | 1.56 | 0.37 | 0.99 | 0.99 |
| CA054098 | Eukaryotic translation initiation factor 2 subunit 2 | 0.80 | 0.55 | 0.38 | 0.01 | 0.85 | 0.66 | 1.18 | 0.66 | 0.74 | 0.42 | 0.93 | 0.84 |
| CA768033 | Coatomer subunit alpha | 1.76 | 0.21 | 0.69 | 0.43 | 3.23 | 0.01 | 1.34 | 0.52 | 0.79 | 0.60 | 1.76 | 0.19 |
| CA053777 | Schistosoma japonicum SJCHGC04882 protein mRNA, | 1.54 | 0.02 | 1.31 | 0.10 | 0.99 | 0.97 | 1.66 | 0.01 | 1.08 | 0.64 | 0.77 | 0.12 |
| CB497529 | Actin, alpha cardiac muscle 1 | 0.84 | 0.50 | 0.50 | 0.01 | 0.95 | 0.85 | 1.08 | 0.77 | 1.55 | 0.08 | 0.90 | 0.68 |
| CB516498 | Plexin-A1 precursor | 3.03 | 0.01 | 0.99 | 0.97 | 0.65 | 0.21 | 2.06 | 0.06 | 1.62 | 0.14 | 1.11 | 0.77 |
| CA037115 | UNKNOWN | 2.57 | 0.01 | 1.85 | 0.07 | 1.49 | 0.23 | 1.18 | 0.63 | 1.21 | 0.59 | 1.14 | 0.70 |
| CA061258 | ATP-dependent DNA helicase 2 subunit 2 | 2.00 | 0.10 | 1.16 | 0.72 | 0.94 | 0.88 | 2.88 | 0.01 | 1.30 | 0.52 | 1.02 | 0.96 |
| CB510009 | two component transcriptional regulator, | 1.53 | 0.28 | 0.85 | 0.66 | 2.52 | 0.01 | 1.48 | 0.31 | 0.80 | 0.55 | 0.98 | 0.95 |
| CB504468 | Elastase-1 | 5.44 | 0.00 | 0.66 | 0.35 | 3.14 | 0.01 | 0.93 | 0.86 | 0.93 | 0.89 | 2.11 | 0.10 |
| CB511768 | Malate synthase | 1.29 | 0.24 | 0.65 | 0.07 | 0.73 | 0.14 | 1.34 | 0.19 | 2.00 | 0.00 | 0.74 | 0.17 |
| CB493401 | Nucleoside diphosphate kinase A | 1.31 | 0.36 | 0.50 | 0.01 | 0.97 | 0.91 | 1.00 | 1.00 | 0.88 | 0.63 | 1.06 | 0.82 |
| CA062184 | CTF18, chromosome transmission fidelity factor 18 | 1.71 | 0.34 | 0.27 | 0.01 | 0.58 | 0.25 | 0.81 | 0.71 | 0.66 | 0.37 | 0.77 | 0.58 |
| CA061152 | UNKNOWN | 1.76 | 0.09 | 1.35 | 0.34 | 0.86 | 0.64 | 0.36 | 0.00 | 1.48 | 0.22 | 0.87 | 0.66 |
| CB510533 | Salmo salar RAF1a gene for serine/threonine protein | 1.42 | 0.26 | 0.51 | 0.03 | 1.13 | 0.69 | 0.35 | 0.00 | 0.70 | 0.23 | 1.10 | 0.74 |
| CA058816 | UNKNOWN | 2.40 | 0.00 | 1.06 | 0.82 | 1.42 | 0.21 | 1.20 | 0.54 | 0.78 | 0.37 | 1.19 | 0.54 |
| CA037968 | UNKNOWN | 5.30 | 0.00 | 0.87 | 0.76 | 2.01 | 0.16 | 2.07 | 0.14 | 1.22 | 0.73 | 1.21 | 0.69 |
| CA042507 | Actin, cytoplasmic 1 | 0.85 | 0.55 | 0.46 | 0.01 | 1.98 | 0.11 | 0.86 | 0.59 | 0.57 | 0.04 | 1.85 | 0.15 |
| CA039186 | UNKNOWN | 1.97 | 0.00 | 1.80 | 0.01 | 1.15 | 0.53 | 1.06 | 0.78 | 0.93 | 0.73 | 1.27 | 0.28 |
| CA060730 | UNKNOWN | 0.73 | 0.35 | 0.47 | 0.01 | 1.12 | 0.73 | 1.01 | 0.98 | 1.00 | 1.00 | 1.25 | 0.48 |
| CB492136 | Cystatin precursor | 2.05 | 0.01 | 1.52 | 0.19 | 0.99 | 0.98 | 0.82 | 0.47 | 1.11 | 0.74 | 1.00 | 0.99 |
| CK991090 | Glycerol-3-phosphate dehydrogenase 1-like protein | 4.02 | 0.01 | 1.12 | 0.81 | 1.00 | 1.00 | 1.13 | 0.80 | 1.64 | 0.30 | 1.12 | 0.83 |
| CB507384 | Fatty acid-binding protein, adipocyte | 2.96 | 0.01 | 0.81 | 0.56 | 1.40 | 0.35 | 0.78 | 0.50 | 0.55 | 0.11 | 0.67 | 0.27 |
| CB494424 | Serine/threonine-protein phosphatase 2A catalytic | 2.22 | 0.01 | 1.20 | 0.54 | 0.93 | 0.84 | 1.15 | 0.65 | 0.91 | 0.75 | 0.94 | 0.86 |
| CA051388 | Olfactomedin precursor | 0.85 | 0.62 | 2.53 | 0.01 | 1.31 | 0.43 | 0.99 | 0.98 | 2.21 | 0.03 | 1.68 | 0.17 |
| CB505490 | Zinc finger protein 207 | 0.71 | 0.08 | 0.61 | 0.01 | 0.97 | 0.91 | 1.03 | 0.89 | 1.02 | 0.91 | 1.20 | 0.43 |
| CA044386 | UNKNOWN | 1.31 | 0.25 | 0.74 | 0.20 | 1.24 | 0.38 | 1.56 | 0.07 | 0.87 | 0.53 | 2.06 | 0.01 |
| CB499139 | Nucleoplasmin-like protein NO29 | 0.89 | 0.63 | 2.44 | 0.00 | 0.80 | 0.33 | 0.89 | 0.63 | 2.06 | 0.01 | 0.76 | 0.25 |
| CB493138 | Cytochrome c oxidase copper chaperone | 0.63 | 0.09 | 0.55 | 0.03 | 1.57 | 0.09 | 0.81 | 0.42 | 0.50 | 0.01 | 1.41 | 0.19 |
| CB499791 | Far upstream element-binding protein 2 | 0.74 | 0.10 | 1.06 | 0.74 | 0.83 | 0.28 | 0.61 | 0.01 | 1.03 | 0.88 | 1.12 | 0.52 |
| CB492725 | Beta-enolase | 0.61 | 0.30 | 0.47 | 0.11 | 0.77 | 0.57 | 0.24 | 0.00 | 0.88 | 0.79 | 1.16 | 0.75 |
| CB492879 | Betaine--homocysteine S-methyltransferase 1 | 0.73 | 0.09 | 1.00 | 0.99 | 0.77 | 0.18 | 1.09 | 0.64 | 1.61 | 0.01 | 0.93 | 0.70 |
| CA041794 | UNKNOWN | 4.21 | 0.04 | 1.26 | 0.70 | 2.19 | 0.21 | 1.38 | 0.62 | 0.94 | 0.92 | 4.77 | 0.01 |
| CA036551 | Nattectin precursor | 0.74 | 0.17 | 1.16 | 0.46 | 2.03 | 0.00 | 1.07 | 0.74 | 1.26 | 0.27 | 2.51 | 0.00 |
| CB510006 | UNKNOWN | 1.27 | 0.40 | 0.94 | 0.83 | 1.61 | 0.09 | 1.00 | 0.99 | 0.80 | 0.42 | 2.17 | 0.01 |
| CK990788 | Retinal rod rhodopsin-sensitive cGMP 3',5'-cyclic | 1.20 | 0.61 | 0.83 | 0.58 | 0.90 | 0.75 | 2.55 | 0.01 | 0.61 | 0.17 | 2.00 | 0.05 |
| CA041517 | Mitochondrial Rho GTPase 2 | 1.09 | 0.76 | 0.64 | 0.14 | 2.14 | 0.01 | 0.91 | 0.74 | 1.35 | 0.31 | 1.48 | 0.16 |
| CK990543 | leukocyte elastase inhibitor [Oncorhynchus mykiss] | 1.25 | 0.61 | 0.71 | 0.42 | 1.74 | 0.23 | 3.67 | 0.01 | 0.67 | 0.35 | 1.40 | 0.47 |
| CA041843 | UNKNOWN | 1.54 | 0.37 | 0.25 | 0.00 | 0.90 | 0.83 | 1.96 | 0.17 | 0.41 | 0.06 | 2.10 | 0.12 |
| CB504376 | UNKNOWN | 1.18 | 0.57 | 1.18 | 0.54 | 1.48 | 0.19 | 1.03 | 0.92 | 1.30 | 0.34 | 2.19 | 0.01 |
| CA770531 | UNKNOWN | 1.29 | 0.54 | 1.39 | 0.42 | 1.50 | 0.32 | 3.26 | 0.01 | 1.32 | 0.49 | 1.40 | 0.41 |
| CB498547 | Nuclear protein Hcc-1 | 0.54 | 0.01 | 0.92 | 0.70 | 1.71 | 0.01 | 0.76 | 0.24 | 1.90 | 0.00 | 1.53 | 0.05 |
| CA051052 | UNKNOWN | 0.82 | 0.25 | 0.67 | 0.01 | 0.88 | 0.47 | 0.98 | 0.91 | 0.91 | 0.53 | 1.00 | 0.99 |
| CB510848 | Histone H1.0 | 0.55 | 0.01 | 0.72 | 0.07 | 0.93 | 0.68 | 0.95 | 0.82 | 0.85 | 0.37 | 1.09 | 0.60 |
| CB510423 | UNKNOWN | 0.90 | 0.73 | 0.44 | 0.01 | 1.03 | 0.92 | 1.28 | 0.45 | 0.86 | 0.62 | 1.76 | 0.07 |
| CB493925 | Ictacalcin | 0.72 | 0.14 | 0.59 | 0.01 | 0.92 | 0.69 | 1.07 | 0.77 | 1.02 | 0.93 | 1.15 | 0.52 |
| CB509632 | UNKNOWN | 1.14 | 0.63 | 0.43 | 0.01 | 0.83 | 0.42 | 0.88 | 0.64 | 0.50 | 0.03 | 1.48 | 0.10 |
| CB496864 | Eukaryotic translation initiation factor 3 subunit 5 | 1.54 | 0.18 | 0.43 | 0.01 | 0.75 | 0.38 | 1.54 | 0.20 | 0.69 | 0.20 | 1.59 | 0.18 |
| CA050155 | Coronin-1A | 1.91 | 0.01 | 0.99 | 0.98 | 0.89 | 0.63 | 0.81 | 0.39 | 0.80 | 0.35 | 1.06 | 0.80 |
| CB512930 | Protein transport protein Sec61 subunit beta | 1.18 | 0.27 | 0.78 | 0.18 | 0.70 | 0.00 | 1.33 | 0.06 | 1.11 | 0.59 | 0.86 | 0.21 |
| CB508023 | UNKNOWN | 1.95 | 0.16 | 0.49 | 0.09 | 0.99 | 0.98 | 3.42 | 0.01 | 0.59 | 0.21 | 1.42 | 0.43 |
| CB510981 | Pepsin A precursor | 1.04 | 0.89 | 0.60 | 0.06 | 0.99 | 0.97 | 1.33 | 0.32 | 0.43 | 0.00 | 1.50 | 0.14 |
| CB507647 | Protein SET | 0.91 | 0.64 | 0.51 | 0.00 | 0.88 | 0.56 | 1.42 | 0.10 | 0.74 | 0.17 | 0.81 | 0.32 |
| CA050425 | Mesoderm development candidate 1 | 1.62 | 0.19 | 0.86 | 0.64 | 0.78 | 0.46 | 0.86 | 0.65 | 0.97 | 0.92 | 2.74 | 0.01 |
| CA037557 | 125 kDa kinesin-related protein | 0.54 | 0.01 | 1.01 | 0.97 | 1.19 | 0.41 | 0.88 | 0.57 | 1.04 | 0.85 | 1.26 | 0.28 |
| CA051739 | UNKNOWN | 0.95 | 0.79 | 0.97 | 0.86 | 0.64 | 0.01 | 0.82 | 0.29 | 1.36 | 0.05 | 0.89 | 0.45 |
| CB497463 | UNKNOWN | 1.04 | 0.83 | 0.84 | 0.36 | 1.78 | 0.01 | 0.89 | 0.55 | 0.69 | 0.06 | 1.92 | 0.00 |
| CA063919 | UNKNOWN | 1.02 | 0.93 | 0.75 | 0.17 | 1.11 | 0.63 | 0.51 | 0.00 | 1.20 | 0.39 | 0.96 | 0.84 |
| CA050497 | Uncharacterized protein C11orf2 | 0.95 | 0.88 | 2.31 | 0.01 | 1.32 | 0.32 | 1.20 | 0.57 | 1.31 | 0.38 | 0.69 | 0.19 |
| CK990286 | Ubiquitin | 0.52 | 0.01 | 0.56 | 0.02 | 1.30 | 0.32 | 1.11 | 0.68 | 0.91 | 0.68 | 0.91 | 0.71 |
| CA037501 | UNKNOWN | 0.44 | 0.00 | 0.88 | 0.59 | 0.98 | 0.94 | 0.75 | 0.30 | 1.03 | 0.92 | 1.17 | 0.51 |
| CA052515 | 60S ribosomal protein L11 | 0.50 | 0.01 | 0.63 | 0.04 | 0.82 | 0.40 | 0.87 | 0.56 | 0.87 | 0.51 | 1.05 | 0.82 |
| CB507685 | Mitofusin-1 | 1.31 | 0.36 | 0.61 | 0.08 | 2.39 | 0.00 | 1.30 | 0.38 | 0.61 | 0.09 | 1.76 | 0.05 |
| CN442545 | Cytochrome c oxidase subunit 3 | 0.47 | 0.00 | 0.64 | 0.05 | 0.88 | 0.57 | 0.79 | 0.35 | 1.07 | 0.75 | 1.05 | 0.81 |
| CA044290 | PREDICTED: similar to Y54G11A.7 isoform 1 [Canis | 0.76 | 0.60 | 0.49 | 0.17 | 1.54 | 0.40 | 2.52 | 0.08 | 0.25 | 0.01 | 4.88 | 0.00 |
| CA063145 | Zinc finger protein ZFMSA12A | 1.72 | 0.11 | 0.66 | 0.19 | 1.53 | 0.19 | 1.66 | 0.16 | 0.31 | 0.00 | 2.70 | 0.00 |
| CA062957 | Hematological and neurological expressed 1 protein | 0.70 | 0.24 | 0.26 | 0.00 | 1.34 | 0.36 | 1.01 | 0.97 | 0.34 | 0.00 | 1.47 | 0.23 |
| CA044543 | Apolipoprotein D precursor | 1.24 | 0.51 | 0.76 | 0.36 | 1.71 | 0.09 | 0.71 | 0.29 | 0.92 | 0.80 | 3.30 | 0.00 |
| CB493629 | S-methyl-5-thioadenosine phosphorylase | 1.76 | 0.10 | 0.33 | 0.00 | 1.08 | 0.85 | 1.14 | 0.71 | 0.55 | 0.08 | 1.78 | 0.14 |
| CB501332 | GDP-L-fucose synthetase | 1.25 | 0.46 | 0.41 | 0.00 | 2.11 | 0.02 | 1.25 | 0.46 | 0.57 | 0.07 | 2.64 | 0.00 |
| CB511983 | PREDICTED: similar to LOC152217 protein [Danio rerio] | 3.49 | 0.01 | 0.56 | 0.22 | 0.85 | 0.73 | 2.54 | 0.06 | 0.60 | 0.28 | 0.74 | 0.52 |
| CA043370 | Eukaryotic translation initiation factor 3 subunit 8 | 1.12 | 0.72 | 0.47 | 0.01 | 0.88 | 0.67 | 1.40 | 0.28 | 0.61 | 0.10 | 0.85 | 0.59 |
| CB497295 | NADH dehydrogenase [ubiquinone] 1 beta subcomplex | 0.92 | 0.89 | 0.19 | 0.01 | 1.04 | 0.95 | 0.64 | 0.48 | 0.66 | 0.50 | 1.03 | 0.96 |
| CA038193 | Fatty acid-binding protein, liver | 0.34 | 0.03 | 0.49 | 0.14 | 1.42 | 0.43 | 0.16 | 0.00 | 0.45 | 0.10 | 0.78 | 0.58 |
| CB511315 | Cytochrome c oxidase subunit 1 | 0.91 | 0.84 | 0.47 | 0.08 | 0.81 | 0.65 | 3.31 | 0.01 | 0.65 | 0.31 | 0.83 | 0.68 |
| CB498502 | Homo sapiens fibronectin type III domain containing 3B | 1.05 | 0.84 | 0.38 | 0.00 | 0.76 | 0.31 | 0.81 | 0.40 | 0.69 | 0.21 | 0.92 | 0.75 |
| CA047623 | UNKNOWN | 1.07 | 0.79 | 0.54 | 0.01 | 0.79 | 0.34 | 1.29 | 0.31 | 0.68 | 0.12 | 0.87 | 0.58 |
| CA042054 | C-factor | 2.27 | 0.20 | 0.58 | 0.33 | 1.94 | 0.23 | 0.08 | 0.00 | 0.78 | 0.64 | 1.56 | 0.42 |
| CB498472 | Selenide, water dikinase 2 | 1.01 | 0.98 | 0.40 | 0.01 | 1.46 | 0.30 | 1.22 | 0.55 | 0.43 | 0.01 | 0.96 | 0.91 |
| CB516736 | UNKNOWN | 0.90 | 0.77 | 0.39 | 0.01 | 0.66 | 0.24 | 0.97 | 0.93 | 0.61 | 0.17 | 0.90 | 0.77 |
| CK991312 | Myosin heavy chain, fast skeletal muscle | 0.89 | 0.69 | 0.55 | 0.01 | 0.97 | 0.92 | 1.06 | 0.85 | 1.15 | 0.54 | 0.89 | 0.70 |
| CA038167 | UNKNOWN | 0.94 | 0.81 | 0.83 | 0.47 | 0.96 | 0.86 | 0.40 | 0.00 | 0.82 | 0.46 | 0.88 | 0.62 |
| CA044190 | Oncorhynchus mykiss SYPG1 (SYPG1), PHF1 (PHF1) | 1.06 | 0.82 | 0.51 | 0.01 | 1.04 | 0.90 | 1.20 | 0.49 | 0.69 | 0.15 | 1.25 | 0.42 |
| CB517017 | Nuclear transport factor 2 | 1.19 | 0.52 | 0.46 | 0.00 | 0.95 | 0.85 | 0.78 | 0.36 | 0.52 | 0.01 | 1.09 | 0.74 |
| CA058741 | UNKNOWN | 0.50 | 0.01 | 0.44 | 0.00 | 0.80 | 0.38 | 0.55 | 0.03 | 0.88 | 0.62 | 1.08 | 0.77 |
| CA041903 | NADH dehydrogenase [ubiquinone] 1 beta subcomplex | 1.39 | 0.43 | 0.36 | 0.01 | 2.07 | 0.06 | 1.40 | 0.43 | 0.72 | 0.38 | 1.46 | 0.32 |
| CA055350 | Putative GTP-binding protein 9 | 1.74 | 0.24 | 0.44 | 0.09 | 0.90 | 0.82 | 1.99 | 0.14 | 0.26 | 0.01 | 1.66 | 0.29 |
| CB497013 | Myosin heavy chain, fast skeletal muscle | 0.31 | 0.00 | 0.47 | 0.05 | 0.77 | 0.51 | 0.31 | 0.00 | 1.62 | 0.20 | 1.06 | 0.88 |
| CB498718 | UNKNOWN | 0.83 | 0.47 | 0.52 | 0.01 | 0.87 | 0.54 | 1.03 | 0.89 | 0.83 | 0.45 | 0.96 | 0.87 |
| CN442535 | NADH-ubiquinone oxidoreductase chain 3 | 1.00 | 0.99 | 0.35 | 0.00 | 0.80 | 0.42 | 0.95 | 0.87 | 0.39 | 0.00 | 1.10 | 0.73 |
| CB486790 | Nuclear receptor coactivator 5 | 1.01 | 0.99 | 1.09 | 0.75 | 0.49 | 0.01 | 0.76 | 0.37 | 1.03 | 0.90 | 1.25 | 0.40 |
| CA768839 | UNKNOWN | 0.47 | 0.01 | 0.72 | 0.18 | 1.17 | 0.51 | 0.52 | 0.03 | 0.91 | 0.71 | 1.36 | 0.22 |
| CA062018 | UNKNOWN | 0.92 | 0.84 | 0.35 | 0.01 | 0.60 | 0.31 | 0.81 | 0.61 | 0.51 | 0.10 | 1.24 | 0.66 |
| CA050852 | Ig kappa chain V-IV region JI precursor | 1.08 | 0.81 | 2.29 | 0.01 | 0.91 | 0.75 | 0.90 | 0.73 | 1.23 | 0.50 | 1.10 | 0.75 |
| CA061566 | Salmo salar zonadhesin-like gene, complete cds and 3' | 0.87 | 0.72 | 0.41 | 0.03 | 0.63 | 0.26 | 0.87 | 0.73 | 0.24 | 0.00 | 0.76 | 0.50 |
| CB503505 | Oncorhynchus mykiss nonclassical MHC class I antigen | 0.96 | 0.91 | 0.41 | 0.01 | 1.18 | 0.64 | 1.45 | 0.25 | 0.63 | 0.16 | 1.18 | 0.65 |
| CB510886 | Creatine kinase B-type | 0.80 | 0.40 | 0.47 | 0.01 | 0.96 | 0.88 | 0.85 | 0.51 | 1.26 | 0.38 | 0.94 | 0.82 |
| CA061758 | Tubulin-specific chaperone D | 0.60 | 0.34 | 0.45 | 0.10 | 1.14 | 0.77 | 1.40 | 0.58 | 0.25 | 0.01 | 1.17 | 0.72 |
| CB510778 | Translation initiation factor eIF-2B subunit alpha | 0.85 | 0.60 | 0.44 | 0.01 | 0.76 | 0.39 | 0.88 | 0.69 | 0.73 | 0.28 | 1.07 | 0.82 |
| CA050527 | Eukaryotic translation initiation factor 4B | 0.40 | 0.00 | 1.65 | 0.05 | 0.83 | 0.46 | 0.56 | 0.02 | 1.41 | 0.17 | 0.71 | 0.19 |
| CB494039 | 14-3-3 protein beta/alpha-1 | 0.65 | 0.19 | 0.41 | 0.01 | 1.02 | 0.96 | 1.04 | 0.91 | 0.58 | 0.09 | 1.04 | 0.90 |
| CB517743 | Salmo salar aryl hydrocarbon receptor 2 gamma (AhR2g) | 0.53 | 0.01 | 0.87 | 0.56 | 0.75 | 0.21 | 0.51 | 0.01 | 0.77 | 0.25 | 0.94 | 0.78 |
| CA037966 | Cyclin-Y | 0.53 | 0.07 | 0.47 | 0.01 | 1.22 | 0.52 | 0.54 | 0.08 | 0.75 | 0.31 | 1.29 | 0.40 |
| CA060569 | UNKNOWN | 0.69 | 0.21 | 0.49 | 0.01 | 0.76 | 0.31 | 0.77 | 0.37 | 0.85 | 0.55 | 1.02 | 0.94 |
| CB514125 | Neurofilament light polypeptide | 1.58 | 0.04 | 0.50 | 0.00 | 0.71 | 0.10 | 0.85 | 0.45 | 0.78 | 0.27 | 0.98 | 0.91 |
| CB507419 | UNKNOWN | 1.68 | 0.43 | 0.16 | 0.01 | 1.18 | 0.80 | 0.54 | 0.36 | 1.34 | 0.64 | 1.55 | 0.49 |
| CA057252 | VIP36-like protein precursor | 0.86 | 0.45 | 1.78 | 0.01 | 0.92 | 0.67 | 0.79 | 0.22 | 1.26 | 0.26 | 1.05 | 0.78 |
| CA044909 | Cytosolic sulfotransferase 2 | 2.46 | 0.01 | 0.95 | 0.88 | 1.06 | 0.86 | 1.30 | 0.45 | 1.01 | 0.97 | 1.38 | 0.31 |
| CB493659 | Type 2A phosphatase activator TIP41 | 0.98 | 0.94 | 2.63 | 0.01 | 1.40 | 0.36 | 1.18 | 0.64 | 1.05 | 0.89 | 0.98 | 0.96 |
| CB499771 | differentially expressed in FDCP 6 homolog (mouse), | 0.84 | 0.60 | 2.42 | 0.01 | 0.90 | 0.73 | 1.37 | 0.34 | 1.24 | 0.54 | 1.10 | 0.77 |
| CB494170 | Homo sapiens cDNA FLJ43303 fis, clone NOVAR2000136, | 0.84 | 0.54 | 0.80 | 0.45 | 0.87 | 0.61 | 0.86 | 0.60 | 2.23 | 0.01 | 1.01 | 0.96 |
| CB508780 | UNKNOWN | 1.06 | 0.91 | 2.02 | 0.10 | 2.54 | 0.05 | 0.62 | 0.28 | 1.20 | 0.66 | 3.42 | 0.01 |
| CA058544 | Vacuolar ATP synthase subunit H | 0.48 | 0.00 | 0.48 | 0.01 | 1.03 | 0.89 | 0.83 | 0.44 | 1.07 | 0.78 | 1.50 | 0.09 |
| CB515837 | Programmed cell death protein 5 | 0.73 | 0.14 | 0.85 | 0.41 | 1.04 | 0.82 | 0.56 | 0.01 | 0.97 | 0.89 | 1.25 | 0.25 |
| CB494458 | Parvalbumin beta 1 | 3.39 | 0.00 | 0.74 | 0.39 | 0.72 | 0.41 | 1.78 | 0.10 | 1.42 | 0.33 | 0.95 | 0.90 |
| CA053062 | 5-aminolevulinate synthase, nonspecific, mitochondrial | 0.91 | 0.72 | 1.57 | 0.18 | 0.78 | 0.34 | 0.76 | 0.30 | 2.48 | 0.01 | 0.79 | 0.34 |
| CB512516 | BTB/POZ domain-containing protein 10 | 0.89 | 0.54 | 1.56 | 0.04 | 1.11 | 0.61 | 1.00 | 0.99 | 1.82 | 0.01 | 0.88 | 0.53 |
| CB498843 | Proactivator polypeptide precursor | 1.11 | 0.60 | 2.64 | 0.00 | 0.72 | 0.05 | 1.07 | 0.74 | 1.47 | 0.04 | 0.96 | 0.79 |
| CB510281 | Parvalbumin beta 1 | 0.67 | 0.36 | 0.51 | 0.10 | 0.98 | 0.96 | 0.14 | 0.00 | 1.48 | 0.32 | 1.02 | 0.97 |
| CB492660 | Biotinidase precursor | 1.03 | 0.94 | 1.61 | 0.18 | 1.36 | 0.46 | 0.96 | 0.92 | 2.97 | 0.00 | 1.41 | 0.40 |
| CA040710 | Glypican-4 precursor | 2.12 | 0.01 | 0.85 | 0.51 | 1.55 | 0.08 | 0.52 | 0.01 | 1.05 | 0.85 | 1.18 | 0.51 |
| CB515569 | damage specific DNA binding protein 1 | 1.13 | 0.61 | 0.54 | 0.01 | 0.99 | 0.96 | 1.46 | 0.13 | 0.62 | 0.04 | 1.16 | 0.51 |
| CB496548 | Inorganic pyrophosphatase | 1.31 | 0.23 | 0.95 | 0.83 | 1.93 | 0.00 | 0.71 | 0.13 | 1.53 | 0.08 | 2.10 | 0.00 |
| CA037913 | UNKNOWN | 1.00 | 1.00 | 1.02 | 0.96 | 1.65 | 0.08 | 1.09 | 0.76 | 1.12 | 0.69 | 2.17 | 0.01 |
| CA057365 | LAG1 longevity assurance homolog 2 | 1.87 | 0.01 | 0.92 | 0.70 | 0.84 | 0.37 | 1.65 | 0.04 | 1.03 | 0.89 | 0.89 | 0.55 |
| CB503044 | Intermediate filament protein ON3 | 0.35 | 0.00 | 0.70 | 0.22 | 1.14 | 0.68 | 0.66 | 0.18 | 1.10 | 0.73 | 1.33 | 0.36 |
| CB517852 | Methyltransferase-like protein 2 | 0.98 | 0.91 | 0.93 | 0.65 | 1.17 | 0.31 | 0.66 | 0.01 | 1.16 | 0.38 | 1.01 | 0.95 |
| CB512647 | UNKNOWN | 1.73 | 0.03 | 0.79 | 0.35 | 0.73 | 0.21 | 1.91 | 0.01 | 0.86 | 0.54 | 1.36 | 0.22 |
| CA058569 | UNKNOWN | 1.13 | 0.68 | 0.35 | 0.00 | 0.55 | 0.05 | 1.38 | 0.26 | 0.61 | 0.14 | 1.13 | 0.68 |
| CA769480 | Endothelial differentiation-related factor 1 homolog | 1.01 | 0.94 | 1.23 | 0.17 | 0.78 | 0.10 | 0.83 | 0.27 | 1.75 | 0.00 | 1.00 | 0.98 |
| CB493673 | UNKNOWN | 0.48 | 0.00 | 0.70 | 0.10 | 1.01 | 0.95 | 0.67 | 0.11 | 1.05 | 0.82 | 1.20 | 0.36 |
| CB511393 | UNKNOWN | 1.80 | 0.10 | 0.83 | 0.59 | 0.91 | 0.78 | 2.60 | 0.01 | 1.09 | 0.80 | 1.65 | 0.14 |
