# Supplementary material for: Differential gene expression in male and female rainbow trout embryos prior to the onset of gross morphological differentiation of the gonads
Source: BMC Genomics. 2011 Aug 8;12:404. doi: 10.1186/1471-2164-12-404 (PMC3166948; doi:10.1186/1471-2164-12-404)
Supplement: Additional file 2 — Table showing results from GLM analysis. Results from GLM analysis, components that are statistically significant are shown in bold type. Genes identified from the microarray are above the line. [file 1471-2164-12-404-S2.DOCX]

|  | Model |  | dpf |  | Sex |  | QTL |  | dpf*Sex |  | dpf*QTL |  | Sex*QTL |  |
| --- | --- | --- | --- | --- | --- | --- | --- | --- | --- | --- | --- | --- | --- | --- |
| Gene | F-value | P-value | F-value | p-value | F-value | P-value | F-value | P-value | F-value | p-value | F-value | p-value | F-value | p-value |
| *Zonadhesin* | 2.45 | **0.01** | 2.27 | 0.07 | 0.45 | 0.507 | 4.23 | **0.04** | 2.5 | **0.05** | 2.21 | 0.126 | 3.85 | **0.05** |
| *Aromatase* | 11.55 | **<0.001** | 24.06 | **<0.001** | 4.75 | **0.03** | 1.49 | 0.23 | 4.19 | **0.005** | 0.14 | 0.87 | 0.47 | 0.5 |
| *Prostaglandin* | 3.89 | **0.01** | 14.2 | **<0.001** | 1.25 | 0.28 | 0.18 | 0.68 | 1.29 | 0.31 | 0.86 | 0.45 | 0.87 | 0.37 |
| *Coatamer subunit* | 1.18 | 0.38 | 0.94 | 0.41 | 0.73 | 0.41 | 1.34 | 0.27 | 1.15 | 0.34 | 1.31 | 0.3 | 1.77 | 0.2 |
| *Vasa* | 0.4 | 0.91 | 0.4 | 0.68 | 0.6 | 0.45 | 0.29 | 0.6 | 0.31 | 0.74 | 0.46 | 0.64 | 0.4 | 0.54 |
| *wt1* | 7.44 | **<0.001** | 15.58 | **<0.001** | 1.44 | 0.239 | 2.73 | 0.109 | 2.58 | **0.04** | 7.75 | **0.001** | 1.09 | 0.304 |
| *foxl2a* | 2.25 | **0.02** | 4.14 | **0.005** | 0.09 | 0.772 | 0.57 | 0.455 | 2.01 | 0.116 | 1.33 | 0.282 | 0.4 | 0.539 |
| *sox9b1* | 3.61 | **0.001** | 6.73 | **<0.001** | 1.63 | 0.211 | 2.39 | 0.131 | 1.94 | 0.115 | 3.39 | **0.04** | 0.01 | 0.925 |
| *Ovol1* | 14.22 | **<0.001** | 35.63 | **<0.001** | 4.08 | **0.05** | 4.71 | **0.03** | 2.34 | 0.06 | 2.2 | 0.129 | 10.24 | **0.003** |
| *foxl2b* | 3.11 | **0.003** | 6.19 | **0.004** | 0.76 | 0.391 | 0.02 | 0.802 | 1.19 | 0.12 | 0.38 | 0.688 | 4.59 | **0.03** |
| *DMRT1* | 1.95 | **0.05** | 4.02 | **0.006** | 0.22 | 0.642 | 1.08 | 0.306 | 0.95 | 0.445 | 0.86 | 0.473 | 1.42 | 0.242 |
| *Fst* | 2.1 | 0.1 | 1.92 | 0.18 | 1 | 0.34 | 4.98 | **0.04** | 0.41 | 0.67 | 3.37 | 0.06 | 1.5 | 0.24 |
| *GC1* | 0.59 | 0.71 | 1.81 | 0.2 | 0 | 0.95 | 0.55 | 0.47 | 0.15 | 0.86 | 0.21 | 0.82 | 0.46 | 0.77 |
| *Solt1* | 6.62 | **<0.001** | 17.14 | **<0.001** | 3.22 | 0.09 | 6.05 | **0.02** | 2.67 | 0.1 | 5.28 | **0.02** | 0.11 | 0.75 |
| *sox9a1* | 2.67 | 0.3 | 5.04 | **0.02** | 0.36 | 0.56 | 0.06 | 0.81 | 0.17 | 0.85 | 0.6 | 0.56 | 0.02 | 0.88 |
| *IFR* | 1.14 | 0.4 | 2.37 | 0.13 | 1.25 | 0.28 | 0.1 | 0.76 | 0.15 | 0.86 | 0.34 | 0.71 | 3.16 | 0.09 |
| *IHR* | 2.87 | **0.04** | 9.8 | **0.002** | 0.08 | 0.79 | 0.32 | 0.58 | 0.61 | 0.56 | 1.17 | 0.34 | 2.14 | 0.17 |
| *Nr5a1* | 1.38 | 0.29 | 2.95 | 0.08 | 1.57 | 0.23 | 0.46 | 0.5 | 1.42 | 0.28 | 0.47 | 0.63 | 0.68 | 0.42 |
| *Nr0b1* | 2.23 | 0.09 | 3.87 | **0.04** | 3.05 | 0.1 | 2.13 | 0.17 | 1.61 | 0.24 | 1.49 | 0.26 | 0.98 | 0.34 |
| *AMH* | 0.91 | 0.54 | 0.75 | 0.49 | 0.36 | 0.56 | 1.84 | 0.2 | 0.42 | 0.66 | 1.28 | 0.31 | 1.10 | 0.31 |
| *cyp19a1a* | 2.12 | 0.09 | 5.4 | **0.01** | 1.35 | 0.26 | 3.09 | 0.1 | 1.40 | 0.28 | 0.48 | 0.62 | 0.1 | 0.76 |

Additional File 2 Results from GLM analysis, components that are statistically significant are shown in bold type. Genes identified from the microarray are above the line.
