# Supplementary material for: Differential gene expression in male and female rainbow trout embryos prior to the onset of gross morphological differentiation of the gonads
Source: BMC Genomics. 2011 Aug 8;12:404. doi: 10.1186/1471-2164-12-404 (PMC3166948; doi:10.1186/1471-2164-12-404)
Supplement: Additional file 3 — Differences in expression between the sexes of the four candidate sex genes identifies from the microarray. Expression plots from four of the candidate sex determining genes identified from the microarray. [file 1471-2164-12-404-S3.DOCX]

Additional File 3. Expression plots from four of the candidate sex determining genes identified from the microarray.
