# Supplementary material for: Differential gene expression in male and female rainbow trout embryos prior to the onset of gross morphological differentiation of the gonads
Source: BMC Genomics. 2011 Aug 8;12:404. doi: 10.1186/1471-2164-12-404 (PMC3166948; doi:10.1186/1471-2164-12-404)
Supplement: Additional file 4 — Expression profile of candidate sex genes identified from Baron et al 2005. female/male ratio of candidate sex genes. Significant differences between the sexes within time points is shown by *, *= 0.05, ** = 0.01 [file 1471-2164-12-404-S4.DOCX]

Additional File 4, female/male ratio of candidate sex genes. Significant differences between the sexes within time points is shown by *, *= 0.05, ** = 0.01

*

**

*

*

*

*
