# Supplementary material for: Differential gene expression in male and female rainbow trout embryos prior to the onset of gross morphological differentiation of the gonads
Source: BMC Genomics. 2011 Aug 8;12:404. doi: 10.1186/1471-2164-12-404 (PMC3166948; doi:10.1186/1471-2164-12-404)
Supplement: Additional file 5 — Linkage mapping of candidate sex genes. linage groups with mapped candidate sex genes (in red). Maps were constructed from a double-haploid cross between two populations of O. mykiss, see Nichols et al (2008) for details. [file 1471-2164-12-404-S5.DOCX]

Additional File 5, linage groups with mapped candidate sex genes (in red). Maps were constructed from a double-haploid cross between two populations of O. mykiss, see Nichols et al (2008) for details.

LG27

LG22

LG6

LG9

LG18

LG10
