# Supplementary material for: Differential gene expression in male and female rainbow trout embryos prior to the onset of gross morphological differentiation of the gonads
Source: BMC Genomics. 2011 Aug 8;12:404. doi: 10.1186/1471-2164-12-404 (PMC3166948; doi:10.1186/1471-2164-12-404)
Supplement: Additional file 6 — Primer details from genes identified for RT-PCR analysis from the microarray. Primer details and PCR conditions for amplification of genes identified from the microarray [file 1471-2164-12-404-S6.DOCX]

Additional File 6, primer details and PCR conditions for amplification of genes identified from the microarray.

| gene name | Forward primer(5’🡪 3’) | Reverse Primer (5’ 🡪 3’) | PCR annealing temperature | Amplicon size (bps) | Amplification efficacy |
| --- | --- | --- | --- | --- | --- |
| vasa | GGACAGACACGCGTTACTCA | TGTGACGCATCACTTGAACTT | 58 | 158 | 80.2% |
| coatamer subunit | ATGGGGCTTACAGGTCAGAA | TGTTATATCATCTCTGCTCCAACG | 58 | 132 | 76.4% |
| prostaglandin | TGCAAGGTTTGGGTAAAGGT | AGGCAGCCTTCAAATCCTTC | 57 | 145 | 71.2% |
| zonadhesin | TGTACCATCTAAACCACTGTGAA | GCTCAAGTTTAGTTTTTGCGTCT | 58 | 134 | 85.8% |
| cyp19a1a | GGTACTGTGCACCTCCACCT | GAGACGGAAATGCAGCTCTT | 58 | 124 | 85.1% |
| PHB2 | gagcccagttctacgtggag | aggttgaggaccaggttgtc | 57 | 222 | 87.4% |
| tekt4 | tgtctcttcagcaggctctg | tgcgggactcctccaggtgg | 59 | 219 | 90.4% |
| STAB2 | gtgaaccagttccatctgagc | tcttagtagtcccacagtgctg | 56 | 205 | 83.2% |
| AhR2 | tggtcaggtgggtcatgctc | gcaaccaggtcaagtggtcatcc | 53 | 205 | 87.8% |
| COL10A1 | acaagaacgacaagccagtc | acaccgtatcacgttgaagc | 55 | 208 | 82.1% |
| CKB | ggcaagaccttcctggtgtg | tgagcacgtaacccaggtgc | 57 | 181 | 76.4% |
| kdelr2 | ttaggcccaggctcagcttg | cctctgtgacaaatgactgcatc | 52 | 205 | 79.8% |
| CP1 | tccagctctatgactcaggtg | tgtgcttgttcctggacatc | 55 | 183 | 74.6% |
| 1ELT | tggaactcaaacagcgttgc | acactgggcagccaagcctg | 58 | 212 | 83.6% |
| L-FABP | gaccacttcaaggtgactgtc | gtgttgacgagggtgtttgc | 57 | 200 | 86.4% |
| SLC31A1 | acacaaacgtggagctgctg | acagactcagcatccgctgg | 58 | 238 | 79.7% |
