# Supplementary material for: Differential gene expression in male and female rainbow trout embryos prior to the onset of gross morphological differentiation of the gonads
Source: BMC Genomics. 2011 Aug 8;12:404. doi: 10.1186/1471-2164-12-404 (PMC3166948; doi:10.1186/1471-2164-12-404)
Supplement: Additional file 7 — Primer details for mapping candidate sex genes. Marker, primers and PCR conditions used for mapping candidate sex genes, PCR annealing temperatures are given in °C. Note that primers used for zonadhesin were the same as those reported in Additional File 6. [file 1471-2164-12-404-S7.DOCX]

Additional File 7. Marker, primers and PCR conditions used for mapping candidate sex genes, PCR annealing temperatures are given in °C. Note that primers used for zonadhesin were the same as those reported in Additional File 6.

| gene | forward primer (5’🡪3’) | reverse primer (5’ 🡪 3’) | SNP genotyping primer (5’🡪 3’) | PCR annealing temperature |
| --- | --- | --- | --- | --- |
| *ovol1* | GCGTGGTTACACGTGGTCT | CAGGGGAAACACCTTGTGAC | GCTAGAGCATACAACAGTCAGTGTG | 58 |
| *FsT* | CCAGACTGCTCCAATGTCAC | TATGGATCTGCCCAGGAGAC | GAGTAAGTAAACCTGCAGTCCGATG | 58 |
| *cyp19a1a* | CTCTCCTCTCATACCTCAGGTT | AGAGGAACTGCTGAGTATGAAT | tgtctcctctccgttgatccagaccctgac | 58 |
| *wt1* | ATGCACCAGAGGAACCTGAC | ACTGAGAAATGGGCACACAA | atctcaatgccctgctgcccccggtgccatccttg | 57 |
| *Zonadhesin* |  |  | tccagctctatgactcaggtg | 60 |
| *foxl2b* | CGAGGAAGATTTAAACTACATG | atgcaggatccaaagtccag | acatgatcagatggacaaagaggac | 58 |
| *IGF1* | TGGACACGCTGCAGTTTGTGTGT | CACTCGTCCACAATACCACGGTT | tgtcagtgagtatcagtgtctgaga | 59 |
